# Advanced Imaging for Live-Cell Spatiotemporal Monitoring: Technologies and Applications

**DOI:** 10.34133/research.1085

**Published:** 2026-02-10

**Authors:** Zhong Zhuang, Zhichao Feng, Jie Wang, Xinhui Liu, Laijun Song, Chunhui Sun, Hong Liu, Na Ren

**Affiliations:** Collaborative Innovation Center of Technology and Equipment for Biological Diagnosis and Therapy in Universities of Shandong, Institute for Advanced Interdisciplinary Research (iAIR), School of Chemistry and Chemical Engineering, University of Jinan, Jinan 250022, P. R. China.

## Abstract

Understanding cellular dynamics requires real-time high-resolution imaging. Recent advancements in imaging technologies have provided unprecedented spatial and temporal resolutions, enabling the precise in situ monitoring of live-cell behavior. This review covers 4 advanced imaging modalities: stimulated emission depletion microscopy, structured illumination microscopy, single-molecule localization microscopy, and Raman spectroscopy. We summarize the principles, applications, advantages, and limitations of these methods, highlighting their significance for high-precision spatiotemporal monitoring of cellular structures and biochemical activities. These tools enable precise tracking of molecular interactions and analysis of cellular dynamics at the nanoscale, which is critical for understanding cellular physiology. The integration of these technologies into biomedical research has markedly enhanced our ability to observe live-cell processes, such as division, migration, differentiation, and signaling. The development and application of these high-precision imaging technologies hold substantial promise for improving disease diagnosis, therapeutic strategies, and drug discovery.

## Introduction

Understanding the dynamic and intricate behaviors of living cells is a goal of biological research. Cellular processes are complex and require advanced techniques to capture spatiotemporal dynamics with high precision and sensitivity. Emerging technologies for in situ spatiotemporal monitoring have significantly enhanced the ability to study living cells [1]. Unlike traditional experimental methods that often rely on fixed time-point analyses, in situ monitoring offers dynamic, real-time visualization of cells, allowing researchers to investigate processes such as proliferation, differentiation, and migration under physiological conditions [2]. Live-cell imaging, a groundbreaking development in this area, enables the continuous observation of cellular activities without disturbing the natural cellular environment [3]. Fluorescence microscopy, a core technique in live-cell imaging, has undergone major advances, delivering unprecedented spatial and temporal resolution. Using fluorescent probes and dyes, researchers can selectively visualize specific cellular components, track molecular interactions, and explore cellular dynamics at the single-cell level.

In recent decades, biomedical imaging has advanced remarkably, particularly with the advent of super-resolution (SR) techniques, profoundly enhancing our understanding of the life sciences [4]. Traditional optical microscopy is constrained by the diffraction limit, which restricts nanoscale observations. However, novel methods such as stimulated emission depletion (STED) microscopy [5], structured illumination microscopy (SIM) [6], single-molecule localization microscopy (SMLM) [7], and Raman spectroscopy [8] have overcome this barrier, providing unprecedented insights into cellular structure. STED microscopy uses a shaped depletion beam to confine fluorescence emission within a nanometric focal spot, surpassing the diffraction-limited resolution [9]. SIM uses specific optical illumination patterns to enhance the spatial resolution for detailed live-cell imaging [10]. SMLM achieves ultrahigh resolution by sequentially localizing individual fluorescent molecules and providing nanoscale structural details. Raman spectroscopy, although not an imaging technique, delivers detailed molecular fingerprints of intracellular components, offering a unique, label-free method for analyzing cellular biochemical composition and structures [11,12].

**Fig. 1.**
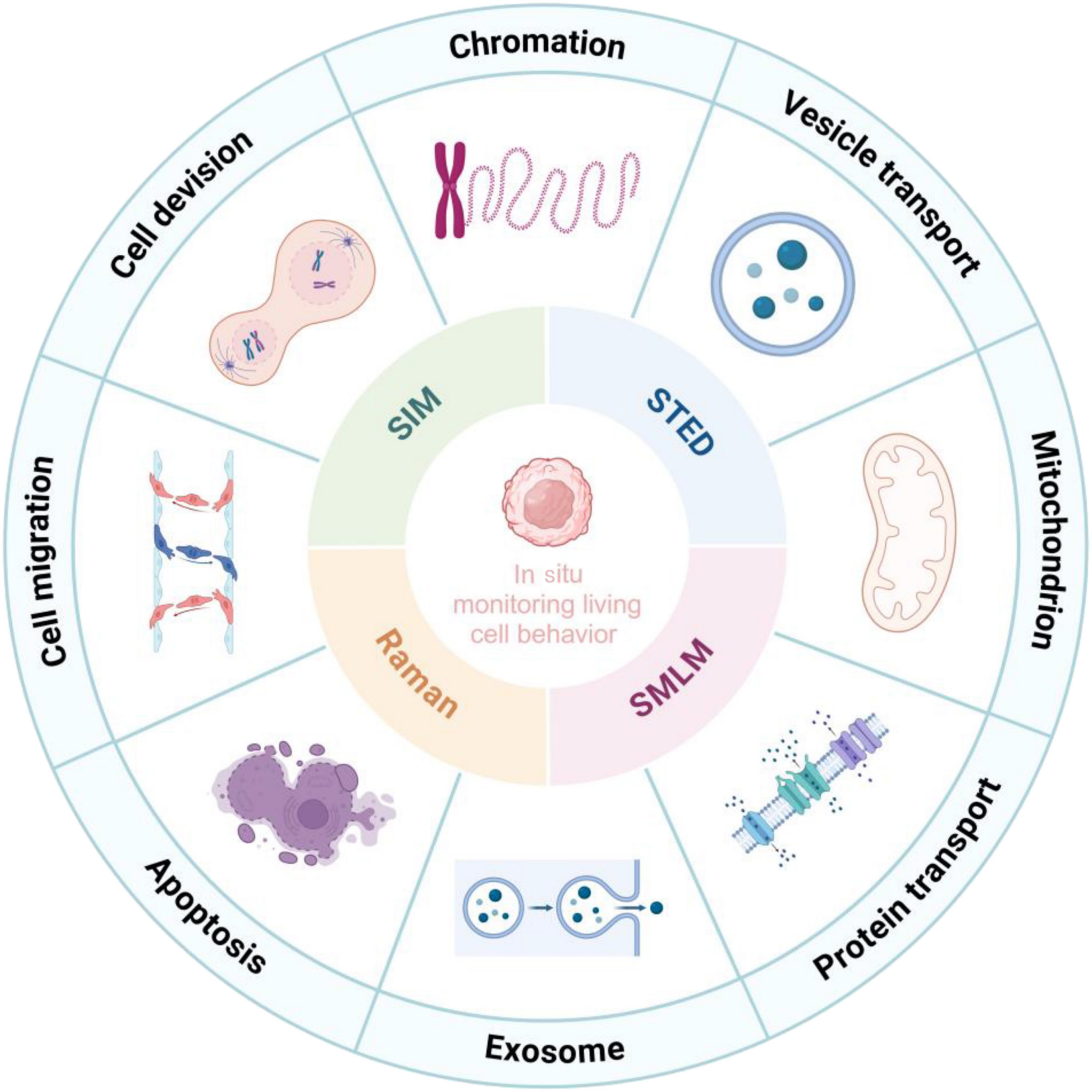
Advanced imaging techniques used for in situ monitoring of cellular behavior.

Collectively, these advanced imaging technologies have expanded our ability to observe and comprehend biological structures and functions, thereby providing powerful tools for disease diagnosis, therapeutic strategy development, and drug discovery. This review summarizes the principles, applications, advantages, and limitations of STED, SIM, SMLM, and Raman spectroscopy, aiming to guide biomedical researchers in selecting optimal imaging methods (Fig. 1). As these technologies evolve, continued innovations will undoubtedly deepen our understanding of life sciences, driving breakthroughs in biomedical research and medicine [13,14].

## STED Microscopy for Super-Resolved Live-Cell Imaging

**Fig. 2.**
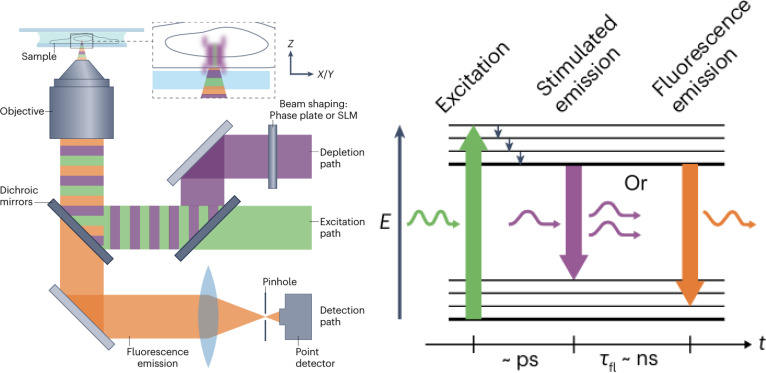
Principle of STED microscopy. Reproduction with permission [192]. Copyright 2022, Springer Nature.

STED microscopy has become an essential SR imaging technology that can overcome the resolution barrier of traditional optical microscopy [15]. This method combines a diffraction-limited excitation beam with a donut-shaped depletion beam (Fig. 2) [16]. The depletion beam induces stimulated emission at the periphery of the excitation spot, reducing the fluorescent region to nanoscale dimensions. STED microscopy achieves a lateral resolution of approximately 40 nm and an axial resolution of approximately 70 nm, allowing the detailed observation of live-cell structures at a high temporal resolution. The achievable resolution is influenced by the spectral properties of the fluorescent dyes and laser intensity.

Fluorescent markers suitable for STED microscopy must exhibit high photostability, as repeated excitation–depletion cycles are involved during imaging. Common fluorescent markers include membrane dyes, fluorescent proteins (FPs), synthetic fluorescent probes, carbon dots (CDs), and quantum dots (QDs). Although FPs conveniently label proteins through genetic fusion, their photophysical properties are not easily tunable, making them unsuitable for certain nanoscale imaging applications. In contrast, chemically synthesized fluorophores, such as Halo-Tag, acyl carrier protein, and SNAP-tag, generally provide superior photostability and quantum yields. The optical properties of these markers critically affect the performance of STED microscopy, thereby influencing image quality, resolution, and imaging depth [17].

### FPs for super-resolved live-cell imaging

The development of green FP (GFP)-based tools represents a significant breakthrough in live-cell microscopy, enabling direct visualization of target proteins through genetically encoded fusion constructs [18,19]. The known atomic structure of GFP allows researchers to link its structural features to its photophysical properties, facilitating rational chromophore tuning. In STED microscopy, FPs have notably advanced imaging, revealing cellular structures and molecular details within live samples at a nanometer resolution. Direct fusion of FPs to target molecules enables highly specific, in situ visualization of cellular structures and molecular interactions, providing insights into complex intracellular processes [20].

**Fig. 3.**
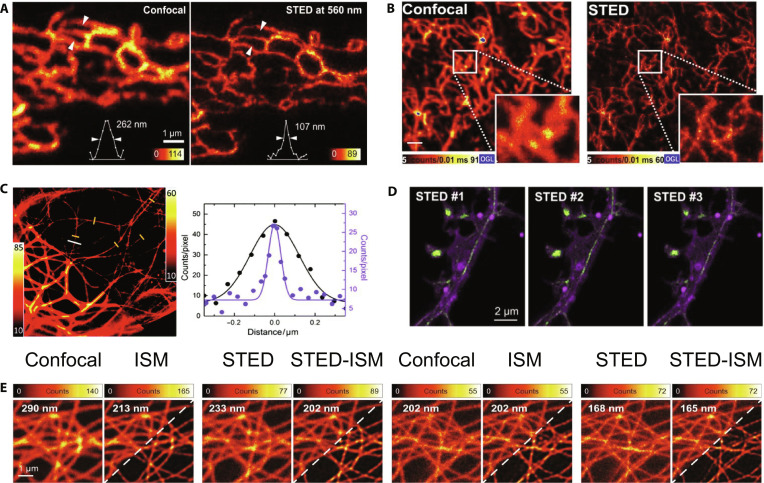
Imaging with FPs. (A) Live Vero cells expressing enhanced GFP (eGFP) in the ER, measured using a 560-nm STED wavelength. Reproduction with permission [21]. Copyright 2011, Elsevier. (B) Subdiffraction resolution STED imaging (right) of vimentin fused with hAGT and labeled TMR in live PtK2 cells, compared with the confocal image (left). Scale bar, 1 μm. Reproduction with permission [23]. Copyright 2010, Elsevier. (C) STED imaging of mGarnet2-labeled microtubules in live COS-7 cell. Left: Combined confocal (bottom left) and STED (top right) image. Right: Representative cross-section of microtubules in confocal (black symbols) and STED (purple symbols) images, marked by white lines. Reproduction with permission [24]. Copyright 2017,The Royal Society of Chemistry. (D) Dual-labeled STED microscopy images of actin staining with RSFPs Padron (green, Lifeact) and membrane with Dronpa-M159T (magenta, myristoylation) in living cultured neurons recorded at 3 time points. Reproduction with permission [25]. Copyright 2021, Elsevier. (E) Comparison of confocal, STED, STED-ISM (left, top corner), and multi-image deconvolution STED-ISM^+^ (right, bottom corner) images of live HeLa cells labeled with silicon rhodamine tubulin. Reproduction with permission [26]. Copyright 2022, Springer Nature.

In 2011, Rankin et al. [21] demonstrated the use of STED microscopy in live *Caenorhabditis elegans* that expressed GFP-fusion proteins. By using pulsed and continuous-wave depletion beams at 556 to 592 nm, the team obtained subdiffraction images within seconds, allowing real-time visualization of dynamic endoplasmic reticulum (ER) structures (Fig. 3A). Similarly, Westphal et al. [22] fused organic fluorophores to synaptic vesicles to achieve video-rate imaging at subdiffraction resolutions. These results provide a powerful approach for exploring dynamic cellular processes. Human *O*^6^-alkylguanine–DNA alkyltransferase (hAGT) is one of the smallest labeled proteins available and exhibits exceptional specificity for covalent linkages with diverse fluorophores. In 2010, Hein et al. [23] utilized hAGT covalently tagged with tetramethylrhodamine (TMR) to image cytoskeletal structures and cell membranes at resolutions of up to 40 nm. Their results showed significantly improved clarity of the vimentin filament network compared to conventional confocal microscopy (Fig. 3B), highlighting hAGT’s utility of hAGT in live-cell imaging.

Advances in far-red FPs are important for imaging live cells, tissues, and organisms. Matela et al. [24] engineered mGarnet2, a far-red FP with absorption at 598 nm and emission at 671 nm, and achieved a 4.2-fold resolution improvement in STED microscopy, from approximately 200 to 69 nm. This allowed for detailed visualization of actin, microtubules, mitochondria, and nuclei within live cells (Fig. 3C). In 2021, Willig et al. [25] developed an in vivo STED system integrating spectrally isolated excitation and detection channels with synchronized toggling of reversible switchable FPs (RSFPs). Their approach simplified the optical setup and resolved nanostructures within mouse cortical neurons under triple-labeling conditions (Fig. 3D). Imaging thicker samples using STED microscopy faces challenges, including photodamage and background fluorescence. To address this, Tortarolo et al. [26] introduced STED image scanning microscopy (STED-ISM), which combines detector arrays and reduces the intensity of the depletion beam. Their focus ISM strategy further enhanced optical sectioning, significantly improving the resolution and reducing the background noise, as demonstrated by imaging microtubules in living HeLa cells (Fig. 3E).

The integration of FPs into STED microscopy has greatly advanced our ability to visualize intracellular dynamics, promoting both fundamental and applied research in life sciences. Nevertheless, challenges such as the limited photostability and suboptimal brightness of FPs restrict their use in prolonged imaging and experiments that require high photon budgets. Recent studies have emphasized the need to engineer FPs with improved photostability, quantum yield, and far-red to near-infrared (NIR) emission spectra to enable extended multicolor live-cell imaging with minimal photobleaching and phototoxicity. Furthermore, the inherent limitations of STED microscopy, particularly the photodamage caused by high-intensity depletion lasers and the complexity of optical alignment, have spurred technological innovations such as time-gated detection, adaptive optics, and event-triggered or artificial intelligence (AI)-assisted imaging, all of which are designed to reduce illumination intensity and enhance cell viability during long-term imaging.

### Fluorescent probes for super-resolved live-cell imaging

Unlike genetically encoded fluorophores, fluorescent probes are chemically synthesized molecules specifically designed to label and detect targeted intracellular structures or molecules. These probes function by absorbing photons of specific wavelengths and subsequently emitting fluorescence upon relaxation to their ground states. Their tunable photophysical characteristics offer significant advantages for nanoscopic imaging techniques such as STED microscopy [27,28].

**Fig. 4.**
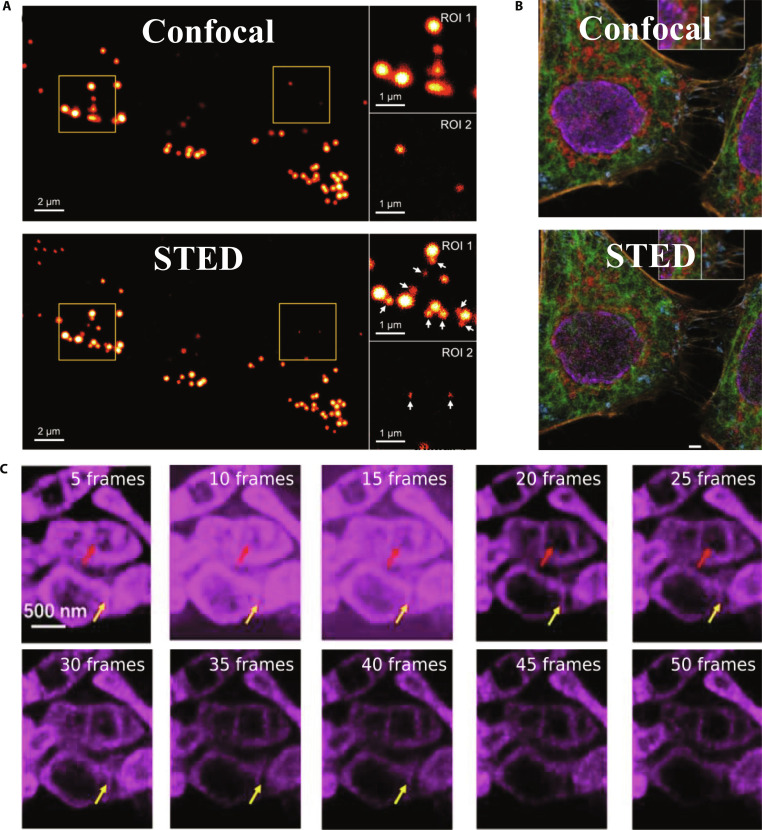
STED imaging with fluorescent probes. (A) Comparison between confocal and STED nanoscopic images of HeLa cells labeled with Lipi-BDTO; the enlarged region is shown in the inset. Reproduction with permission [29]. ROI, region of interests. Copyright 2021, American Chemical Society. (B) Five-color FLIM-STED phasor-separated and FLIM confocal images with the same pixel size. The inset shows 2 regions magnified by a factor of 2. Scale bar, 2 μm. Reproduction with permission [31]. Copyright 2017, Springer Nature. (C) Mitochondrial dynamics images from different frames labeled with TPERh using STED. Red and yellow arrows indicate mitochondrial fission. Reproduction with permission [32]. Copyright 2024, Optica Publishing Group.

In 2021, Liu et al. [29] introduced Lipi-BDTO, a benzodithiophene-tetraoxide-based fluorescent probe that was optimized for STED microscopy. Owing to its excellent photostability and compatibility with STED imaging conditions, Lipi-BDTO enabled the precise visualization of lipid droplets (LDs) at resolutions as low as 65 nm, outperforming conventional confocal microscopy (Fig. 4A). Concurrently, Liang et al. [30] developed neodymium-based rare-earth nanoparticles as STED nanoprobes, achieving enhanced contrast imaging at depths of up to 50 μm within immunostained HeLa cells. In 2022, Gonzalez Pisfil et al. [31] combined single-depletion laser STED with fluorescence lifetime imaging microscopy (FLIM) to simultaneously resolve 5 different fluorophores. This approach significantly expanded color differentiation in STED imaging (Fig. 4B). In 2024, Gao et al. [32] designed a tetraphenylethene-functionalized rhodamine dye (TPERh) that exhibited remarkable resistance to photobleaching and low saturation power requirements. TPERh enabled the visualization of mitochondrial cristae dynamics in living cells at a resolution of approximately 84 nm (Fig. 4C), which is valuable for studying mitochondrial biology.

Fluorescent probes have markedly expanded the utility of STED microscopy by enabling the highly specific nanometric visualization of live-cell structures relevant to disease, drug discovery, and neuroscience. Recent probe engineering has focused on lower saturation power and higher photostability, allowing for longer time-lapse STED at reduced light doses. For example, mitochondrial probes such as HBmito Crimson and TPERh sustain hundreds of frames and maintain cristae contrast under low-power depletion, while supporting functional readouts such as membrane-potential monitoring in live cells [33]. Meanwhile, extending fluorophore emission into the far-red and NIR ranges, together with nanoparticle-based probes, enhances multiplexing and imaging depth, whereas lifetime-domain techniques, such as FLIM-STED phasor analysis, minimize spectral overlap and enable multicolor imaging with a single depletion laser [34]. Despite these advances, practical limitations persist. Some organic probes still raise cell-entry/biocompatibility concerns in specific models, and even improved dyes can photobleach under harsh conditions. Ongoing priorities include next-generation rhodamine/JF-style scaffolds and self-healing dye concepts to further increase the brightness/photostability while lowering the required dose, along with expanding far-red/NIR palettes for deep and multiplexed STED [35]. In parallel, the development of low-power nanoprobes provides an additional strategy for achieving high-resolution imaging. Guo et al. [36] demonstrated low-power single-wavelength-pair nanoscopy using NIR-II continuous-wave lasers and lanthanide-doped upconversion nanoprobes, achieving sub-100-nm resolution and multicolor imaging at photon doses nearly an order of magnitude lower than those of conventional STED microscopy. These NIR-responsive nanoprobes exhibit large anti-Stokes shifts and long-lived excited states, offering efficient depletion under minimal irradiation and extending STED microscopy toward low-phototoxicity, long-term biological imaging.

### CDs and QDs for super-resolved live-cell imaging

**Fig. 5.**
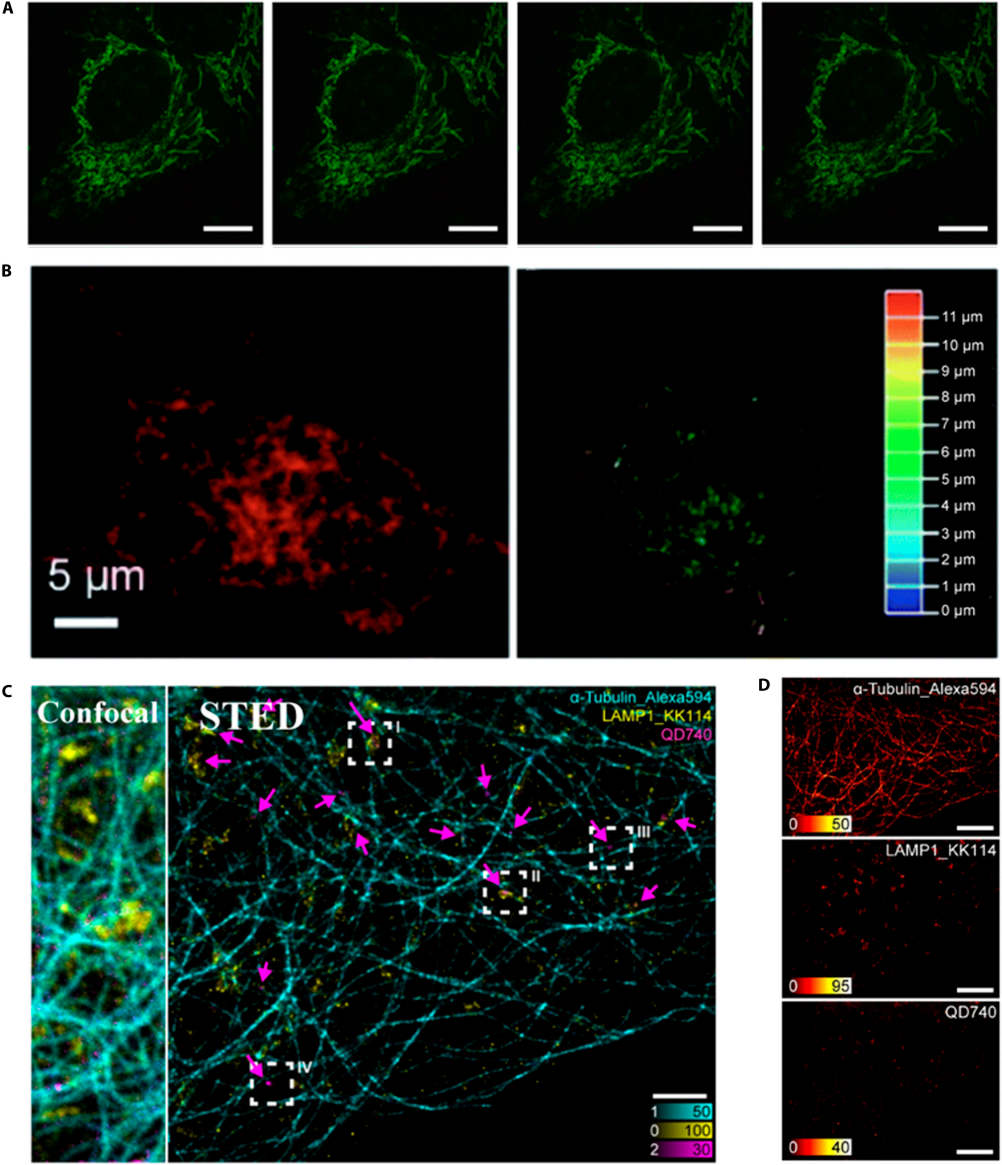
Cellular Imaging with CDs and QDs. (A) STED nanoscopic image of HeLa cell samples labeled with carbon nanodots. Scale bars, 10 μm. Reproduction with permission [39]. Copyright 2023, Elsevier. (B) STED image of ER in HeLa cells and the corresponding 3D reconstruction of the ER. Reproduction with permission [40]. Copyright 2022, The Royal Society of Chemistry. (C) Three-color STED imaging of lysosome-associated membrane protein 1 (yellow), α-tubulin (cyan), and QD740 (magenta) in U2OS cells. Scale bar, 2 μm. (D) Channels from (C). Scale bars, 5 μm. Reproduction with permission [43]. Copyright 2023, Wiley-VCH GmbH.

CDs are a promising class of fluorescent nanomaterials characterized by their excellent biocompatibility, stability, and superior optical properties [37]. Their compact size and favorable photostability make them ideal for SR techniques, such as STED microscopy. Leménager et al. [38] demonstrated CDs’ utility of CDs in STED, achieving 30-nm resolution in live cells and confirming their biocompatibility and imaging potential. Li et al. [39] later developed nitrogen-doped CDs targeting mitochondria and lysosomes, achieving resolutions of approximately 134 and 55 nm, respectively (Fig. 5A). Li et al. [40] designed amphiphilic CDs (Phe CDs) to overcome photobleaching limitations, facilitate ER imaging at sub-100-nm resolution, and allow detailed visualization of ER dynamics during cell division (Fig. 5B).

QDs are semiconductor nanocrystals known for their brightness and resistance to photobleaching. Their optical properties make them particularly suitable for STED microscopy, enabling the SR visualization of intracellular structures. For example, Hanne et al. [41] achieved a resolution of approximately 50 nm with red-emitting QDs, resolving the vimentin filaments. Subsequently, Wang et al. [42] applied Mn-doped ZnSe QDs to improve the imaging clarity of microtubules at a resolution of approximately 85 nm. In 2023, Alvelid et al. [43] used CdTe QDs to achieve streamlined 3-color STED imaging, significantly simplifying multicolor imaging setups (Fig. 5C and D).

Despite recent advances, QDs face significant limitations preventing their broader use in SR microscopy [44]. First, high production costs due to complex synthesis and purification restrict accessibility, particularly for large-scale research. Developing cost-effective, streamlined synthesis methods is therefore essential. Second, batch-to-batch variations in size, shape, and surface chemistry often cause inconsistent photophysical properties (e.g., emission wavelength and quantum yield), compromising imaging reproducibility. Future efforts must focus on standardized protocols to ensure optical uniformity. Third, cytotoxicity concerns stemming from heavy-metal components limit live-cell and clinical applications, necessitating the development of heavy-metal-free QDs or robust biocompatible coatings. Finally, fluorescence instability during extended imaging hampers continuous monitoring; this can be addressed by engineering core–shell structures or hybrid nanoparticles to enhance photostability [45,46]. In contrast, CDs offer superior biocompatibility and stability but often suffer from lower brightness and resolution. Future research should prioritize enhancing their quantum yields and refining surface modifications. In this context, innovative material designs are emerging. For instance, Kang et al. [47] reviewed luminescent biomass nanocomposites with programmable emission. Although primarily designed for anticounterfeiting, such low-toxicity, cost-effective biomass platforms represent a promising avenue for next-generation biocompatible imaging probes. Ultimately, addressing these challenges through targeted material design and innovative synthesis will significantly expand the utility of QDs and CDs in SR microscopy, providing deeper insights into cellular dynamics.

Table 1 summarizes the advantages and limitations of the different types of fluorescent markers used in STED microscopy, offering guidance and references for researchers.

**Table 1.** Advantages and limitations of fluorescent indicators for STED imaging in living cells

Technique	Applications	Advantages	Limitations	Reference
FPs	Labeling specific proteins or cellular structures	No additional staining steps required, long-term live-cell imaging	Low brightness and photostability of some FPs for STED imaging	[194]
Fluorescent probes	Specific detection of intracellular environments	High brightness and specificity, labeling specific molecules and structures	Cytotoxicity and requirement for cell membrane permeation	[195]
CDs	Fluorescent markers for bioimaging	Good biocompatibility, high photostability	Relative low resolution and brightness	[196]
QDs	Multicolor imaging and dynamic intracellular processes tracking	High brightness and photostability	Challenges in biocompatibility	[197]

### AI-enhanced STED microscopy: Toward adaptive and low phototoxic

The integration of AI and deep learning has transformed STED microscopy from a hardware-limited technique into an intelligent imaging framework. Deep neural networks enable high-quality SR imaging under ultralow irradiation by performing denoising, image reconstruction, and resolution enhancement, thereby minimizing photobleaching and phototoxicity [48]. Architectures such as U-shaped convolutional neural network, convolutional neural networks (CNNs), and generative adversarial networks (GANs) have been successfully applied to accelerate image acquisition [49], reconstruct 3-dimensional (3D) structures, and extend the live-cell imaging duration. Simulation platforms, such as pySTED, provide physically realistic datasets for training and benchmarking AI models, thereby addressing data scarcity and reproducibility challenges. Representative studies have demonstrated the power of this integration. Balakrishnan et al. [50] developed a neural-network-assisted low-irradiation STED approach that enables long-term, fast SR imaging of organelle dynamics, such as the ER, with minimal phototoxicity, whereas Rahm et al. [51] combined ultralow-dose STED with deep learning restoration to achieve quantitative, second-resolution live-cell imaging over several hours. Similarly, Bilodeau et al. [52] introduced the pySTED platform, a physics-based simulator that supports deep and reinforcement learning model training, online optimization, and seamless simulation-to-real deployment without the need for fine-tuning. Together, these advances establish a solid foundation for the evolution of AI-driven STED microscopy toward more adaptive, quantitative, and biologically compatible imaging paradigms.

Building on these advances, the incorporation of AI and deep learning has propelled STED microscopy beyond hardware limitations toward an intelligent, data-driven imaging paradigm. Deep neural networks, such as U-Net, Residual Channel Attention Network and Generative Adversarial Network architectures, enable SR imaging under ultralow irradiation by performing denoising, image reconstruction, and resolution enhancement, thereby markedly reducing photobleaching and phototoxicity while preserving the nanoscale structural fidelity. AI-enhanced STED systems are expected to achieve adaptive and quantitative imaging through reinforcement-learning-based beam control and Bayesian optimization, which enable closed-loop regulation of excitation and depletion intensities for real-time adaptive imaging at minimal light doses and enhanced cellular viability [53]. Integrating physical priors, including point-spread functions, photobleaching kinetics, and photon statistics, within deep learning architectures can effectively suppress hallucination artifacts and maintain temporal consistency during prolonged imaging, as demonstrated by emerging physics-informed restoration frameworks. The establishment of standardized paired datasets linking low- and high-dose STED acquisitions with dynamic cellular annotations is critical for the cross-platform validation of AI models. In parallel, lifetime-domain strategies, such as FLIM-STED phasor analysis combined with far-red and NIR fluorophores, are expanding multicolor (>4 to 5 channels) and deep-tissue imaging capabilities under low phototoxic conditions. Collectively, these advances position next-generation AI-driven STED microscopy as a self-optimizing and quantitatively reliable platform for the long-term visualization of cellular and subcellular dynamics with molecular precision [54].

### Applications of STED microscopy technology for investigation of live-cell behavior with high-resolution imaging

STED microscopy has significantly advanced live-cell imaging and has become an indispensable tool in cell biology and neuroscience [55]. Its nanoscale resolution enables researchers to rapidly visualize intracellular events and detailed subcellular structures. In cytoskeletal studies, STED microscopy has provided critical insights into cellular motility and morphological changes by precisely tracking microtubule and actin filament dynamics. Moreover, it excels in membrane biology, revealing the organization and interactions of membrane proteins, thus deepening our understanding of signaling pathways and transmembrane transport [56].

In 2018, Kamper et al. [57] introduced a small NIR FP (SNIFP) that significantly enhanced the resolution of STED microscopy in the range of 650 to 900 nm. Compared with conventional confocal microscopy, SNIFP markedly improves the imaging of centromere proteins, vimentin filaments, and microtubule-associated proteins, enabling detailed capture of intracellular dynamics.

**Fig. 6.**
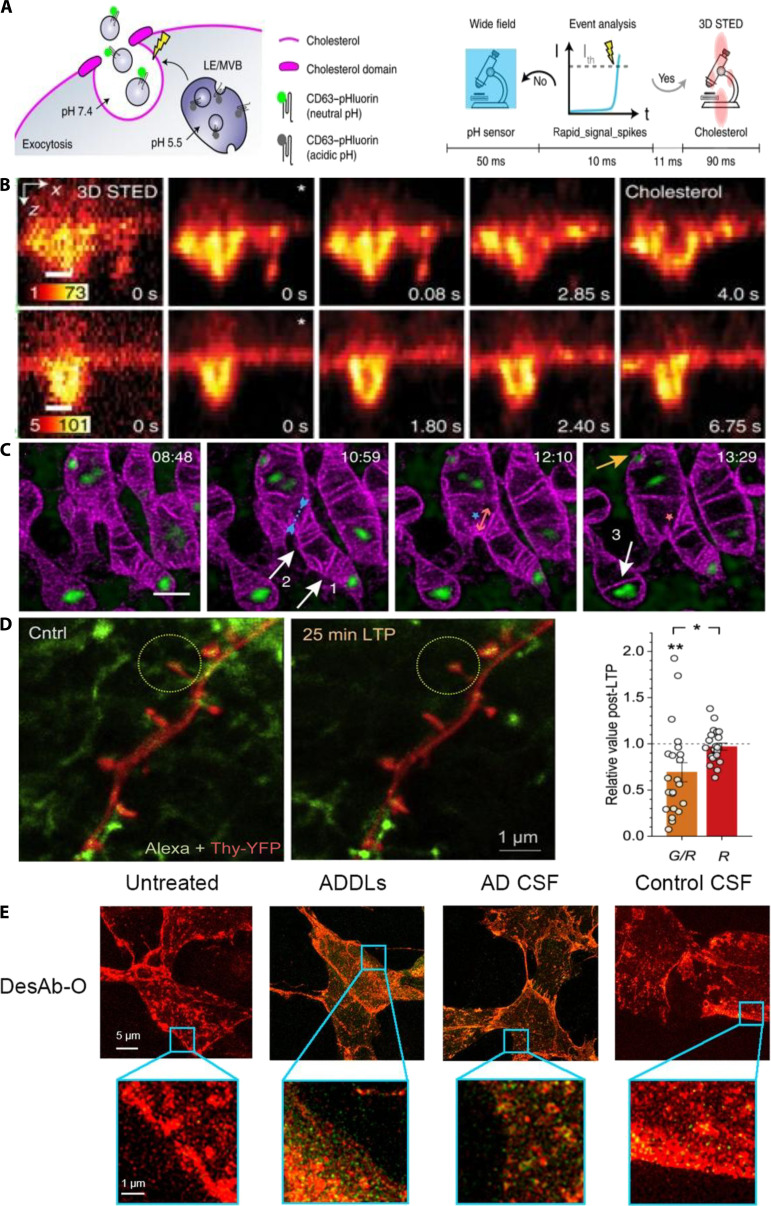
Applications of STED microscopy in cellular behavior studies. (A) Schematic representation of exocytosis and the experiment timeline of one WF frame. LE, late endosome; MVB, multivesicular body. (B) Triggered 11-Hz 3D STED time lapses of cholesterol-labeled plasma membrane dynamics and accumulation. Scale bars, 250 nm. Reproduction with permission [58]. Copyright 2022, Springer Nature. (C) Cristae remodeling and mtDNA convergence during early apoptosis. Scale bar, 1 μm. Reproduction with permission [33]. Copyright 2024, Springer Nature. (D) STED image of dendritic spines (red) and nearby astrocytes (green). Right: Reduced green/red (astrocyte/neuron) pixel ratios within the region of interests after LTP induction. *P < 0.02 compared to G/R change. Reproduction with permission [66]. Copyright 2020, Springer Nature. (E) STED images of Aβ42 oligomers present in the cerebrospinal fluid of patients with Alzheimer’s disease. DesAb, designed antibody; O, oligomer. Reproduction with permission [68]. Copyright 2020, Springer Nature.

Effective monitoring of intracellular proteins and lipids requires enhanced spatiotemporal resolution. However, photobleaching often limits the use of STED microscopy in prolonged live-cell studies. To overcome this problem, Alvelid et al. [58] developed an event-triggered STED (etSTED) microscope in 2022. This automated technique initiates rapid 2D and 3D STED imaging upon the detection of cellular events such as protein recruitment, vesicle transport, and second messenger signaling. By capturing synaptic vesicle dynamics at 24 Hz and endocytosis or exocytosis events at 11 Hz, etSTED significantly expanded real-time nanoscale imaging capabilities (Fig. 6A and B). In 2020, Barbotin et al. [59] applied axial STED microscopy to study plasma membrane dynamics, achieving a lateral resolution of approximately 35 nm. This allowed the visualization of previously undetectable structures, including endocytic vesicles and fine tubular membrane networks. The enhanced axial resolution (~100 nm) distinguished closely spaced structures along the *z* axis.

Mitochondria contain complex internal structures, such as cristae and mitochondrial DNA (mtDNA), which are vital for cellular metabolism and function. However, traditional optical microscopy cannot detect these nanoscale features [60]. STED microscopy effectively images the mitochondrial cristae and mtDNA, facilitating the study of their dynamic interactions [61]. In 2020, Yang et al. [62] introduced the optimized fluorescent probe MitoESq-635, which enables continuous STED imaging of mitochondrial dynamics at a resolution of approximately 35 nm. This revealed detailed changes during mitochondrial fusion and fission. Ren et al. [33] further demonstrated high-resolution imaging of mitochondrial cristae and their dynamic interactions with mtDNA during apoptosis (Fig. 6C), offering valuable insights into the mechanisms of mitochondrial regulation.

In neuroscience, STED microscopy has profoundly affected our understanding of the neuronal ultrastructure, synaptic transmission, and neural plasticity. By capturing rapid dynamics of synaptic vesicles and postsynaptic densities, STED microscopy provides critical insight into neuronal activity and information processing by capturing the rapid dynamics of synaptic vesicles and postsynaptic density. In addition, it has advanced research on axonal transport and dendritic spine morphology, shedding new light on the pathology of neurological diseases [63,64].

In 2020, Arizono et al. [65] used 3D-STED microscopy to image astrocytes in live brain slices, revealing detailed nanoscale structures such as nodal and shaft regions. Their findings demonstrated that nodal regions are important biochemical compartments and calcium microdomains that are crucial for synaptic regulation. Similarly, Henneberger et al. [66] used STED microscopy to visualize astrocytic processes and glutamate distribution at ultrahigh resolution and observed significant morphological changes in astrocytes during long-term potentiation (LTP). These observations highlight the dynamic role of astrocytes in synaptic transmission and plasticity (Fig. 6D).

At the clinical translation frontier, STED is evolving from a research imaging tool to a quantifiable nanopathology. Nanoscale spatial fingerprints can serve as novel biomarkers for early cancer diagnosis, molecular stratification, and therapeutic monitoring, particularly for events such as receptor clustering. Bergstrand et al. [67] used STED nanoscopy to reveal cancer-induced circular P-selectin nanostructures in platelets and converted these patterns into diagnostic biomarkers for classification, therapy monitoring, and relapse surveillance. Beyond oncology, STED resolves ultrastructural changes in pathogen protein assembly and amyloid deposition in neurodegenerative diseases, providing a quantitative and reproducible evidence base for etiological studies and companion diagnostics (Fig. 6E) [68]. Johansson et al. [69] STED resolves interwoven amyloid-β (Aβ) fibrils at ~29 nm (in vitro) and ~62 nm (tissue) in 3×Tg-AD mouse brain, delivering 5 to 10× better resolution than confocal and electron microscopy (EM)-like structural detail.

STED microscopy has advanced live-cell imaging and has become an indispensable technique for exploring nanoscale intracellular dynamics with exceptional precision. Overcoming the traditional optical resolution limitations has provided critical insights into diverse cellular processes, including cytoskeletal remodeling, membrane organization, mitochondrial interactions, and neuronal ultrastructure. Innovations such as SNIFP, etSTED, and axial STED have markedly enhanced spatial and temporal resolution, facilitating detailed observations of previously undetectable molecular and structural events. Despite notable achievements, challenges such as photobleaching, phototoxicity, and technical complexity persist, emphasizing the need for ongoing innovation in fluorescent probe development, optical instrumentation, and imaging methodologies. Continued advances in these areas promise to further expand the capabilities of STED microscopy, deepen our understanding of fundamental cellular mechanisms, and significantly accelerate progress in biomedical research and clinical translation.

## Structured Illumination Microscopy for Live-Cell Imaging

SIM significantly enhances image resolution using specialized illumination patterns combined with computational postprocessing [70]. Its principle involves the projection of a series of high-frequency grating patterns onto a sample. By varying the pattern orientations and phases, images collected from multiple angles were reconstructed using Fourier transforms, yielding images with greater detail than those obtained using conventional methods. SIM allows the visualization of dynamic cellular processes with minimal phototoxicity and wide-field (WF) observation. SIM techniques include optical sectioning SIM (OS-SIM) for enhanced axial resolution, 2D-SIM for improved lateral resolution, and 3D-SIM for improved combined axial and lateral resolution. Both 2D-SIM and 3D-SIM are SR-SIM [71–73].

Recently, deep learning methods, particularly CNNs, have significantly enhanced SIM data processing speed and accuracy of SIM data processing. These advances have markedly improved imaging efficiency and quality, expanded SIM’s biomedical applications of SIM, and enabled researchers to explore cellular and tissue structures with greater precision [74].

### OS-SIM for live-cell imaging

OS-SIM enhances imaging resolution using oblique illumination [75]. By acquiring 3 phase-shifted raw images and mathematically recombining them, OS-SIM generates optical sections with superior contrast and resolution compared with conventional WF imaging. This method effectively reduces background noise and is beneficial for imaging thicker biological samples. OS-SIM offers higher spatial resolution without compromising temporal resolution, making it suitable for the real-time observation of rapid cellular events [76]. Furthermore, OS-SIM enables multicolor imaging, facilitating the simultaneous visualization of multiple biomolecules and cellular structures.

**Fig. 7.**
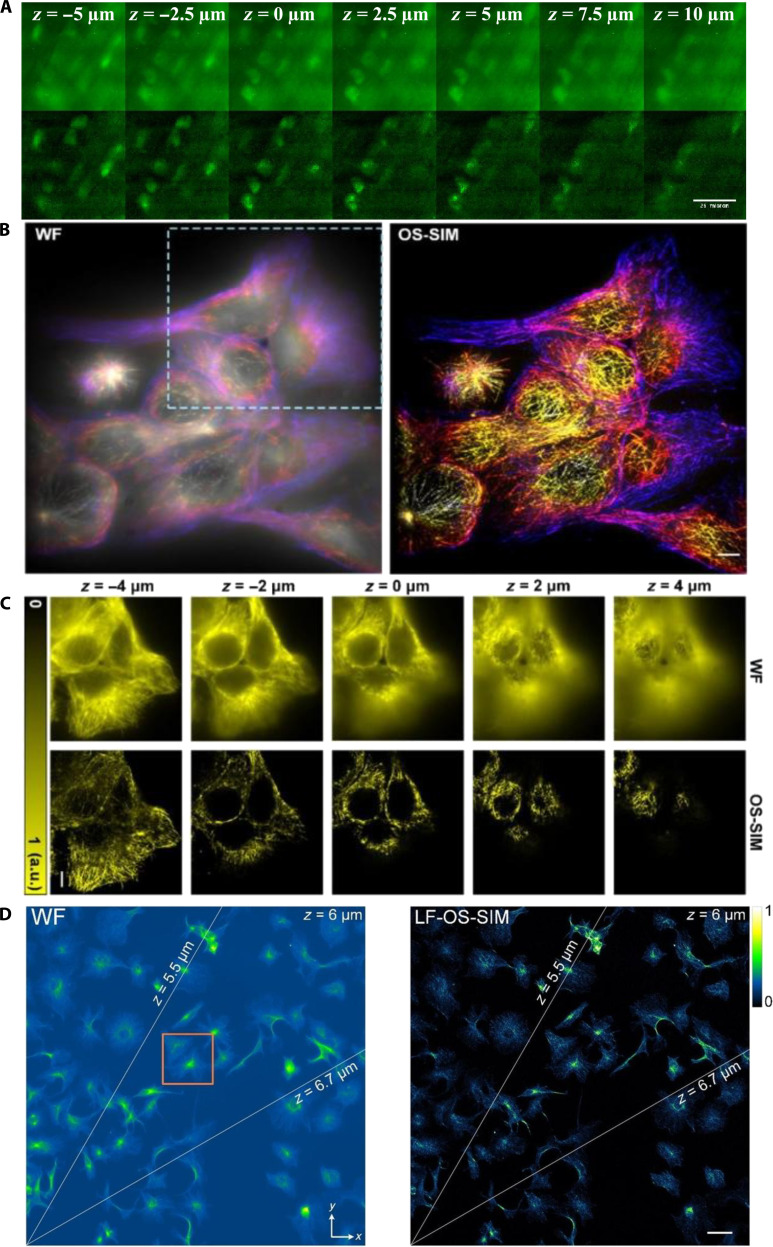
OS-SIM cellular imaging. (A) Comparison between pseudo-WF (top) and OS-SIM (bottom) z-stacks images of brain slices captured with a step size of 2.5 μm. Scale bar, 25 μm. Reproduction with permission [78]. Copyright 2023, Optica Publishing Group. (B) 3D fluorescence imaging of fixed MCF-10A cells labeled for microtubules with Alexa Fluor 532 using wide field and OS-SIM. Scale bar, 5 μm. (C) Five *xy* sections at 2-μm axial intervals with intensity color coding. Scale bar, 5 μm. Reproduction with permission [79]. Copyright 2025, Wiley-VCH GmbH. (D) WF and LF-OS-SIM images of microtubules in fixed mouse stem cells, showing results at *z* = 5.5, 6.0, and 6.7 μm. Reproduction with permission [80]. Copyright 2025, Elsevier.

Förster resonance energy transfer (FRET) microscopy has also benefited from advancements in OS-SIM. In 2022, Liu et al. [77] applied OS-SIM to quantitatively image FRET in live HeLa cells expressing FRET fusion proteins. OS-SIM provides a spatial resolution of approximately 200 nm, comparable to that of confocal microscopy, thus improving the precision of live-cell molecular interaction studies. Conventional OS-SIM systems rely on complex illumination setups, such as spatial light modulators or digital micromirror devices. To simplify miniaturization, Kumar et al. [78] introduced a micro-light-emitting diode (microLED)-based OS-SIM in 2023. Testing of GFP-labeled oligodendrocytes in mouse brain slices demonstrated a significantly improved optical sectioning contrast (86.92%) compared to pseudo-WF imaging (44.31%). These results highlight the potential of the microLED-based OS-SIM for deep-tissue WF imaging (Fig. 7A). In 2025, Li et al. [79] integrated partially coherent illumination into OS-SIM to axially confine the fringe modulation and enhance the axial resolution. Systematic theory and experiments varying the modulation period and illumination angular spectrum showed a ~5× improvement for scattering samples and a 1.4× improvement for fluorescent samples over conventional OS-SIM. Imaging of immunostained microtubules in fixed cells resulted in a fivefold increase in SBR compared to WF imaging (Fig. 7B). These results clarify the sectioning mechanism and provide guidelines for illumination design in 3D biological imaging. In 2025, Chen et al. [80] demonstrated large-field OS-SIM (LF-OS-SIM), which uses a 1D grating for stripe generation and a spatial light modulator for Fourier-domain phase shifting, thereby increasing the spatial bandwidth product by 2.6-fold over conventional OS-SIM. Validated on Eurocoins and fluorescent samples, the LF-OS-SIM provides a large field of view with rapid phase shifting. When applied to β-tubulin-labeled microtubules in fixed mouse stem cells (Fig. 7C), it effectively suppressed background noise and yielded optically sectioned images that were superior to WF imaging, underscoring its potential for 3D imaging of industrial microdevices and biological specimens.

OS-SIM has markedly advanced the imaging of complex biological specimens by delivering enhanced spatial resolution and effective background suppression, making it particularly suitable for multicolor visualization and studies of thicker tissues. Nevertheless, certain limitations persist, including dependence on specialized illumination patterns, which complicate the optical setup and restrict its integration into compact or simplified microscopy platforms. Furthermore, OS-SIM’s imaging speed of OS-SIM, which is faster than that of traditional SIM methods, still faces constraints in capturing rapid cellular dynamics. Future developments should prioritize simplifying illumination strategies, such as utilizing compact modulators (such as microLEDs), optimizing computational reconstruction methods, and enhancing temporal resolution to unlock OS-SIM’s potential of OS-SIM in real-time, high-resolution imaging of living biological systems.

### 2D-SIM and 3D-SIM for live-cell imaging

2D-SIM utilizes specific grating patterns projected onto samples, enhancing the lateral resolution through alterations in pattern orientation, phase, and combined image reconstruction from multiple angles [81]. It achieved a lateral resolution of approximately 100 nm, which is ideal for thin samples requiring high resolution. In contrast, 3D-SIM changes the focal plane, in addition to the pattern direction and phase, allowing the reconstruction of images at various sample depths. Advanced algorithms provide detailed 3D structural information, significantly improving the resolution in all spatial dimensions and, thus, enabling the visualization of complex cellular structures [82].

**Fig. 8.**
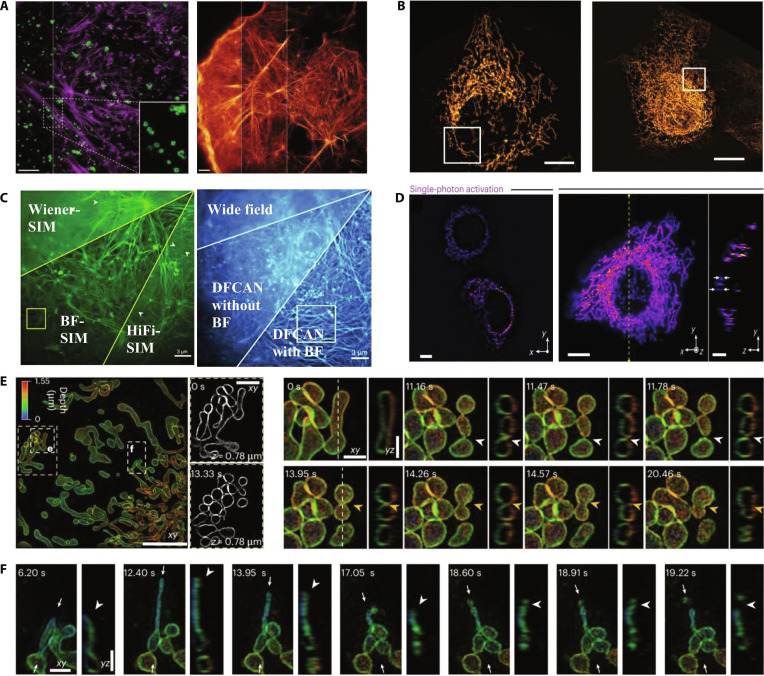
2D- and 3D-SIM cellular imaging. (A) Resolution improvement progress imaging of cortical actin (left) and cellular actin cytoskeleton (right). Scale bars, 2 μm. Reproduction with permission [84]. Copyright 2015, American Association. (B) Live U2OS cells imaged with POPSIM at a total rate of 2.2 Hz (left). Magnified view of the boxed region in (A) as imaged with projection Oblique Plane Microscopy (top right) and POPSIM (bottom right). Reproduction with permission [86]. Copyright 2023, Springer Nature. (C) Representative examples of COS-7 cells reconstructed using traditional Wiener-SIM, High-fidelity structured illumination microscopy, and BF-SIM (left) and the SR image of HiFi-SIM and BF-SIM (right). Reproduction with permission [87]. Copyright 2023, Springer Nature. (D) SPA-SIM imaging of the mitochondria in live HeLa cells. 2D plane images (left), 3D rendered images (right), and vertical cross-sectional images along the vertical yellow dashed line. Scale bars, 10 μm. Reproduction with permission [88]. Copyright 2024, Springer Nature. (E) Imaging of the mitochondrial outer membrane in live COS-7 cells using 3D-MP-SIM (left), showing a fission event at 11.78 s (white arrowheads) and a fusion event at 14.26 s (yellow arrowheads) (right). Scale bars, 5 μm (left) and 1 μm (right). (F) In the same region, an invagination occurred at 18.91 s, followed by completed fission at 19.22 s. White arrowheads indicate the nanotunnel tip and the fission site. Scale bar, 1 μm. Reproduction with permission [89]. Copyright 2025, Springer Nature.

In 2012, Fiolka et al. [83] advanced SIM technology to achieve a lateral resolution of 120 nm and an axial resolution of approximately 360 nm. They successfully visualized dynamic cellular events, such as actin rearrangements and mitochondrial morphology, in live HeLa cells and imaged rapid structures, such as neuronal growth cone filopodia, without motion artifacts. The dual-objective 3D-SIM achieved isotropic axial resolutions of approximately 100 nm, providing detailed structural insights. In 2015, Li et al. [84] demonstrated high-numerical-aperture total internal reflection fluorescence (TIRF) SIM combined with a patterned activation nonlinear SIM. This method revealed cortical actin interactions, cytoskeletal remodeling, mitochondrial fusion/fission, and Golgi-mediated vesicle transport at sub-100-nm resolution (Fig. 8A), surpassing the traditional SIM capabilities. In 2021, Lin et al. [85] used adaptive optics with 3D-SIM to enhance the imaging of deeper cellular structures such as the ER, axons, and adhesion junctions. This method improved the lateral and axial resolutions to 150 and 570 nm, respectively, which are significantly better than those of conventional WF microscopy (280 nm lateral and 930 nm axial). In 2023, Chang et al. [86] introduced “projective oblique plane SIM” (POPSIM), which integrates SIM and oblique illumination. Unlike traditional 2D-SIM, POPSIM eliminates mechanical scanning, achieves double the spatial resolution and rapid imaging speeds (2.7 Hz for full-cell imaging), and successfully visualizes mitochondrial and ER dynamics (Fig. 8B). In 2023, Mo et al. [87] developed a background-filtering algorithm to enhance the SIM resolution to below 70 nm, providing clear imaging and quantification of actin filament dynamics in living cells (Fig. 8C). In 2024, Temma et al. [88] applied selective plane activation SIM (SPA-SIM) to thick sample. While single-photon activation offered rapid 2D imaging (1 frame/s [fps]), 2-photon activation significantly improved the 3D spatial resolution, imaging 100- to 200-μm cell spheroids with reduced motion artifacts, thus validating the capability of SPA-SIM for thick-sample mitochondrial imaging (Fig. 8D). In 2025, Chen et al. [89] reported a novel 3-beam multiplane 3D-MP-SIM optimized for studying mitochondrial dynamics. This approach reduced the acquisition time to less than 1 s, attaining lateral and axial resolutions of approximately 120 and 300 nm, respectively. High-speed imaging of COS-7 mitochondrial dynamics (double-invagination fission, nanotunnel splitting, and vesicle fusion) demonstrated its exceptional ability to perform real-time ultrastructural studies (Fig. 8E and F).

Despite its significant advancements, SR-SIM has several critical limitations. Both methods rely heavily on computational image reconstruction, which can introduce artifacts and inaccuracies, particularly in highly dynamic or optically dense biological samples. In addition, their relatively slower acquisition speeds compared with traditional microscopy can restrict the effective imaging of rapid cellular events, potentially missing crucial transient dynamics. Furthermore, the prolonged illumination necessary to acquire multiple structured illumination frames can lead to increased photobleaching and phototoxicity, thereby limiting long-term live-cell imaging applications. Therefore, future studies should focus on developing faster acquisition protocols, improving computational algorithms for artifact reduction, and optimizing illumination schemes to minimize the photodamage. Overcoming these challenges will significantly enhance the capabilities of the SR-SIM, making it an even more powerful tool for dynamic and long-term studies in cell biology and biomedicine [90].

### Deep-learning-based SIM for live-cell imaging

Deep-learning-based SIM (DL-SIM) integrates advanced machine learning algorithms, particularly CNNs, with traditional SIM reconstruction to overcome limitations in resolution, speed, and phototoxicity [91,92]. By learning the nonlinear mapping between low-contrast raw images and high-fidelity reconstructions, DL-SIM enables SR imaging with fewer input frames, thereby accelerating image acquisition and reducing light exposure. This approach enhances spatial resolution and contrast while effectively denoising and suppressing reconstruction artifacts, allowing high-quality imaging under low-illumination conditions [93].

**Fig. 9.**
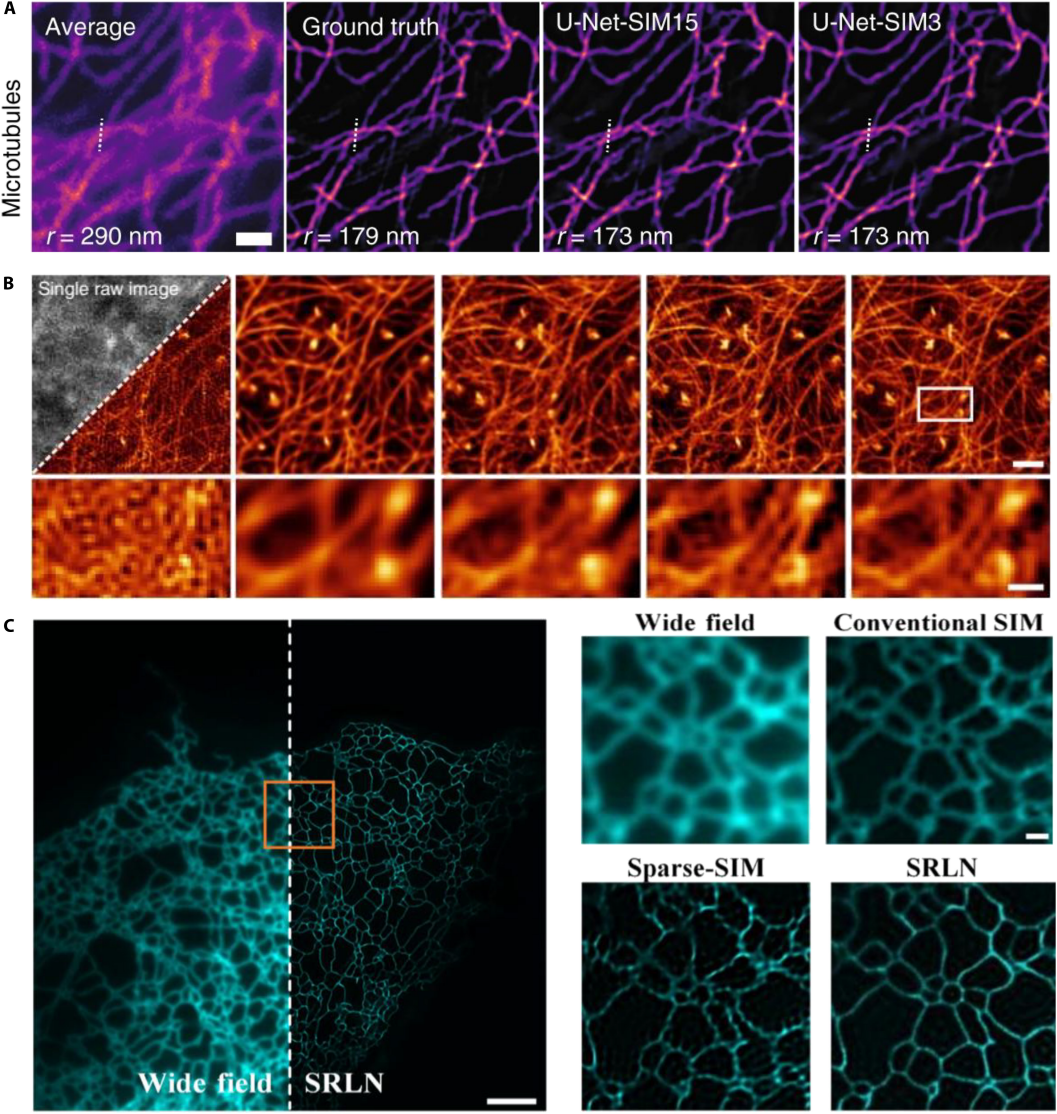
Deep learning SIM imaging. (A) Mitochondria and F-actin SIM raw data images as inputs, training scU-Net on live-cell images. Scale bar, 1 μm. Reproduced with permission [94]. Copyright 2020, Springer Nature. (B) Comparison of rationalized deep learning SIM with other advanced SIM methods for actin imaging. Scale bars, 1 μm. Reproduced with permission [95]. Copyright 2023, Springer Nature. (C) WF and SRLN images of the ER reconstruction. Left: WF and SRLN images. Scale bar, 3 μm. Right: Magnified view of the area shown in the left panel for WF, conventional SIM, sparse SIM, and SRLN images. Scale bar, 0.5 μm. Reproduced with permission [97]. Copyright 2024, Elsevier.

In 2020, Jin et al. [94] applied U-Net CNN architectures for SIM image reconstruction, achieving fast and high-quality imaging even under low-light conditions. Compared to GANs, U-Net provides simpler and more stable training with only 50 to 70 training samples and approximately 2,000 training cycles. They further enhanced the method by developing an scU-Net that combines 2 U-Nets through skip connections. These models enable clear imaging of cellular structures, such as microtubules, mitochondria, adhesion sites, and actin filaments, at low illumination levels (Fig. 9A), facilitating the rapid visualization of live-cell dynamics while minimizing phototoxicity and photobleaching. In 2022, Qiao et al. [95] introduced a rationalized deep learning SR approach that significantly enhanced the real-time imaging of rapid subcellular processes. Their method overcame the common artifact limitations of conventional reconstruction algorithms, providing over 10-fold improvement in the SR information (Fig. 9B). These advances represent substantial progress in SIM imaging and yield more precise and dynamic tools for biological and medical research. In 2023, Wang et al. [96] proposed a total depth variance SIM (TDV-SIM), a hybrid reconstruction approach that combines physical inversion models and depth-variance regularization. This method addresses the limitations of purely physical or deep-learning-based approaches, mitigating potential image degradation or hallucinations caused by deep learning alone. TDV-SIM effectively captured high-resolution images of actin filaments, the ER, and mitochondrial cristae. In 2024, Song et al. [97] developed a scale Richardson–Lucy network (SRLN), integrating Richardson–Lucy deconvolution with neural network architectures to reconstruct SIM images. After extensive training, the SRLN provided a spatial resolution of ~70 nm from raw SIM images across diverse imaging setups, successfully reconstructing high-quality images of the cellular ER (Fig. 9C). This demonstrates SRLN’s robustness of SRLNs and their general applicability in live-cell SIM imaging.

Recent developments have further improved the reconstruction accuracy by combining physical modeling with data-driven learning. Hybrid frameworks, such as TDV-SIM and SRLN, incorporate optical priors, including point-spread functions and illumination patterns, into deep learning architectures, mitigating image degradation and artifacts while preserving fine structural details [97,98]. Moreover, integrating multilevel wavelet transforms with deep learning has expanded SIM’s applicability of SIMs to complex biological environments by improving feature extraction and image restoration under photon-limited conditions. Collectively, these advances establish DL-SIM as a powerful, low-phototoxicity platform for the rapid, high-fidelity visualization of dynamic cellular processes [99].

In the future, deep learning applications in SIM will increasingly focus on real-time adaptive imaging and quantitative analysis. Reinforcement learning and Bayesian optimization techniques enable the automatic adjustment of excitation and depletion intensities, allowing real-time imaging with minimal light dose, thereby improving cellular viability and imaging precision. Furthermore, the combination of deep learning with physical models has led to significant advancements in denoising, image reconstruction, and temporal consistency, thereby facilitating long-term dynamic imaging. Standardized datasets and cross-platform validation will further enhance the reliability and reproducibility of these algorithms, thereby promoting the application and transfer of technologies across different experimental platforms. In addition, as multicolor imaging technologies advance, deep-learning-based SIM will enable the realization of more color channels with low phototoxicity, thereby expanding the potential of deep-tissue imaging applications. Ultimately, the integration of deep learning with SIM will shift toward quantitative biological analysis, extracting interpretable nanoscale parameters such as organelle contact frequency, membrane remodeling, and protein clustering dynamics, providing precise molecular-level analytical tools for disease research and clinical applications [100].

### Applications of SIM imaging technology for dynamic live-cell behavior

SIM microscopy is highly effective for studying live-cell dynamics owing to its superior spatial resolution and minimal phototoxicity. Traditional optical microscopy suffers from a limited resolution and insufficient temporal capability to accurately capture rapid cellular processes [101]. SIM generates thin optical sections with enhanced image quality, enabling real-time monitoring of intracellular structures and their dynamics [102].

**Fig. 10.**
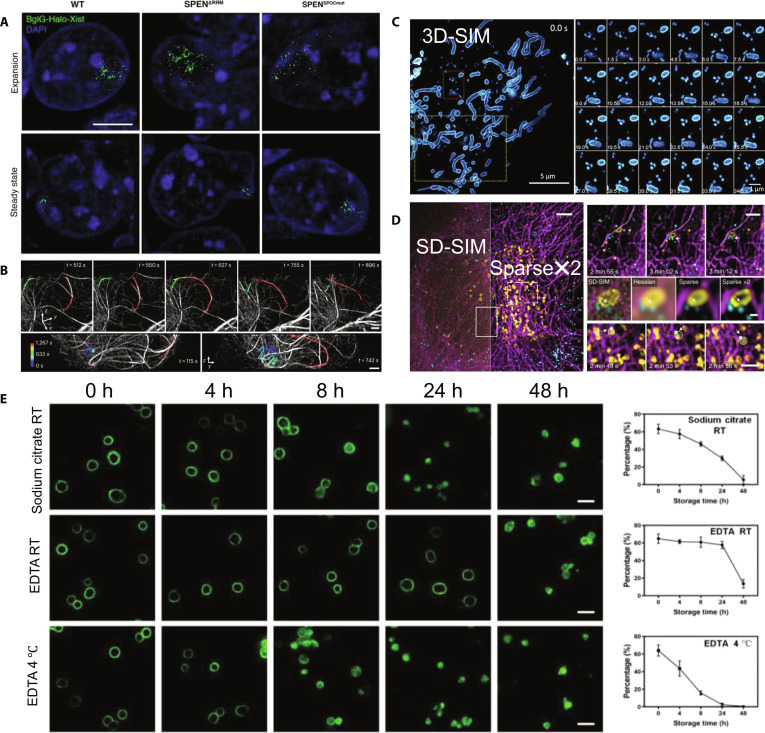
Applications of SIM in studying cellular behavior. (A) Representative 3D-SIM images showing the localization of Xist molecules in mouse embryonic stem cells during the expansion and steady-state phases. Scale bar, 1 μm. Reproduction with permission [105]. Copyright 2021, American Association for the Advancement of Science. (B) Live immune cell microtubule dynamics. The images show MTOC (cyan spheres), overlapping microtubules (red, yellow, and green), and curved microtubules (red and yellow spheres). Scale bars, 1 μm. Reproduction with permission [107]. Copyright 2023, Springer Nature. (C) Fast 3D-SIM time-lapse imaging of MDVs and dynamic mitochondrial tubules in H9c2 cells cultured in galactose medium. Reproduction with permission [109]. Copyright 2022, Wiley-VCH GmbH. (D) Sparse-SIM images of lysosome and peroxisome movement along microtubules. Left: Snapshot of HeLa cells labeled with microtubule protein–eGFP (magenta), Pex11a–blue FP (cyan), and Lamp1–mCherry (yellow). Right: An enlarged view of the region indicated in the left panel. Scale bars, 5 μm (left) and 3 μm (right). Reproduction with permission [110]. Copyright 2022, Springer Nature. (E) Platelet SIM images across whole-blood storage variables (anticoagulant: sodium citrate/EDTA; temperature: room temperature [RT]/4 °C; time: 0, 4, 8, 24, and 48 h) and corresponding percentages of ring-shaped marginal band microtubule structures. Reproduction with permission [111]. Copyright 2022, Wiley-VCH GmbH.

High temporal resolution is essential for examining dynamic cellular processes. SIM captures rapid cellular events, including cell division, membrane dynamics, cytoskeletal remodeling, and intracellular trafficking. For example, SIM visualizes spindle microtubule rearrangements during cell division and precisely tracks chromosomal movements [103,104]. In 2021, Rodermund et al. [105] developed RNA-SPLIT combined with 3D-SIM, enabling the time-resolved visualization of Xist RNA dynamics during X chromosome inactivation. Their method successfully revealed the detailed localization and dynamic movement of individual Xist RNA molecules in mouse embryonic stem cells (Fig. 10A).

Unlike SR techniques such as STED or photoactivated localization microscopy (PALM), which often require longer acquisition times and increase phototoxicity, SIM’s lower illumination intensity allows for rapid, long-term imaging with minimal photodamage. For example, SIM effectively captures fast synaptic events in neurons, thereby elucidating the detailed mechanisms of neuronal activity and synaptic plasticity [106]. In 2023, Li et al. [107] introduced a deep-learning-enhanced SIM method that achieved isotropic ~120-nm resolution with integrated denoising, significantly improving the volumetric imaging quality across multiple time points (Fig. 10B).

SIM is also valuable for cell migration studies, enabling real-time visualization of dynamic changes in microtubules and actin filaments. In addition, SIM has been used to investigate intracellular membrane systems such as the ER, Golgi apparatus, and vesicular transport [108], providing detailed insights into intracellular trafficking mechanisms. Mitochondria-derived vesicles (MDVs), which are critical for mitochondrial health, are challenging to image because of their rapid dynamics and small sizes. In 2021, Opstad et al. [109] leveraged 3D-SIM to capture the high-resolution dynamics of MDVs and mitochondrial tubules (Fig. 10C), demonstrating SIM’s capability of SIM for visualizing dynamic organelle behavior.

SIM’s multicolor imaging capability of SIM, combined with its high temporal resolution, facilitates the simultaneous tracking of multiple cellular components. In 2022, Zhao et al. [110] introduced Sparse-SIM, a deconvolution algorithm that utilizes biological structure sparsity and continuity priors, which effectively doubled SIM’s resolution of SIM to approximately 60 nm at imaging rates of up to 60 Hz. Sparse-SIM successfully resolved complex intracellular structures, such as lysosomes and peroxisomes (Fig. 10D), considerably enhancing dynamic live-cell imaging.

SIM translates nanoscale morphology into quantifiable clinical readouts. In renal pathology, SIM can replace or complement EM by super-resolving podocyte foot processes and, with automated analysis, quantifying foot-process width and slit-diaphragm density, enabling an objective assessment of effacement. In liquid biopsy, Xu et al. [111] established a standardized sample workflow and an automated high-throughput pipeline to analyze 280,000 SIM images from 206 donors, extracting nanoscale distribution signatures of platelet α-granules that discriminate multiple cancers and support diagnostic stratification and therapy monitoring (Fig. 10E). In summary, SIM microscopy, with its unique combination of high spatial and temporal resolutions, has immense potential for investigating dynamic cellular processes. Its applications in cell division, signal transduction, membrane dynamics, organelle movement, and immune responses have significantly deepened our understanding of complex cellular behavior. As imaging techniques and computational algorithms evolve, SIM will play an increasingly pivotal role in elucidating the dynamic cellular mechanisms underlying various biological processes and diseases.

## SMLM for Single-Molecule Imaging in Living Cells

**Fig. 11.**
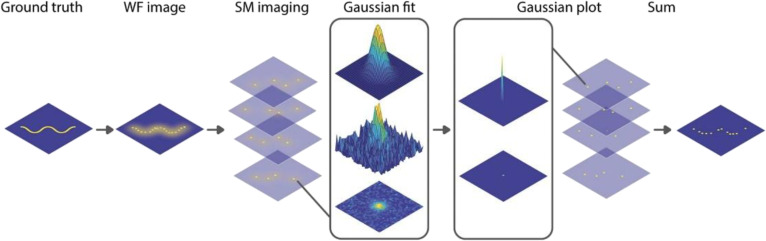
Principle of SMLM. Reproduction with permission [112]. Copyright 2018, IOP Publishing Ltd.

SMLM encompasses a family of SR techniques based on the precise localization of individual fluorescent molecules and the reconstruction of images with nanometer-level resolution (Fig. 11) [112]. By temporally separating fluorescence emissions from neighboring molecules and accurately localizing each emission, SMLM surpasses traditional diffraction-limited microscopy. Common SMLM techniques include stochastic optical reconstruction microscopy (STORM), PALM, point accumulation for imaging in nanoscale topography (PAINT), and DNA-PAINT [113]. These powerful approaches enable detailed visualization of intracellular architecture and dynamic molecular processes, providing unprecedented insights into cellular function and complex biological mechanisms.

### STORM for imaging in living cells

**Fig. 12.**
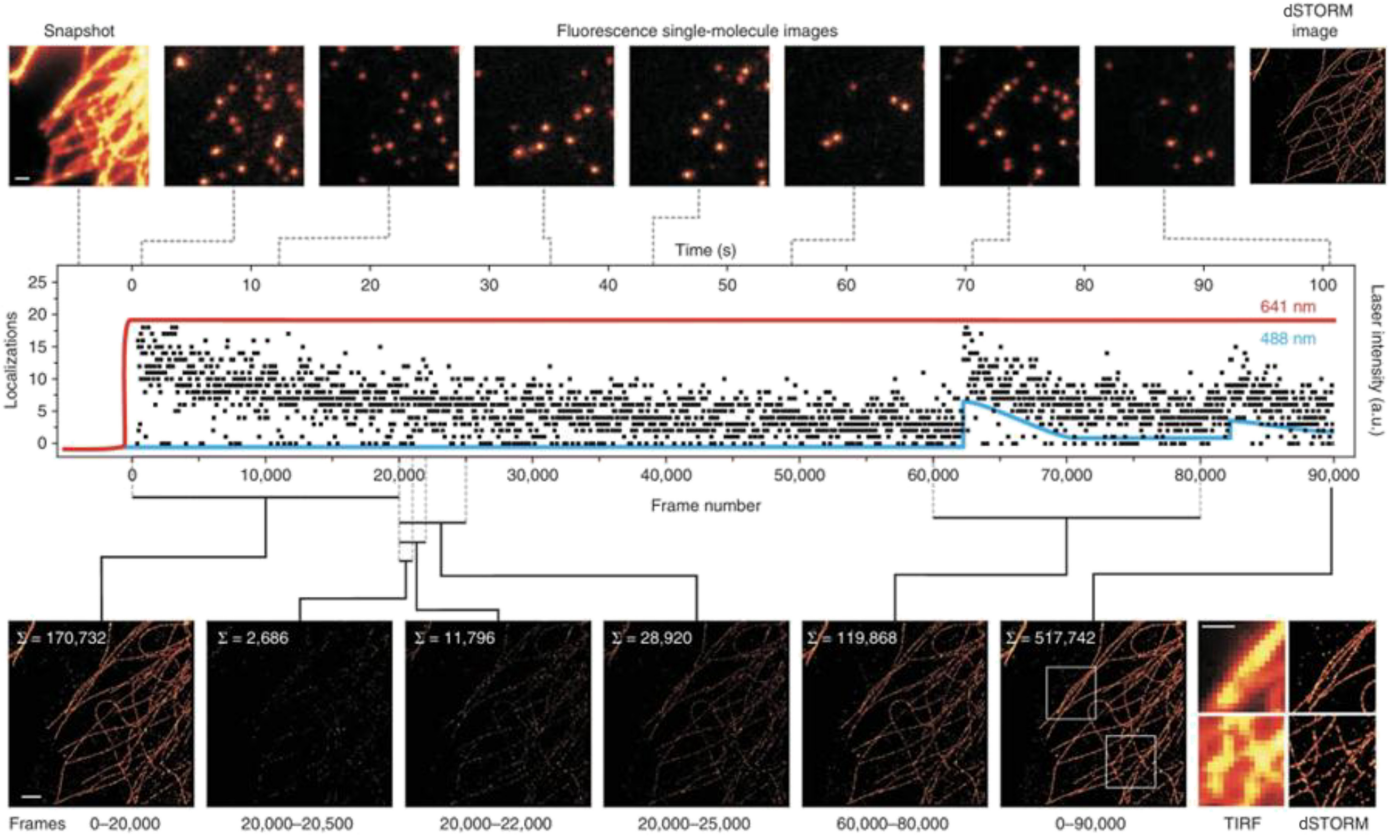
Principle of STORM. Reproduction with permission [193]. Copyright 2011, Springer Nature.

The principle of STORM is based on the random excitation and de-excitation of fluorescent probes. A widely used variant, direct STORM (dSTORM), achieves the same stochastic blinking as conventional organic dyes (e.g., Alexa Fluor 647 or Cy5) by driving them into a long-lived dark state with a thiol-rich reducing buffer and allowing them to return stochastically to the fluorescent state, eliminating the need for an activator reporter pair. Figure 12 summarizes the dSTORM workflow: Individual emitters are switched on, localized with nanometer precision, and iteratively combined to yield a super-resolved reconstruction. By recording the temporal information of these events, STORM achieves a higher-resolution than traditional microscopy techniques at the subcellular level [114]. The application of STORM spans various aspects at the cellular and molecular levels, from the distribution of organelles to the localization of proteins, and provides detailed insights into cellular structures and dynamics [115].

**Fig. 13.**
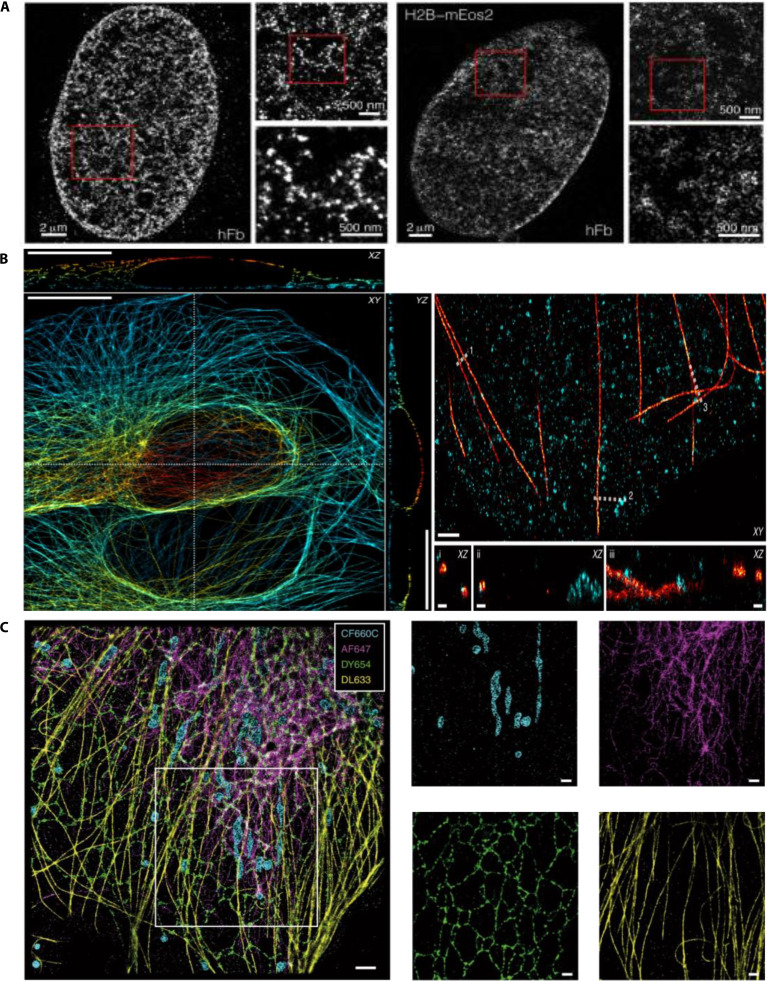
STORM live-cell imaging. (A) Representative STORM images of human fibroblast (hFb) nuclei (left) and live cells expressing H2B-mEos2 (right). The progressively zoomed-in regions within the red square next to each cell nucleus. Reproduction with permission [116]. Copyright 2015, Cell. (B) Full-cell 3D reconstruction of microtubules (left) and 2-color 3D imaging of microtubules stained with Alexa Fluor 647 (AF647; red) and clathrin (right). Scale bars, 10 μm (left) and 1 μm (right). Reproduction with permission [118]. Copyright 2020, Springer Nature. (C) Tetra-color SR reconstructed image of mitochondria outer membrane (cyan), intermediate filaments (magenta), ER (green), and microtubules (yellow) in COS-7 cells based on ExR-STORM. Scale bars, 2 μm (left) and 1 μm (right). Reproduction with permission [119]. Copyright 2023, Springer Nature.

In 2015, Ricci et al. [116] utilized STORM to image chromatin organization in human fibroblast nuclei by mapping the distribution of histone H2B. They observed that discrete nanodomains of H2B were enriched at the nuclear periphery, highlighting STORM’s capacity of STORM to resolve intricate nuclear structures (Fig. 13A). In 2018, Bintu et al. [117] combined STORM with diffraction-limited imaging to construct high-resolution 3D images of chromatin fragments. They identified chromatin topological domains (Topologically Associating Domain-like domains), demonstrating STORM’s suitability of STORM for detailed structural analysis of chromatin. In 2020, Diekmann et al. [118] investigated the effects of excitation intensity on SMLM image quality, particularly for high-speed imaging. By systematically evaluating the fluorophores, labeling methods, and imaging buffers, the 3D-dSTORM protocols were optimized, enabling high-throughput, multicolor 3D imaging. Their improved approach effectively visualized the entire cellular microtubule network and clathrin-coated structures with high resolution and efficiency (Fig. 13B). Multicolor imaging is crucial for examining molecular colocalization and interactions. In 2023, Wu et al. [119] introduced excitation-resolved STORM (ExR-STORM), which distinguishes 4 spectrally overlapping far-red dyes using only 3 excitation lasers. ExR-STORM achieved sub-3% spectral cross-talk and precise multicolor 3D visualization of cellular structures, such as mitochondria, intermediate filaments, ER, and microtubules (Fig. 13C).

Despite significant advancements in resolution and versatility, STORM continues to face several challenges. However, its reliance on high-intensity illumination and specialized imaging buffers can lead to considerable photobleaching and phototoxicity, restricting long-term live-cell studies. In addition, the requirement for extensive computational postprocessing and the risk of localization errors can introduce artifacts that complicate the interpretation and analysis of data. Furthermore, the imaging speed remains relatively slow, limiting its ability to capture highly dynamic cellular events in real time. Future improvements in fluorophore stability, buffer formulations, computational algorithms, and imaging acquisition speeds will further enhance STORM’s robustness of STORM and expand its applicability in dynamic biological investigations.

### PALM for imaging in living cells

PALM is an advanced SR microscopy method that facilitates nanoscale imaging using photoswitchable FPs [120]. In PALM, FPs are activated, emit fluorescence, and subsequently switch off, ensuring only a subset of fluorescence. Precise localization of these individual events enables the reconstruction of high-resolution images, revealing intricate cellular and subcellular structures [121].

**Fig. 14.**
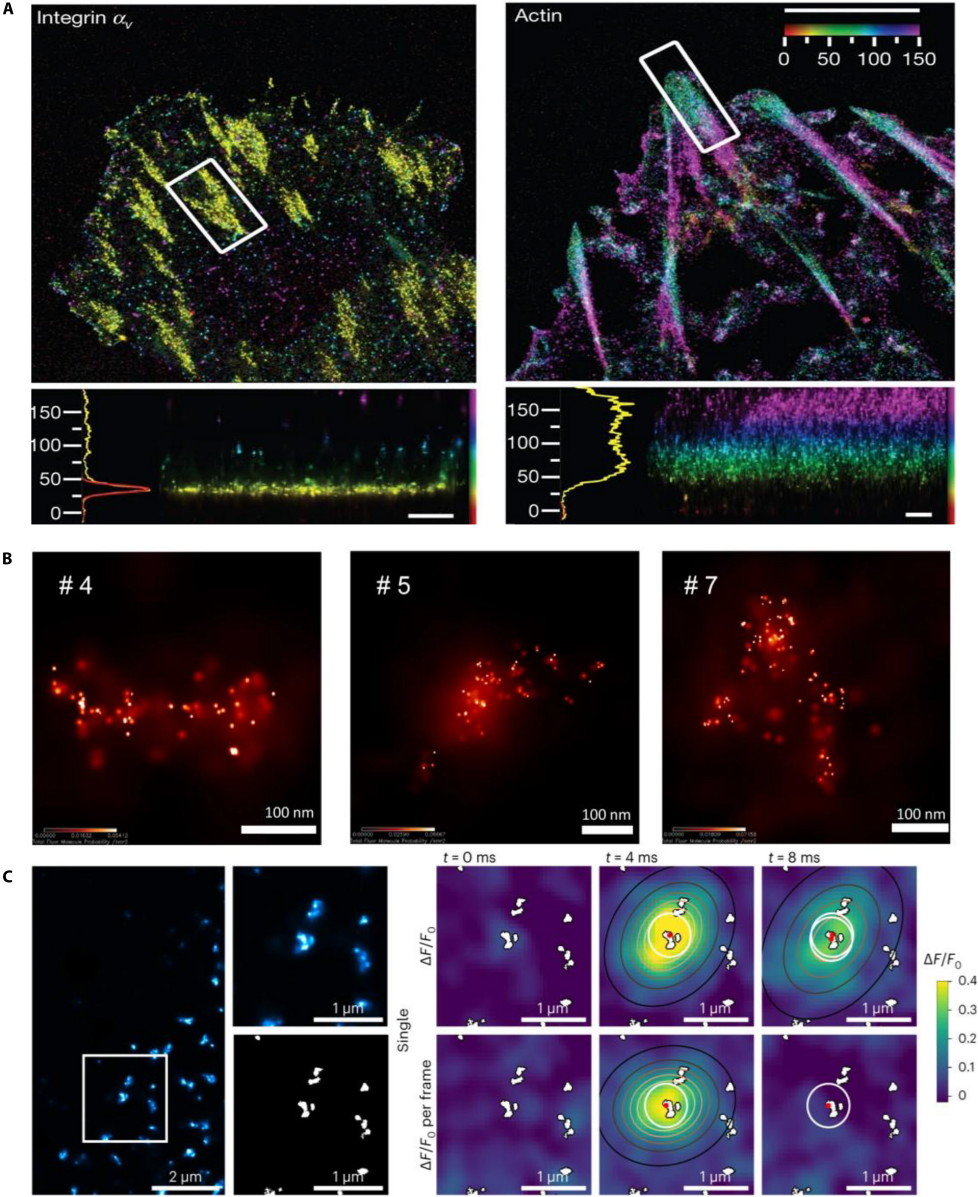
PALM cellular imaging. (A) iPALM imaging of membrane marker integrins and actin. Scale bars, 500 nm. Reproduction with permission [122]. Copyright 2010, Springer Nature. (B) Representative images of target regions from 6 different cells obtained by PALM. Reproduction with permission [124]. Copyright 2022, Springer Nature. (C) SR PALM imaging of RyRs on the cell surface and associated Ca^2+^ imaging. Reproduction with permission [125]. Copyright 2022, Springer Nature.

In 2010, Kanchanawong et al. [122] used interferometric PALM (iPALM) to elucidate the nanoscale organization of focal adhesions in cell–extracellular matrix interactions. They visualized a vertically stratified structure comprising integrin signaling, force transmission, and actin regulatory layers. Integrin tails were localized approximately 20 nm from the membrane surface, providing molecular details crucial for understanding the mechanics of adhesion (Fig. 14A). Despite PALM’s precision of PALM, multiple blinking events can cause artificial clustering artifacts. In 2022, Jensen et al. [123] introduced a model-based correction method to address this issue and successfully demonstrated precise aggregation patterns of connector adapter proteins at the immune synapses. In the same year, Parteka-Tojek et al. [124] used iPALM to image chromatin loops in human lymphoblasts, achieving a remarkable localization accuracy of 2 to 22 nm and providing detailed insights into the 3D structure of chromatin and its influence on gene expression (Fig. 14B). In 2023, Hou et al. [125] applied PALM to cardiac myocytes to explore the correlation between calcium sparks and the nanoscale arrangements of ryanodine receptors (RyRs). High-resolution PALM imaging linked RyR distribution directly to calcium signaling dynamics, offering crucial insights into cardiac cellular functions (Fig. 14C).

Despite its remarkable resolution and precision in visualizing subcellular structures, PALM has several limitations. A primary challenge is their dependence on specialized photoswitchable FPs, which can exhibit limited brightness, photostability, and switching efficiency, potentially compromising the image quality and limiting prolonged or repeated imaging sessions. In addition, multiple blinking events in FPs can introduce localization errors and artificial clustering artifacts, complicating accurate data interpretation. PALM’s relatively slow acquisition speed also restricts its ability to effectively monitor rapid cellular dynamics. Future developments should focus on engineering brighter and more photostable FPs, improving computational algorithms to minimize reconstruction artifacts, and increasing the imaging speed. Addressing these challenges will enhance PALM’s reliability of PALM and expand its application in dynamic live-cell imaging.

### PAINT and DNA-PAINT for imaging in living cells

PAINT uses dynamic and transient molecular binding and unbinding for high-resolution imaging. DNA-PAINT uses complementary DNA sequences to enable precise target localization [126]. Both methods circumvent the need for photoswitchable fluorophores, making them particularly suitable for live-cell imaging.

**Fig. 15.**
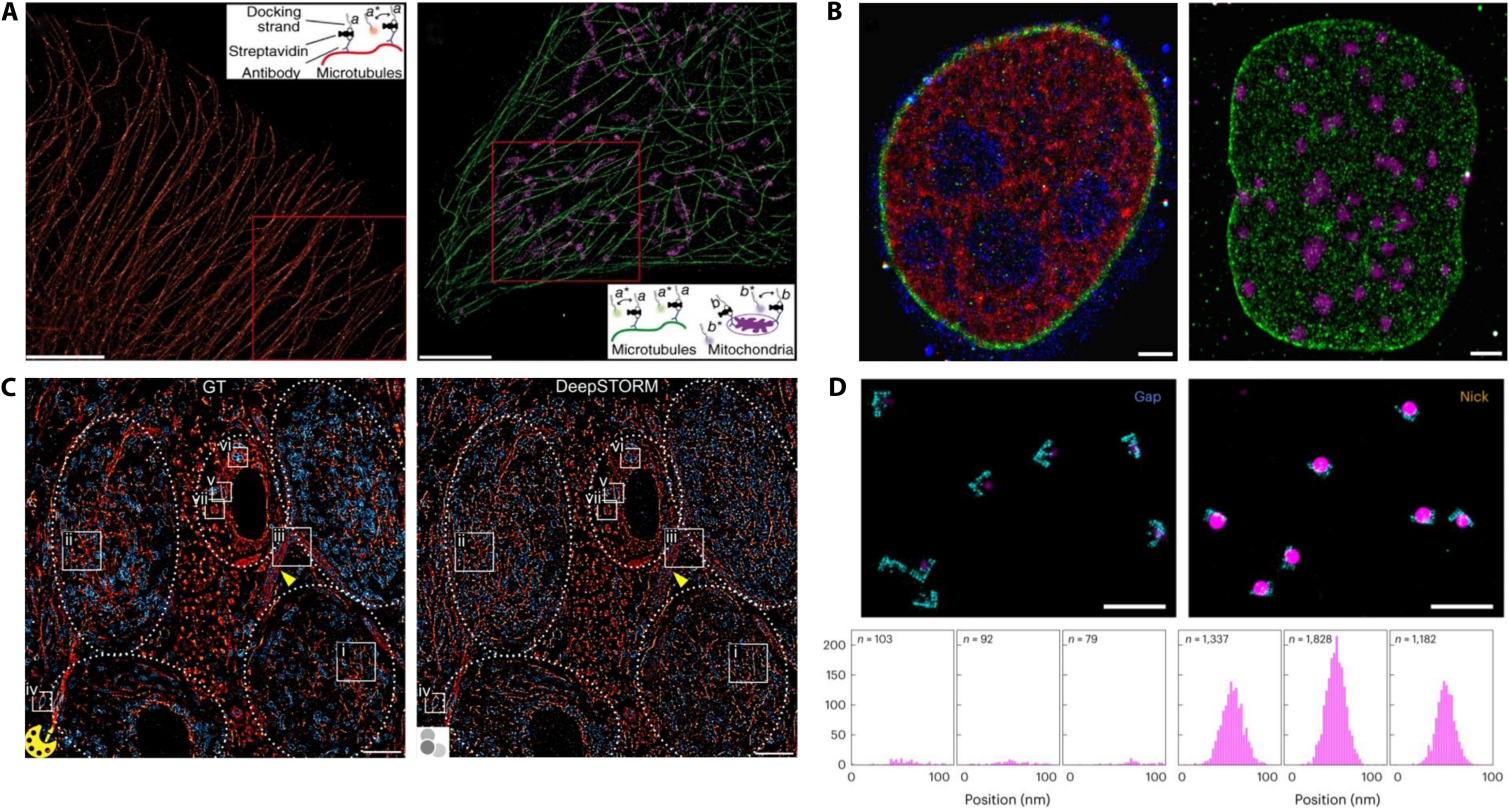
PAINT and DNA-PAINT live-cell imaging. (A) DNA-PAINT SR imaging of microtubules in HeLa cells. Scale bars, 5 μm. Reproduction with permission [127]. Copyright 2014, Springer Nature. (B) Three-color immunostaining DNA-PAINT imaging of HeLa cells. Scale bars, 2 μm. Reproduction with permission [128]. Copyright 2017, Springer Nature. (C) Imaging of tissue samples showing microtubule protein and Translocase of Outer Mitochondrial Membrane 20. Scale bars, 5 μm. Reproduction with permission [130]. Copyright 2022, Springer Nature. (D) DNA-PAINT data imaged with Atto647N and Cy3B imaging systems. Reproduction with permission [131]. Copyright 2023, Springer Nature.

In 2014, Jungmann et al. [127] applied DNA-PAINT and Exchange-PAINT for multicolor imaging, achieving sub-10-nm resolution for imaging microtubules and mitochondria in live HeLa cells (Fig. 15A). In 2017, Schueder et al. [128] extended DNA-PAINT with confocal microscopy, enabling 3D imaging of thick samples (~10 μm in depth) at resolutions of ~20 nm (lateral) and ~80 nm (axial), successfully visualizing nuclear structures and histones in HeLa cells (Fig. 15B). In 2020, Brockman et al. [129] introduced Tension-PAINT, which combines molecular tension sensors with DNA-PAINT to visualize integrin-mediated forces at a resolution of ~25 nm, revealing the mechanical dynamics at the leading edges of cells. In 2022, Narayanasamy et al. [130] developed a neural network (DeepSTORM) that accelerated DNA-PAINT imaging acquisition, enabling rapid high-density emitter imaging and multicolor SR of neuronal tissues (Fig. 15C). In 2023, Banerjee et al. [131] quantified base-stacking interactions in DNA structures using DNA-PAINT and custom-designed DNA nanostructures. Their multiplexed approach accurately measured the single-molecule free energies of base-stacking interactions, significantly enhancing the stability of nanostructures (Fig. 15D).

In recent years, substantial progress has been made in DNA-PAINT multiplexing, pushing this technique toward higher-throughput and quantitative nanoscopy. Steen et al. [132] provided a systematic performance analysis of DNA-PAINT systems, defined the practical limits of resolution, binding kinetics, and probe orthogonality, and established design guidelines for multicolor, high-speed imaging. Building on this framework, Lycas and Manley [133] developed an adaptor-mediated DNA-PAINT approach that uses modular DNA adaptors to simplify probe exchange and enable efficient multitarget imaging with minimal cross-talk. More recently, Piantanida et al. [134] summarized emerging strategies that couple optimized probe chemistry, rapid hybridization kinetics, and drift-correction algorithms to achieve large-scale quantitative mapping of cellular nanostructures. Together, these developments have transformed DNA-PAINT into a robust and scalable multiplexed SR platform capable of simultaneously visualizing numerous molecular species with nanometer-scale precision.

Despite their high spatial resolution and versatility, PAINT and DNA-PAINT techniques still have several limitations. However, their reliance on transient binding kinetics often results in slower image acquisition speeds, posing challenges in capturing fast and dynamic cellular processes in vivo. In addition, these methods require careful optimization of the probe concentration and binding kinetics, thus increasing the experimental complexity and the risk of nonspecific interactions. DNA-PAINT requires stringent control over probe sequences and hybridization conditions, potentially complicating experimental design and data interpretation. Moreover, the extensive computational analysis required to localize single molecules and reconstruct images can introduce artifacts and inaccuracies. Future developments should prioritize improving probe-binding dynamics, accelerating acquisition rates through advanced computational approaches such as deep learning, and refining experimental protocols to reduce complexity and enhance reproducibility. Addressing these challenges will further expand the applications of PAINT and DNA-PAINT in live-cell imaging and nanoscale biological research.

To analyze the specific details of SMLM technologies, Table 2 outlines the characteristics, applications, advantages, and limitations of the different SMLM techniques.

**Table 2.** Comparison of advantages and limitations of SMLM imaging techniques

Technique	Applications	Advantages	Limitations	Reference
STORM	Cytoskeleton, membrane protein organization, subcellular structures within cells	High resolution, capable of revealing nanoscale cellular structures	High demands on fluorophores and long data acquisition times	[198]
PALM	Protein localization and distribution, intracellular molecular dynamics	Use of endogenously expressed FPs, suitable for live-cell imaging	Requires specific light-activated FPs; lower temporal resolution	[199]
PAINT	Membrane structures, cell–cell contact regions	No need for specific light-activated proteins; achieved with conventional fluorescence microscopes	Resolution limited by the density of blinking molecules; may require specific probes	[127]
DNA-PAINT	Molecular interactions, DNA and protein complexes	High resolution, down to a few nanometers; can be investigation of dynamic molecular interactions	Complex experimental design and optimization are complex; high data processing demands	[200]

### Emerging strategies for SR-SMLM: Deep-learning-based reconstruction and live-cell imaging enhancement

The essence of overcoming SMLM’s temporal limits of SMLM is to trade frames and doses for the algorithms. Deep-learning-based high-density reconstruction separates multiple emitters from the overlapping point spread function (PSF), permitting higher per-frame emitter densities and thus reliable localization in far fewer frames. In parallel, low-photon denoising and reconstruction preserve precision under ultralow-photon budgets, shorten exposure, and reduce photobleaching and phototoxicity [135,136].

Chen et al. [137] used single-frame SR microscopy (SFSRM), which leverages a subpixel edge map and multicomponent optimization to reconstruct SR images from a single diffraction-limited frame, thereby eliminating thousands of frames required by conventional SMLM. Under practical emitter densities and signal-to-noise ratio (SNR), SFSRM enables the imaging of mitochondria–ER interactions with 30-nm spatial and 10-ms temporal resolutions. Basumatary et al. [138] introduce an event-based SMLM strategy that exploited subblink photon timing to generate multiple PSF realizations per molecule. By aggregating these short-window events, the method yields robust centroid estimates and achieves ~10-nm localization precision while supporting long-term live-cell measurements of the molecular dynamics.

The future development of SMLM will emphasize intelligent acquisition, optimized photophysics, and quantitative analysis. The integration of deep learning with adaptive optics, Bayesian optimization, and reinforcement learning control will enable the real-time regulation of illumination intensity and activation density, balancing precision and speed under ultralow photon budgets. In addition, progress in far-red and NIR fluorophores with reversible photoswitching kinetics and enhanced photostability will extend SMLM to deep-tissue and multicolor imaging regimes.

Standardized open datasets and physics-informed reconstruction algorithms enhance reproducibility and enable reliable cross-platform benchmarking [139]. Furthermore, multimodal integration that combines SMLM with correlative techniques, such as Raman nanoscopy, STED, or cryo-electron tomography, transforms localization data into quantitative molecular maps, linking nanoscale architectures with biochemical functions in living systems. Collectively, the convergence of the optimized fluorophore design, AI-driven reconstruction, and intelligent acquisition is expected to redefine SMLM as a quantitative, high-speed, and low-dose nanoscopy framework for real-time molecular imaging in biological and clinical contexts [140].

### Applications of SMLM techniques in investigation on cellular behavior at the single-molecule level

SMLM has become crucial for studying cellular behavior at the nanoscale, offering spatial resolutions of 10 to 20 nm. SMLM provides unprecedented insights into protein localization, molecular interactions, and signaling pathways, crucial for understanding processes such as cytoskeletal remodeling, vesicle transport, mitochondrial dynamics, and cell adhesion [141,142].

**Fig. 16.**
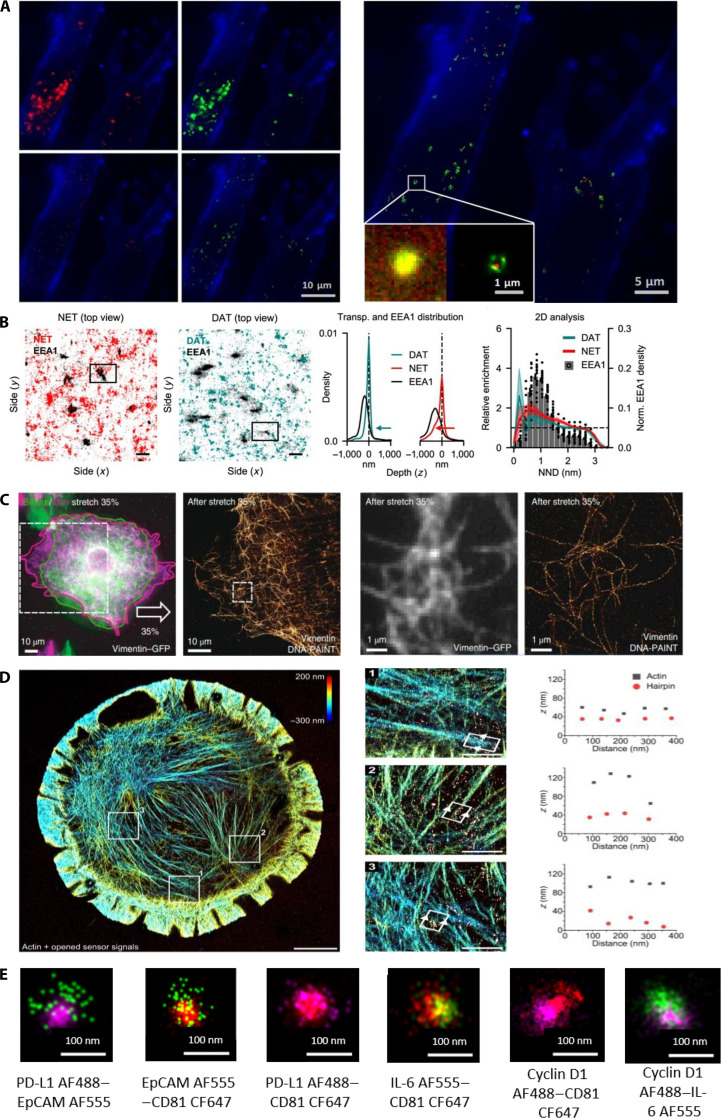
Applications of SMLM in studying live-cell behavior. (A) Colocalization of exosomes and lysosomes. Exosomes (red), membrane (blue), and lysosomes (green). Reproduction with permission [143]. Copyright 2016, American Chemical Society. (B) Representative top-view images of EEA1 and DAT/NET in cells, acquired using scattered light dSTORM. Scale bars, 0.5 μm. Reproduction with permission [145]. Copyright 2022, Springer Nature. (C) Low-resolution fluorescence and DNA-PAINT SR images of vimentin after cell stretching (left). Corresponding magnified view of the dashed box in the left panel (right). Reproduction with permission [147]. Copyright 2020, Springer Nature. (D) Dual-target 3D SR characterization of actin cytoskeleton and the hairpin sensor. Using Lifeact-Cy3B SR to show the actin network, overlaid with the extended sensor signal (red), and zoomed-in view of the highlighted region. Scale bars, 5 μm (left) and 1 μm (right). Reproduction with permission [148]. Copyright 2021, Springer Nature. (E) SR dSTORM imaging for extracellular vesicle characterization. IL-6, interleukin-6. Reproduction with permission [149]. Copyright 2025, MDPI.

Exosomes are small, protein-rich membrane vesicles secreted by cells. They play critical roles in intercellular communication, diagnostics, and drug delivery. To study exosomes in more detail, Chen et al. [143] used dual-color PALM/STORM imaging in 2016 to visualize the fine structures of 2 membrane receptors. They observed interactions between cancer-derived exosomes and normal cells, as well as the colocalization of exosomes with lysosomes in recipient cells (Fig. 16A). These findings lay the foundation for further research on the role of exosomes in cancer metastasis. In 2020, Yan et al. [144] investigated the cause of diffusion slowdown at ER–plasma membrane contact sites. Using single-molecule dynamic microscopy with SR-SMLM and a solvent-sensitive probe (Nile Red), they tracked lipid accumulation and found that the diffusion slowdown was due to local protein crowding and not due to changes in the lipid sequence.

Protein transport is a fundamental cellular process that often requires 3D imaging for detailed analysis. In 2022, Ejdrup et al. [145] used dual-color SMLM to study the endocytosis of dopamine transporters (DATs) and norepinephrine transporters (NETs). Although these proteins are functionally similar, they exhibit distinct subcellular localizations and transport pathways. Using dSTORM combined with antibody labeling for EEA1, the authors obtained 3D SR images (Fig. 16B), providing new insights into the protein distribution and dynamics during transport.

SMLM is also widely used in cytoskeletal research, allowing for high-resolution imaging of microtubules, microfilaments, and intermediate filaments. This enabled a detailed study of cell shape changes and movements. In addition, SMLM has been applied to nuclear structure and gene expression studies by precisely localizing chromatin-labeling molecules [146]. This approach reveals the 3D organization and dynamic changes in chromatin, deepening our understanding of gene regulation. For example, Massou et al. [147] used DNA-PAINT in 2020 to image the stretching of vimentin and microtubules (Fig. 16C), capturing protein reorganization and force responses in mechanosensitive structures. In 2021, Schlichthaerle et al. [148] focused on cellular extracellular matrix sensing and the formation of focal adhesions. Using 3D DNA-PAINT, they demonstrated how extracellular ligand binding and mechanical forces were transduced into nanoscale protein structures within the actin cytoskeleton (Fig. 16D). Their work provides new insights into the study of ligand–receptor binding and force transmission at the molecular level.

SMLM has been applied to tissue diagnosis of glomerular diseases, liquid biopsy analyses of platelets and extracellular vesicles, and spatial mapping of tumor-associated receptors and protein complexes. These studies demonstrate that SMLM can serve not only as a high-throughput alternative or complement to EM but also as a quantitative tool for identifying disease-specific nanoscale spatial fingerprints. Raciti et al. [149] showed that dSTORM profiling of plasma extracellular vesicle protein nanosignatures provides a minimally invasive means of predicting and monitoring colorectal cancer recurrence, surpassing the capabilities of conventional tissue biopsy (Fig. 16E). Antunes-Ferreira et al. [150] used dSTORM to resolve nanoscale protein redistribution patterns within circulating platelets exposed to tumor-derived factors or cancer cells, enabling the visualization of P-selectin, CD63, and other granule-associated markers at an exceptional resolution of approximately 20 nm.

SMLM is equally valuable for studying mitochondrial dynamics, including fusion, fission, and protein distribution in the mitochondrial membranes. These high-resolution imaging results support the development of novel therapies, particularly for mitochondrial diseases. In 2024, Zheng et al. [151] used SMLM and other SR methods to image the mitochondrial inner membrane in HeLa cells, revealing tubular cristae structures and distances between adjacent cristae of 68 to 184 nm. Saguy et al. [152] developed a deep-learning-based approach called DBlink to improve both the temporal and spatial resolutions in SMLM. DBlink uses CNNs and long short-term memory models to capture frame-to-frame dependencies, enabling super-resolved videos of mitochondrial dynamics at spatial resolutions of 20 fps and 75 nm, respectively. This advancement allows for detailed observation of mitochondrial fusion, fission, and movement in live cells.

SMLM significantly enhances nanoscale imaging, providing crucial insights into protein localization, molecular interactions, and dynamic cellular processes. By providing spatial resolutions of 10 to 20 nm, SMLM enables detailed investigations of cytoskeletal remodeling, vesicle transport, mitochondrial dynamics, and cell adhesion. Advances in dual-color and 3D SMLM imaging have facilitated breakthroughs in our understanding of complex biological phenomena, such as exosome-mediated cellular communication, lipid dynamics at membrane contact sites, and distinct intracellular transport pathways. Despite these advancements, several challenges remain, including limited temporal resolution, potential localization inaccuracies, and phototoxicity concerns. The future development of improved fluorophores, optimized imaging protocols, and advanced computational algorithms, such as deep-learning-based approaches, will further enhance SMLM’s capacity of SMLM to capture rapid cellular dynamics and molecular behaviors, providing deeper insights into cellular function and disease mechanisms.

## Raman Spectrum as a Tool for Live-Cell Monitoring

Raman spectroscopy is a unique and valuable tool in cell biology because it is noninvasive and label-free. By detecting subtle shifts in molecular vibrations, Raman spectroscopy robustly reveals the cellular chemical composition and ultrastructure. Various Raman-based methods, such as surface-enhanced Raman scattering (SERS), stimulated Raman scattering (SRS), coherent anti-Stokes Raman scattering (CARS), and conventional Raman spectroscopy, are essential for studying cell metabolism, drug distribution, molecular interactions, and disease diagnostics [153]. This article explores the principles, features, applications, and challenges of Raman technologies to summarize high-precision molecular imaging and cellular-level analyses.

### SRS for live-cell imaging

SRS microscopy is an advanced imaging method that uses pump and probe lasers to excite molecular vibrations, enabling direct visualization of chemical components within live cells. On the basis of the energy transfer between lasers, SRS provides label-free, high-resolution images that are ideal for noninvasive, real-time imaging of biological samples [154].

**Fig. 17.**
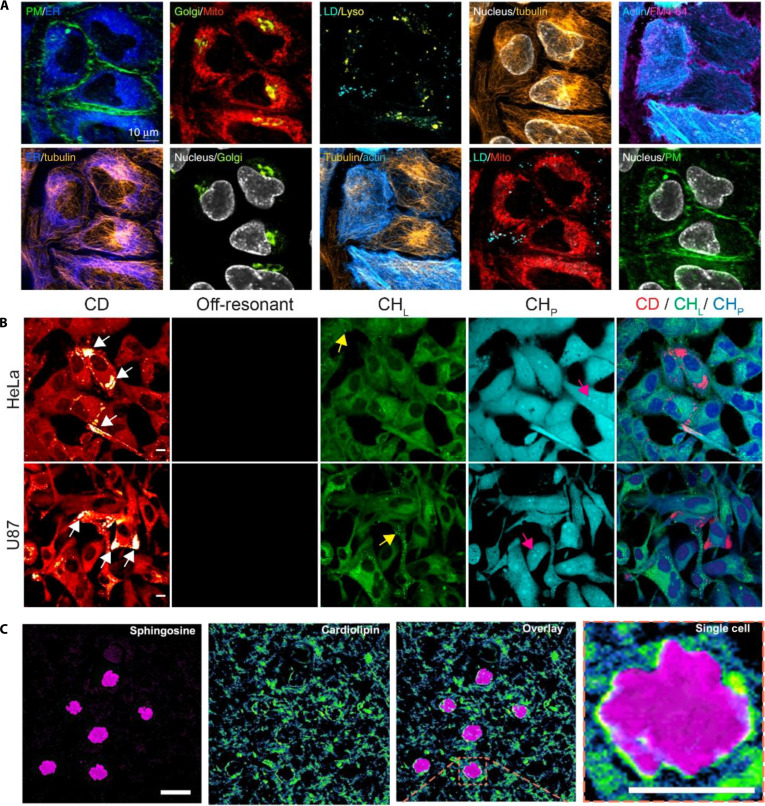
SRS live-cell imaging. (A) Optical imaging of live HeLa cells using 10 fluorescent probes. Each image shows the overlay of 2 species. PM, plasma membrane; Mito, mitochondria; LD, lipid droplets; FM4-64, GM 4-64 (styryl membrane dye, *N*-(3-triethylammoniumpropyl)-4-{6-[4-(diethylamino)phenyl]hexatrienyl}pyridinium dibromide) Reproduction with permission [155]. Copyright 2018, Springer Nature. (B) Imaging results of HeLa and U87 cells incubated in 7-glucose medium for 3 d. The CD channel displays enriched subcellular signals, with white arrows indicating locations. Nonresonant images show no background. Spectral linear combination algorithms separate lipids and proteins, with yellow and magenta arrows indicating LDs and nucleoli, respectively. Reproduction with permission [156]. Copyright 2020, American Chemical Society. (C) SRS imaging of phospholipids and sphingosine in human brain tissue slices. Scale bars, 10 μm (left) and 5 μm (right). Reproduction with permission [158]. Copyright 2024, Springer Nature.

In 2018, Hu et al. [155] introduced a polyacetylene-based optical supermultiplexing technique. Twenty Raman frequencies were obtained by adjusting conjugation length, isotope doping, and end-cap substitution, they generated 20 Raman frequencies. This method provides high specificity, sensitivity, and photostability for multicolor organelle imaging in single cells (Fig. 17A) and enables optical data storage and identification using barcode combinations. This material demonstrates great potential for live-cell imaging, sorting, high-throughput diagnosis, and screening. In 2020, Lee et al. [156] labeled live cancer cells with deuterated glucose (d7-glucose). Using SRS imaging to target carbon–deuterium (C–D) vibrations, glycogen incorporation was successfully visualized at the subcellular level. Using the characteristic C–D Raman peak, concentration maps of glycogen, lipids, and proteins were created for the HeLa and U87 cells (Fig. 17B). This provides a novel method for high-resolution visualization of glycogen, enhancing our understanding of cancer metabolism. In 2022, Hislop et al. [157] combined hyperspectral SRS microscopy with *k*-means clustering analysis to robustly segment organelles based on their intrinsic Raman spectra. This approach successfully analyzed LD composition in prostate cancer and healthy prostate cell models, offering a reliable, label-free tool for investigating drug–cell interactions.

Lipids are crucial for various cellular functions, making their localization and metabolic turnover essential areas of research in aging and diseases. In 2024, Zhang et al. [158] developed a penalty reference matching-SRS (PRM-SRS) to achieve label-free imaging of sphingosine and cardiolipin distributions in human brain tissue (Fig. 17C). PRM-SRS provides a novel tool for studying lipid functions across different organs and species.

SRS microscopy provides high sensitivity and molecular specificity, enabling real-time, minimally invasive imaging of live cells and tissues, which is crucial for exploring molecular dynamics in biological systems. However, several limitations persist, including relatively low sensitivity to molecules with weak Raman signals, potential background interference from nonresonant signals, and challenges in distinguishing chemically similar molecules. In addition, the complexity and high cost of optical setups, along with computationally intensive hyperspectral data analysis, restrict their widespread use. Future improvements in enhancing detection sensitivity, refining computational methods for data segmentation and analysis, and developing simplified, cost-effective instrumentation will further broaden the utility and accessibility of SRS microscopy in biological and clinical research.

### Surface-enhanced Raman spectroscopy for live-cell imaging

SERS is an optical technique that markedly enhances Raman signals using metal nanoparticles, enabling ultrasensitive detection even at the single-molecule level. In live-cell research, SERS uses nanoprobe-based targeted detection, which is a powerful tool for studying cellular functions, disease diagnostics, and pharmacokinetics [159].

**Fig. 18.**
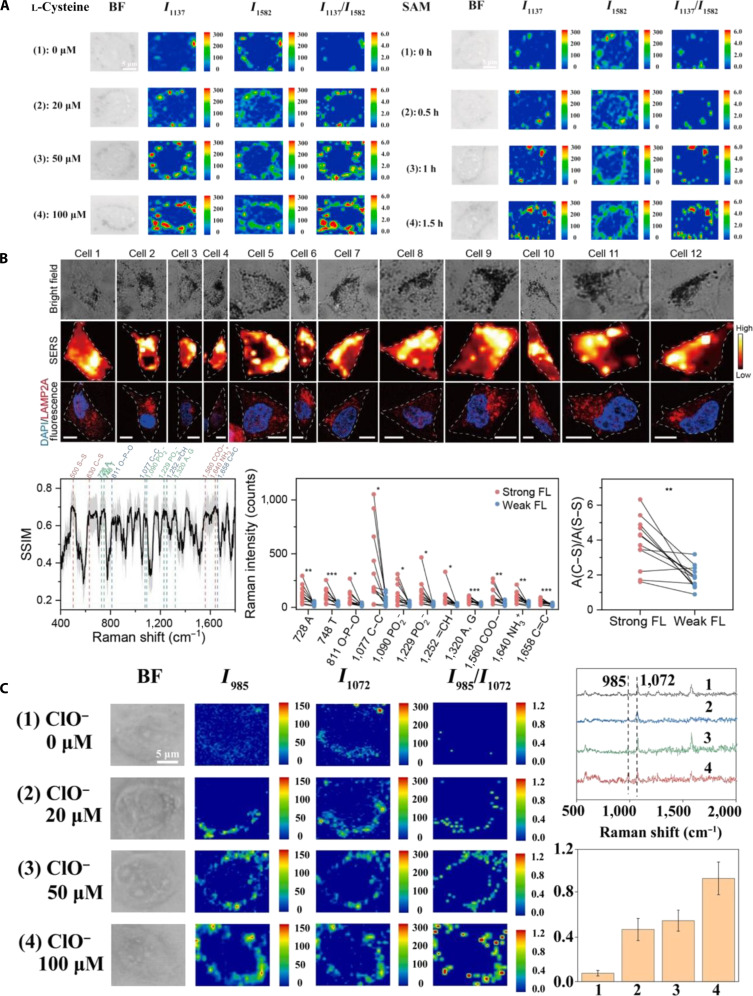
SERS cellular imaging. (A) Cell-membrane-targeted SERS imaging of H_2_S secretion: (left) concentration-dependent responses to l-cysteine (0, 20, 50, and 100 μM) and (right) time-dependent responses to SAM addition (0, 0.5, 1, and 1.5 h). Scale bars, 5 μm. Reproduction with permission [160]. Copyright 2023, Elsevier. (B) Raman spectra and SSIM analysis of single-cell lysosomal activity. Bright-field, total Raman intensity mapping (400 to 1,800 cm^−1^), and fluorescence confocal images of 12 single cells. Scale bars, 10 μm. DAPI, 4′,6-diamidino-2-phenylindole. Reproduction with permission [161]. Copyright 2024, Wiley-VCH GmbH. *P ≤ 0.05; **P ≤ 0.01; ***P ≤ 0.001. (C) Corresponding SERS spectra of cells treated with varying ClO^−^ concentrations (0, 20, 50, and 100 μM). Reproduction with permission [162]. Copyright 2025, Elsevier.

In 2024, Chen et al. [160] used a cholesterol-functionalized, membrane-targeted SERS nanoprobe for the quantitative mapping of H_2_S released from living cells. The core–shell AuNPs@4-MBN@Au probe integrates DNA–cholesterol for membrane anchoring, dithiobis(phenylazide) as an H_2_S-responsive reporter, and 4-MBN as an internal standard, achieving >85% cell viability and a 37 nM detection limit. In HeLa cells, cholesterol modification ensured efficient membrane localization and enabled the dynamic visualization of l-cysteine– or self-amplifying mRNA (SAM)-induced H_2_S production (Fig. 18A). Perturbation studies further showed that CO suppressed and NO enhanced H_2_S generation, highlighting the platform’s utility for monitoring extracellular metabolism and intercellular signaling. In 2024, Liu et al. [161] introduced a novel approach that integrated label-free SERS spectroscopy with confocal fluorescence imaging to selectively probe the lysosomal metabolic behavior of single HeLa cells. By inducing the endocytosis of SERS-active nanoparticles and applying a structural similarity (SSIM) algorithm, the method effectively identified Raman wave numbers that were highly correlated with lysosomal activity, overcoming the challenge of spectral interference from nonlysosomal regions (Fig. 18B). This strategy enabled 3D molecular profiling of lysosomes, captured macromolecular digestion events, and revealed spectral changes associated with starvation-induced autophagy, demonstrating its potential for organelle-specific metabolic monitoring. In 2025, Chen et al. [162] engineered a core–shell SERS nanosensor to simultaneously visualize the epidermal growth factor receptor (EGFR) on cell membranes and reactive oxygen species (ROS) released from living cells. Built on AuNPs@4-MBN@Au and functionalized with an EGFR aptamer plus the ROS-responsive reporter 2-MHQ (2-mercaptohydroquinon), the probe enabled multiplex SERS mapping through distinct spectral channels (Fig. 18C). Across multiple cancer cell lines, it exhibited high biocompatibility, selective membrane targeting, and real-time monitoring of EGFR and ROS dynamics. The positive correlation between ROS levels and EGFR expression, validated by Western blotting, highlights the utility of this platform for probing membrane signaling and supporting cancer diagnosis.

The advantages of SERS in live-cell applications include its ultrahigh sensitivity, which allows for the detection of molecules at low intracellular concentrations and provides rich molecular fingerprint information. Its high spatial resolution enables the precise localization and tracking of molecular dynamics, making it ideal for real-time monitoring of complex intracellular processes.

However, several challenges limit their applications [163]. To mitigate cytotoxicity from direct metal–cell contact while retaining strong SERS signals, recent studies have focused on surface engineering and metal-free platforms with clear and practical benefits. poly(ethylene glycol)ylation creates a hydrated steric barrier that suppresses protein corona formation and nonspecific membrane interactions, improving serum stability and cell viability during hour-to-day live-cell measurements. Comparative studies and reviews have consistently reported reduced aggregation and lower toxicity of polyethylene glycol (PEG)-modified AuNPs compared to uncoated controls, making PEG a sensible default material for intracellular SERS probes. Building on physical separation, lipid or biomimetic-cell-membrane coatings further insulate the plasmonic core, reduce opsonization, and improve compatibility for repeated imaging and in vivo use, with recent surveys detailing how lipid composition and the membrane phase govern nanoparticle–membrane interactions and biocompatibility. Robust core–shell encapsulation, especially Au@SiO_2_ with dense or mesoporous shells, prevents ion leakage and sharp-edge damage while preserving near-field enhancement. Live-cell SERS studies and recent material reports show that carefully tuned silica shells maintain Raman performance and markedly improve colloidal and biological stability. In parallel, biocompatible polymer interlayers, notably polydopamine (PDA), offer universal adhesion and facile biofunctionalization properties. Multiple studies have documented that PDA coatings quell cetyltrimethylammonium bromide-related toxicity in gold nanorods and stabilize hybrid Au/Ag architectures for intracellular applications. Finally, when maximum electromagnetic enhancement is not mandatory, heavy-metal-free or semiconductor SERS substrates provide lower intrinsic cytotoxicity via charge-transfer-mediated enhancement, trading some enhancement factor for improved uniformity and biosafety, which is useful for quantitative single-cell assays and longer observations. Collectively, these strategies delineate a practical path: using PEG plus silica shells for balanced intracellular performance, lipid or cell-membrane cloaks for repeat imaging or immune-sensitive contexts, and semiconductor platforms when biocompatibility and reproducibility outweigh the peak EF.

### Coherent anti-Stokes Raman spectroscopy for live-cell imaging

CARS is a nonlinear optical microscopy technique that enables label-free high-resolution chemical imaging by exciting molecular vibrational frequencies. CARS requires fluorescent labeling, allowing for the direct visualization of endogenous molecules. It is suitable for studies on lipid metabolism, cell differentiation, and membrane dynamics. Its high sensitivity and noninvasive nature make it an essential tool for studying dynamic cellular processes and chemical composition changes in the life sciences [164].

**Fig. 19.**
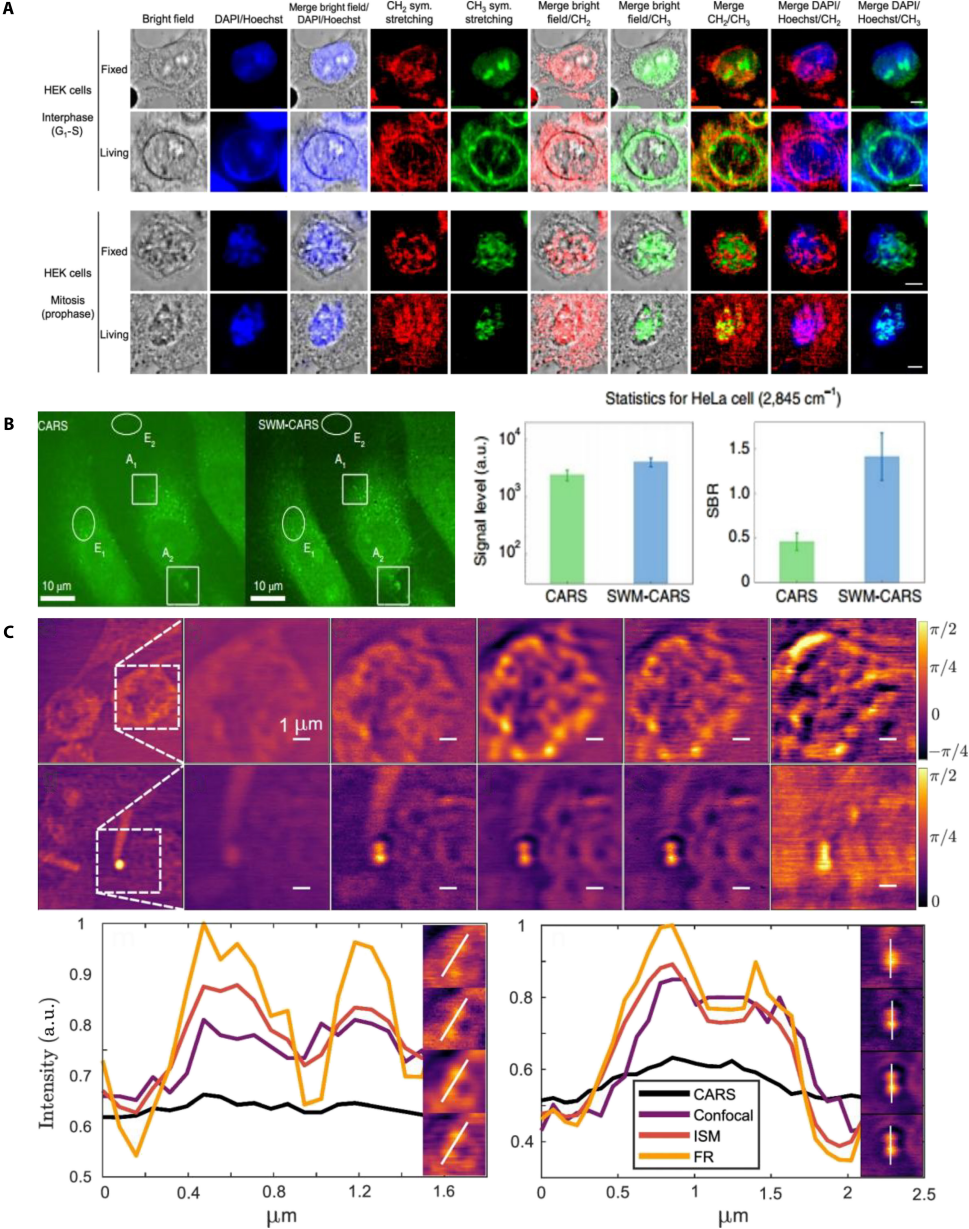
CARS cellular imaging. (A) Analysis of interphase and mitosis, staining, fixation, and live HEK293 cells (left), with standard deviation of vibrational resonant CARS signals between 2,500 and 3,200 cm^−1^ (right). Scale bars, 5 μm. Reproduction with permission [165]. Copyright 2019, Springer Nature. (B) Comparison of CARS and SWM-CARS images of HeLa cells (left). Signal levels and SBR comparison between CARS and six-wave mixing (SWM)-CARS images of HeLa cells (right). Reproduction with permission [166]. Copyright 2020, Springer Nature. (C) SR CARS imaging of HEK cells. Scale bar, 1 μm. Copyright 2019 [169], Springer Nature.

In 2019, Guerenne-Del Ben et al. [165] used hyperspectral resolution multiplexed CARS (MCARS) microscopy for cell cycle analysis. High-wave-number MCARS leverages protein vibrational signatures to visualize label-free heterochromatin condensation, enabling the real-time identification and tracking of chromosome positions and dynamics during mitosis and the resolution of intranuclear structures, such as nucleoli and nuclear boundaries, in interphase cells (Fig. 19A). This study demonstrated the ability of MCARS to detect the biochemical effects of fixation methods, providing a label-free approach for imaging cellular dynamics during chromatin rearrangement. In 2020, Gong et al. [166] used higher-order CARS (HO-CARS) microscopy to surpass the diffraction limit, enabling SR and label-free molecular imaging of biological samples. HO-CARS demonstrated superior resolution and a higher resonance-to-nonresonance background ratio. This technique has been successfully applied to unlabeled HeLa cells to achieve high-contrast, label-free, and SR vibrational imaging. Compared to traditional CARS, HO-CARS not only provides clearer intracellular structural details but also significantly enhances the signal intensity (Fig. 19B), showcasing its potential applications in biomedical systems, particularly for efficient SR imaging. In 2022, Zong et al. [167] used WF surface-enhanced CARS (WISE-CARS) microscopy to enable label-free detection of nanolabels and metabolic molecules in live cells. WISE-CARS achieved imaging speeds of 120 fps over a 130-μm × 130-μm area. The method demonstrated 3D WISE-CARS imaging of nanolabels in live cells and label-free detection of adenine released by *Staphylococcus aureus*, highlighting its potential for fast and efficient biological imaging. In 2024, Bera et al. [168] used hyperspectral CARS microscopy and multiphoton excitation FLIM to analyze the cellular uptake and expression mechanisms of SAM vaccine lipid nanoparticles (LNPs) in hamster kidney cells (BHK-21). We obtained representative broadband CARS images of BHK-21 cells treated with SAM-GFP LNPs. Using multimodal imaging, we observed increased lipid intensity in LNP-treated cells. In 2024, Zhitnitsky et al. [169] presented a novel SR CARS microscopy technique that integrated phase-resolved ISM. This approach enabled phase-sensitive imaging, distinguishing resonant from nonresonant scatterers within cells, and achieved a 1.5- to 2-fold spatial resolution enhancement compared to conventional CARS microscopy. Utilizing low-intensity excitation tuned to lipid-specific C–H vibrations (2,850 cm^−1^), this method demonstrated significantly improved contrast and clarity in human embryonic kidney (HEK) cell imaging, highlighting its potential for advanced live-cell molecular visualization (Fig. 19C).

CARS microscopy offers high spatial and temporal resolution; however, several limitations remain. Primarily, the presence of nonresonant background signals can reduce the image contrast and specificity, complicating data interpretation, especially in complex cellular environments. In addition, because of its nonlinear optical nature, CARS microscopy requires high laser intensities, which can cause photodamage and limit prolonged live-cell imaging sessions. Moreover, the complexity and expense associated with optical setups, including ultrafast laser systems and specialized detectors, may restrict their broader adoption. Finally, distinguishing between chemically similar molecules can be challenging, thereby limiting their molecular specificity in certain biological contexts. Future developments should prioritize reducing background interference, minimizing phototoxicity, simplifying and lowering instrumentation costs, and enhancing chemical specificity to further expand the effectiveness and accessibility of CARS microscopy in biomedical research.

### Emerging strategies for SR Raman imaging: Hybrid excitation and deep learning enhancement

Raman spectroscopy offers high molecular specificity and label-free imaging capability; however, its inherently weak signals and strong background noise lead to a low SNR, limiting imaging quality and speed. Stimulated Raman-excited fluorescence (SREF), developed by Xiong et al. [170], addresses this challenge by coupling vibrational excitation with fluorescence detection. A pump–Stokes pair excites specific molecular vibrations, and a probe beam converts the vibrational energy into fluorescence emission, thereby overcoming the weak signal of conventional Raman microscopy while maintaining molecular specificity. By incorporating frequency-modulated background suppression and STED-like depletion, SREF achieves high-contrast SR imaging with sub-100-nm precision. He et al. [171] proposed a noise-learning framework to address an important limitation of hyperspectral Raman microscopy, namely, the low SNR caused by complex instrument-dependent noise. By statistically learning the intrinsic noise distribution of each imaging system, including the readout and channel-specific fluctuations, the model enables quantitative noise removal directly from raw spectra.

The integration of deep learning has advanced Raman imaging by enhancing both spectral quality and spatial resolution. By learning the characteristic distribution of large hyperspectral datasets, neural networks can reconstruct high-fidelity molecular information from low-SNR spectra, markedly improving the imaging efficiency and quantitative accuracy. Building on this concept, Horgan et al. [172] developed the DeepeR framework, integrating deep-learning-based denoising with spatial SR. This advancement achieves over 90-fold faster high-throughput Raman molecular imaging, substantially improving data quality and imaging efficiency. This lays a solid foundation for the translating Raman spectroscopy from basic research to clinical diagnostics. Abdolghader et al. [173] introduced an unsupervised, one-shot deep learning framework to enhance hyperspectral SRS microscopy for cell imaging. This method simultaneously performs denoising and unsupervised segmentation on SRS images using a single hyperspectral dataset, eliminating the need for labeled training data, thereby enabling more efficient and high-fidelity cellular imaging.

The next phase of Raman nanoscopy will prioritize photon-efficient, data-driven, and integrative imaging frameworks. Hybrid excitation strategies that couple vibrational coherence control with fluorescence readout or plasmonic field confinement are expected to push the spatial resolution to the few-nanometer regime while preserving single-molecule sensitivity. In parallel, real-time AI-assisted reconstruction using transformer architectures, Bayesian denoisers, and reinforcement-learning-based acquisition control will enable adaptive sampling and quantitative signal recovery under ultralow photon budgets. The multimodal integration of SREF, SRS, and tip-enhanced Raman spectroscopy with fluorescence and EM yields correlative chemical–structural maps bridging the molecular and cellular scales. Ultimately, the establishment of open hyperspectral repositories and standardized deep learning benchmarks will unify data quality assessments and accelerate the clinical translation of quantitative Raman nanoscopy for diagnostics, metabolic imaging, and in situ molecular pathology [174].

### Applications of Raman living-cell imaging technologies for investigating label-free cell behavior

Raman imaging broadly applies to live-cell research, enabling real-time, label-free, high-resolution monitoring of intracellular molecular activities. Identifying the vibrational signatures of key molecules such as lipids, proteins, and nucleic acids provides deeper insights into physiological processes, including metabolism, signaling, and organelle function. This makes Raman imaging an indispensable tool in cellular biology [175].

**Fig. 20.**
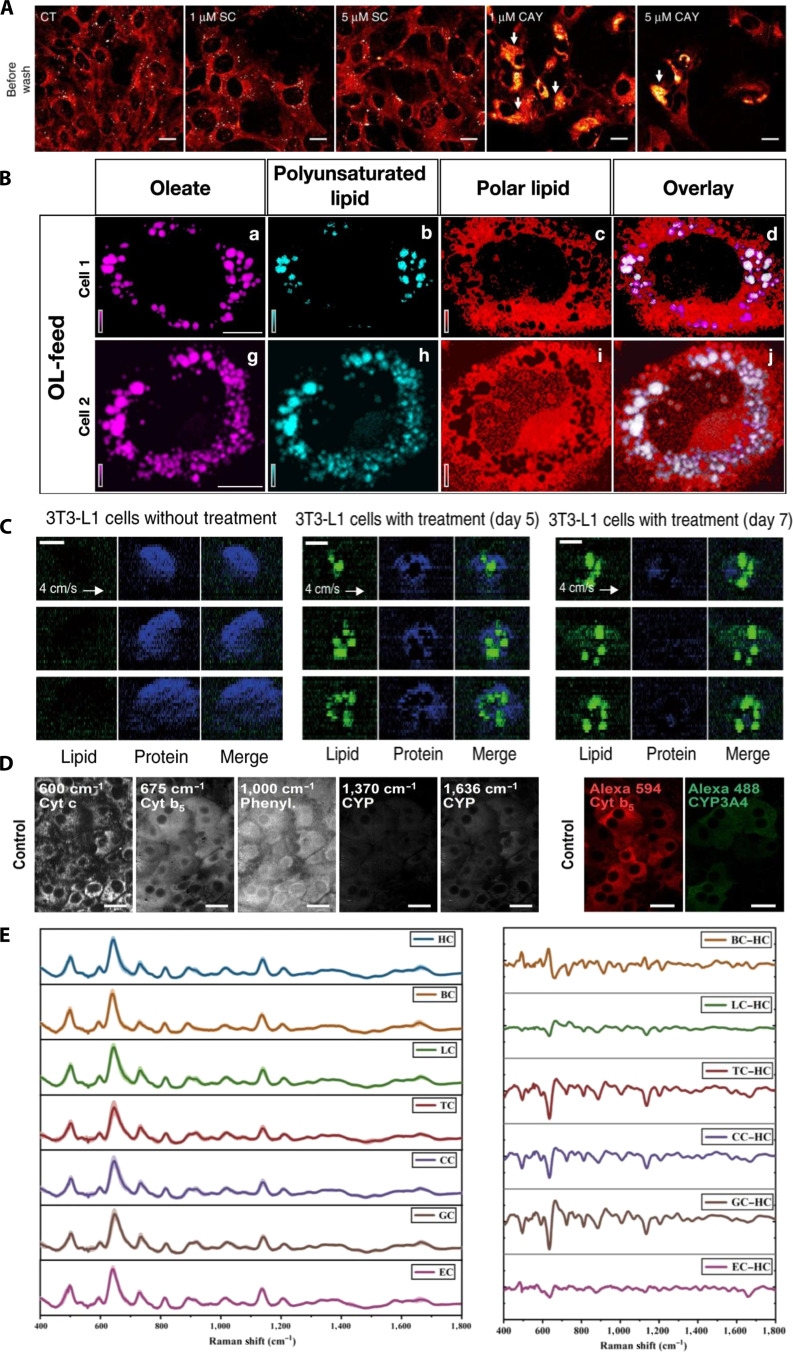
Applications of Raman in studying cellular behavior. (A) Representative SRS image of the lipid channel in M381 cells. Scale bars, 20 μm. CT, control; SC, SC 26196, a Δ6 desaturase inhibitor; CAY, CAY10566, an S CD1 (Δ9 desaturase) inhibitor. Reproduction with permission [176]. Copyright 2020, Springer Nature. (B) Distribution of lipids in HuH7 cells. Each row represents the Raman image of a cell. Scale bars, 10 μm. OL, oleate. Reproduction with permission [178]. Copyright 2020, Springer Nature. (C) SRS image of 3T3-L1 cells, showing lipid accumulation in the cytoplasm over 7 d, induced to differentiate into adipocyte-like cells. Scale bars, 10 μm. Reproduction with permission [179]. Copyright 2020, Springer Nature. (D) Reconstructed Raman images of HepaRG cells at different locations. Immunofluorescence staining of Cyt b_5_ and CYP3A4 at the same positions as the Raman measurement. Scale bars, 20 μm. Reproduction with permission [182]. Copyright 2022, Springer Nature. (E) SERS spectra and difference spectra of different types of serum samples. HC, healthy control(s); BC, breast cancer; LC, lung cancer; TC, thyroid cancer; CC, colorectal cancer; GC, gastric cancer; EC, esophageal cancer. Reproduction with permission [183]. Copyright 2025, Springer Nature.

In 2020, Du et al. [176] combined SRS microscopy with transcriptomic analysis to study the fatty acid synthesis pathway as a metabolic target for melanocyte differentiation. Hyperspectral SRS imaging revealed that targeted therapy induced apoptosis, accompanied by intracellular membrane domain phase separation (Fig. 20A). This method provides a general pathway for spatially resolved single-cell metabolomics studies. In 2021, Dodo et al. [177] explored the tumor-selective cytotoxicity mechanism of deuterated γ-linolenic acid (d7-GLA). Evaluating metabolic and toxicity effects, the study found d7-GLA suppressed metabolism and toxicity in normal fibroblasts (WI-38) but selectively enhanced cytotoxicity in tumor-derived VA-13 cells.

Raman microspectroscopy has been applied for the molecular analysis of LDs in live cells, demonstrating its powerful utility in live-cell research. In 2020, Samuel et al. [178] applied an improved multivariate curve resolution method to address background and spectral demixing challenges to achieve a one-step separation of 7 biomolecules, accurately revealing dynamic LD composition changes in HuH7 cells (Fig. 20B).

Raman imaging has shown remarkable potential for cell sorting. By leveraging the spectral signatures of intracellular molecules, label-free Raman-based cell sorting can efficiently differentiate between various cell types. Because of its high sensitivity for cancer diagnosis and biomarker detection, Raman imaging is a powerful tool in cell biology, drug discovery, and diagnostics. In 2020, Nitta et al. [179] used Raman image-activated cell sorting (RIACS) technology using ultrafast multicolor SRS microscopy to directly detect cell-specific molecular vibrations for phenotype analysis. RIACS technology processes approximately 100 events per second, is label-free, and can be applied to diverse cell types and sizes, significantly expanding its scope of application. This enabled the monitoring of lipid accumulation in 3T3-L1 cells (Fig. 20C), demonstrating its potential in cell differentiation and metabolism studies. In 2020, Hsu et al. [180] established a label-free, noninvasive single-cell Raman spectroscopy platform to classify neuronal lineages derived from human induced pluripotent stem cells (hiPSCs). Large-scale Raman spectral analysis differentiated hiPSCs from their neuronal derivatives based on distinct biochemical profiles and identified glycogen as a critical biomarker for neuronal differentiation. Conventional assays validated these findings. This versatile platform and its biomarkers hold promise for broader applications in developmental biology and glycogen metabolism disorder research in the future.

Raman imaging can identify subcellular protein localization, clarify biological functions and processes, and aid in drug development. However, because of spectral overlap between different proteins, accurate prediction of subcellular protein localization remains challenging. To address this, in 2022, Wei et al. [181] proposed a multiparallel fusion network combined with nonlinear decomposition algorithms to automatically detect subcellular structures. This model demonstrated high precision in simultaneously predicting multiple subcellular components using lung cancer cell datasets, thus providing a new method for time-resolved studies of subcellular structures.

Beyond cell differentiation, development, and disease processes (including cancer and neurodegeneration), Raman imaging has revealed changes in cellular behavior under various chemical conditions. It is also widely used in drug transport and metabolism studies to determine the absorption, distribution, and metabolic pathways of drugs within cells. Real-time tracking of drug molecules clarifies the mechanisms of drug action and provides critical data for drug development and precision therapies. In 2022, Li et al. [182] introduced a new method for the nondestructive visualization and quantification of cytochrome P450 (CYP) activity in cells using Raman microscopy under drug administration conditions. Redox- and spin-state-sensitive Raman measurements revealed that CYP was induced in hepatocytes in the oxidized, low-spin state (Fig. 20D), confirming its monooxygenase activity. In addition, glycogen depletion associated with CYP induction was observed, suggesting an impact on glucose metabolism. This nondestructive, quantitative chemical imaging method enables the assessment of CYP activity at the single-cell level, supporting drug development and research on the effects of drug regulation on CYP activity.

Raman spectroscopy has emerged as a powerful tool for the early detection of cancer, intraoperative guidance, and treatment monitoring. In tissue diagnostics, it enables the real-time identification of malignant lesions and surgical margins across various cancer types, providing molecular contrast without labeling. In liquid biopsies, Raman and SERS analyses of blood and serum facilitate the highly specific detection of tumor biomarkers, circulating tumor cells, and exosomes. Moreover, the development of Raman-based endoscopic and fiber optic probes has extended their use to in vivo applications, enabling noninvasive diagnosis and intraoperative decision-making.

Lin et al. [183] demonstrated that serum SERS combined with deep learning enabled the early detection of multiple cancers. In a large-scale case–control study (Fig. 20E), CNN-based analysis of serum Raman spectra achieved high accuracy and specificity in distinguishing patients with cancer from healthy controls. Wen et al. [184] developed a targeted SERS imaging approach for the real-time intraoperative detection and removal of microscopic breast tumors. Using tumor-specific SERS nanoparticles, this method precisely delineates tumor margins and enables complete resection during breast-conserving surgery, offering a highly sensitive and minimally invasive tool for precision cancer surgery.

Raman imaging demonstrates broad applicability and unique advantages in live-cell research by enabling label-free high-resolution visualization of intracellular molecular activities. Advanced Raman techniques, including SRS, facilitate studies on cellular metabolism, particularly glycogen incorporation and lipid dynamics, and provide valuable insights into cancer metabolism and cellular differentiation. In addition, Raman microspectroscopy effectively analyzes LD composition and protein localization, contributing significantly to the understanding of cellular functions and mechanisms. Moreover, Raman-based technologies, such as RIACS, allow precise cell sorting and phenotyping without fluorescent labels, supporting various applications ranging from drug metabolism to developmental biology. These powerful capabilities underscore the critical role of Raman imaging in uncovering cellular processes and advancing biomedical research, despite ongoing challenges in sensitivity, spectral overlap, and technical complexity.

## Concluding Remarks and Future Directions

### Concluding remarks

To illustrate the practical performance and limitations of these imaging tools, Table 3 presents a comparative analysis of the trade-offs between resolution, photodamage, and biocompatibility. This comparison highlights that future progress requires more than just higher spatial resolution; it relies on the integration of AI-driven reconstruction, standardized biocompatible probes, and multimodal frameworks to achieve high-fidelity, low-toxicity live-cell analysis.

**Table 3.** Trade-offs among resolution, photodamage, and biocompatibility in advanced imaging techniques

Technique	Spatial/temporal resolution	Photodamage risk	Biocompatibility/live-cell suitability	Trade-off characteristics	Recent mitigation strategies
STED	~30–50 nm lateral; millisecond to second frame rate	High	Moderate; photobleaching and heating limit long-term imaging	Exceptional spatial resolution achieved at the expense of light dose and cellular stress	Low-power or adaptive depletion beams, time-gated detection, event-triggered or AI-controlled beam modulation, photostable nanoprobes
SIM	~100–120 nm lateral; up to 100 fps	Low to moderate	Excellent; suitable for long-term live-cell imaging	Balanced compromise between resolution, speed, and phototoxicity	DL-based reconstruction (U-Net SIM, DL-SIM^2^), hybrid optical–computational SIM, low-photon excitation schemes
SMLM	10–20 nm localization precision; seconds to minutes per frame	Moderate	Limited by labeling density and fluorophore fatigue	Superior resolution but slow acquisition and labeling dependence	High-speed sCMOS imaging, AI-assisted localization, multiplexed DNA-PAINT, live-cell compatible fluorophores
Raman	~80–200 nm; rapid spectral acquisition possible	Low to moderate	High (label-free or biocompatible probes)	Trade-off between weak signal intensity and imaging speed/resolution	Hyperspectral SRS + DL reconstruction for SNR enhancement, frequency-modulated background suppression (SREF), biocompatible plasmonic substrates (SERS), hybrid excitation design

In practice, fluorescence images frequently contain artifacts—such as uneven illumination or motion blur—that compromise data quality. To address this, Rehn et al. [185] developed a machine learning approach using convolutional autoencoders to automatically detect and correct these errors without requiring labeled datasets. However, deep learning models can sometimes generate “hallucinations”—artificial structures that appear real but do not exist. To mitigate this risk, Liu et al. [186] recently introduced a Bayesian framework that quantifies uncertainty, allowing researchers to distinguish between trustworthy data and potential artifacts.

Beyond software, hardware innovation is also advancing data quality. A new flexible topo-optical sensing technology with ultrahigh contrast has emerged. This sensor uses elastic folded surfaces to generate sharp optical signals under strain. Although originally designed for physical sensing, this concept demonstrates how high-contrast mechanisms can be integrated into imaging systems to enhance signal quality directly at the source [187].

Despite these advances, no single imaging modality can capture the full spectrum of biological complexity, from molecular interactions to organ-level functions. Therefore, integrating complementary techniques—such as optical, electron, and magnetic resonance imaging—is essential. This “multimodal” approach links structural, chemical, and functional information to provide a comprehensive view. However, implementing these workflows remains difficult. As Bischof et al. [188] note, physical incompatibilities between instruments often damage delicate samples during transfer. Furthermore, the field lacks standardized probes that function robustly across different platforms. Establishing community standards to compare performance across fluorescence, Raman, magnetic resonance imaging, and positron emission tomography systems is the key to building reliable, high-fidelity multimodal imaging [185,188–191].

### Future directions

High-precision in situ live-cell imaging is crucial for understanding cellular behavior, development, and the mechanisms of disease. Future advancements in live-cell in situ imaging promise breakthroughs and innovations, focusing on the following key areas:

1. Enhancing spatial resolution: Advancing spatial resolution, particularly at the organelle and molecular levels, allows for clear visualization of intracellular structures and molecular interactions. This will deepen our understanding of cellular functions and biological processes.
2. Precision molecular imaging and recognition: Development of precise molecular imaging methods and highly sensitive probes enables simultaneous multimolecule detection within cells. This will allow a more precise assessment of changes in the intracellular microenvironment and function, thus providing rich information for cell biology studies.
3. Enhancing temporal resolution and long-term imaging capabilities to dynamically monitor intracellular changes, biomolecular dynamics, and functional evolution is essential. This will enable a deeper understanding of dynamic cellular behavior.
4. Multitechnology integration and fusion: Integrating multiple imaging technologies combines their strengths and achieves comprehensive insights that are impossible with single-method approaches. For example, combining SR microscopy with Raman imaging can simultaneously provide precise molecular information, spatial localization, and rapid dynamic change detection, thereby providing a more comprehensive understanding of the biological, physical, and chemical mechanisms underlying cellular changes.
5. Development of novel probes and molecular signatures: Development of novel probes and molecular signatures enables the broader application of advanced detection technologies in diverse biomedical fields. This will promote the widespread use of these imaging technologies in biomedical research.
6. Combining image analysis with AI: Integrating advanced image analysis with AI rapidly characterizes cellular states and accelerates platform development and software tool availability. This will enable researchers to master and apply these novel imaging techniques, thereby accelerating the progress of in situ cell function research.
7. Expanding application areas: Applications in embryonic development, proliferation, differentiation, cellular aging, disease mechanisms, and tumor biology advance the understanding of human health. In-depth research will reveal the processes involved in life development, enhance our understanding of disease mechanisms, and contribute to improving human health.

In conclusion, ongoing advancements in live-cell in situ imaging will significantly improve the resolution, molecular precision, real-time monitoring, technological integration, novel probes, intelligent analysis, and broaden application fields. These developments will deepen our understanding of cellular functions, accelerate life science research, and introduce innovative strategies for disease diagnosis and treatment of diseases.

## References

[1] Allport JR, Weissleder R. In vivo imaging of gene and cell therapies. Exp Hematol. 2001;29(11):1237–1246. 11698119 10.1016/s0301-472x(01)00739-1

[2] Wang Y, Shyy JY-J, Chien S. Fluorescence proteins, live-cell imaging, and mechanobiology: Seeing is believing. Annu Rev Biomed Eng. 2008;10(1):1–38. 18647110 10.1146/annurev.bioeng.010308.161731

[3] Schnell U, Dijk F, Sjollema KA, Giepmans BNG. Immunolabeling artifacts and the need for live-cell imaging. Nat Methods. 2012;9(2):152–158. 22290187 10.1038/nmeth.1855

[4] Park SC, Park MK, Kang MG. Super-resolution image reconstruction: A technical overview. IEEE Signal Process Mag. 2003;20(3):21–36.

[5] Bianchini P, Peres C, Oneto M, Galiani S, Vicidomini G, Diaspro A. STED nanoscopy: A glimpse into the future. Cell Tissue Res. 2015;360(1):143–150. 25743695 10.1007/s00441-015-2146-3 PMC4379395

[6] Samanta K, Joseph J. An overview of structured illumination microscopy: Recent advances and perspectives. J Opt. 2021;23(12): Article 123002.

[7] Wu Y-L, Tschanz A, Krupnik L, Ries J. Quantitative data analysis in single-molecule localization microscopy. Trends Cell Biol. 2020;30(11):837–851. 32830013 10.1016/j.tcb.2020.07.005

[8] Kudelski A. Analytical applications of Raman spectroscopy. Talanta. 2008;76(1):1–8. 18585231 10.1016/j.talanta.2008.02.042

[9] Willig KI, Harke B, Medda R, Hell SW. STED microscopy with continuous wave beams. Nat Methods. 2007;4(11):915–918. 17952088 10.1038/nmeth1108

[10] Mudry E, Belkebir K, Girard J, Savatier J, Le Moal E, Nicoletti C, Allain M, Sentenac A. Structured illumination microscopy using unknown speckle patterns. Nat Photonics. 2012;6(5):312–315.

[11] Jimenez A, Friedl K, Leterrier C. About samples, giving examples: Optimized single molecule localization microscopy. Methods. 2020;174:100–114. 31078795 10.1016/j.ymeth.2019.05.008

[12] Das RS, Agrawal Y. Raman spectroscopy: Recent advancements, techniques and applications. Vib Spectrosc. 2011;57(2):163–176.

[13] Kasprzycka W, Szumigraj W, Wachulak P, Trafny EA. New approaches for low phototoxicity imaging of living cells and tissues. BioEssays. 2024;46(5):2300122. 10.1002/bies.202300122 38514402

[14] Saitou T, Imamura T. Extended depth of focus two-photon light-sheet microscopy for in vivo fluorescence imaging of large multicellular organisms at cellular resolution. Int J Mol Sci. 2023;24(12):10186. 37373345 10.3390/ijms241210186 PMC10298976

[15] Vicidomini G, Bianchini P, Diaspro A. STED super-resolved microscopy. Nat Methods. 2018;15(3):173–182. 29377014 10.1038/nmeth.4593

[16] Müller T, Schumann C, Kraegeloh A. STED microscopy and its applications: New insights into cellular processes on the nanoscale. ChemPhysChem. 2012;13(8):1986–2000. 22374829 10.1002/cphc.201100986

[17] Los GV, Encell LP, McDougall MG, Hartzell DD, Karassina N, Zimprich C, Wood MG, Learish R, Ohana RF, Urh M, et al. HaloTag: A novel protein labeling technology for cell imaging and protein analysis. ACS Chem Biol. 2008;3(6):373–382. 18533659 10.1021/cb800025k

[18] Chalfie M. Green fluorescent protein. Photochem Photobiol. 1995;62(4):651–656. 7480149 10.1111/j.1751-1097.1995.tb08712.x

[19] Liang W, Bian Y, Samanta A, Chen X, Yu X, Zheng Y, Gao X, Su D. A simple yet effective H2S-activated fluorogenic probe for precise imaging of hepatitis and arthritis in situ. BMEMat. 2025;3(1): Article e12086.

[20] Patterson G, Day RN, Piston D. Fluorescent protein spectra. J Cell Sci. 2001;114(5):837–838. 11181166 10.1242/jcs.114.5.837

[21] Rankin BR, Moneron G, Wurm CA, Nelson JC, Walter A, Schwarzer D, Schroeder J, Colón-Ramos DA, Hell SW. Nanoscopy in a living multicellular organism expressing GFP. Biophys J. 2011;100(12):L63–L65. 21689517 10.1016/j.bpj.2011.05.020 PMC3123922

[22] Westphal V, Rizzoli SO, Lauterbach MA, Kamin D, Jahn R, Hell SW. Video-rate far-field optical nanoscopy dissects synaptic vesicle movement. Science. 2008;320(5873):246–249. 18292304 10.1126/science.1154228

[23] Hein B, Willig KI, Wurm CA, Westphal V, Jakobs S, Hell SW. Stimulated emission depletion nanoscopy of living cells using SNAP-tag fusion proteins. Biophys J. 2010;98(1):158–163. 20074516 10.1016/j.bpj.2009.09.053 PMC2800968

[24] Matela G, Gao P, Guigas G, Eckert AF, Nienhaus K, Nienhaus GU. A far-red emitting fluorescent marker protein, mGarnet2, for microscopy and STED nanoscopy. Chem Commun. 2017;53(5):979–982. 10.1039/c6cc09081h 28044150

[25] Willig KI, Wegner W, Müller A, Clavet-Fournier V, Steffens H. Multi-label invivo STED microscopy by parallelized switching of reversibly switchable fluorescent proteins. Cell Rep. 2021;35(9): Article 109192. 34077731 10.1016/j.celrep.2021.109192

[26] Tortarolo G, Zunino A, Fersini F, Castello M, Piazza S, Sheppard CJR, Bianchini P, Diaspro A, Koho S, Vicidomini G. Focus image scanning microscopy for sharp and gentle super-resolved microscopy. Nat Commun. 2022;13:7723. 36513680 10.1038/s41467-022-35333-y PMC9747786

[27] Tian M, Ma Y, Lin W. Fluorescent probes for the visualization of cell viability. Acc Chem Res. 2019;52(8):2147–2157. 31335119 10.1021/acs.accounts.9b00289

[28] Zhang J, Tang K, Yang Y, Yang D, Fan W. Advanced nanoprobe strategies for imaging macrophage polarization in cancer immunology. Research. 2025;8:0622. 39990770 10.34133/research.0622 PMC11842672

[29] Liu G, Peng G, Dai J, Zhou R, Wang C, Yan X, Jia X, Liu X, Gao Y, Wang L, et al. STED nanoscopy imaging of cellular lipid droplets employing a superior organic fluorescent probe. Anal Chem. 2021;93(44):14784–14791. 34704744 10.1021/acs.analchem.1c03474

[30] Liang L, Feng Z, Zhang Q, Cong TD, Wang Y, Qin X, Yi Z, Ang MJY, Zhou L, Feng H, et al. Continuous-wave near-infrared stimulated-emission depletion microscopy using downshifting lanthanide nanoparticles. Nat Nanotechnol. 2021;16(9):975–980. 34127821 10.1038/s41565-021-00927-y

[31] Gonzalez Pisfil M, Nadelson I, Bergner B, Rottmeier S, Thomae AW, Dietzel S. Stimulated emission depletion microscopy with a single depletion laser using five fluorochromes and fluorescence lifetime phasor separation. Sci Rep. 2022;12(1):14027. 35982114 10.1038/s41598-022-17825-5 PMC9388687

[32] Gao X, Cai S, Wang L, Guo Y, Liu L, Weng X, Huang K, Yan W, Qu J. Rhodamine-based fluorescent probe for dynamic STED imaging of mitochondria. Biomed Opt Express. 2024;15(3):1595–1604. 38495704 10.1364/BOE.507770 PMC10942718

[33] Ren W, Ge X, Li M, Sun J, Li S, Gao S, Shan C, Gao B, Xi P. Visualization of cristae and mtDNA interactions via STED nanoscopy using a low saturation power probe. Light Sci Appl. 2024;13(1):116. 38782912 10.1038/s41377-024-01463-9 PMC11116397

[34] Qi L, Liu S, Ping J, Yao X, Chen L, Yang D, Liu Y, Wang C, Xiao Y, Qi L, et al. Recent advances in fluorescent nanoparticles for stimulated emission depletion imaging. Biosensors. 2024;14(7):314. 39056590 10.3390/bios14070314 PMC11274644

[35] Gonzalez Pisfil M, Dietzel S. Fluorochrome separation by fluorescence lifetime phasor analysis in confocal and STED microscopy. Methods Microsc. 2025;2(1):45–60.

[36] Guo X, Pu R, Zhu Z, Qiao S, Liang Y, Huang B, Liu H, Labrador-Páez L, Kostiv U, Zhao P, et al. Achieving low-power single-wavelength-pair nanoscopy with NIR-II continuous-wave laser for multi-chromatic probes. Nat Commun. 2022;13(1):2843. 35606360 10.1038/s41467-022-30114-z PMC9126916

[37] Li X, Yu L, He M, Chen C, Yu Z, Jiang S, Wang Y, Li L, Li B, Wang G, et al. Review on carbon dots: Synthesis and application in biology field. BMEMat. 2023;1(4): Article e12045.

[38] Leménager G, de Luca E, Sun YP, Pompa PP. Super-resolution fluorescence imaging of biocompatible carbon dots. Nanoscale. 2014;6(15):8617–8623. 24983856 10.1039/c4nr01970a

[39] Li H, Guo J, Liu A, Shen X, Li J, Weng X, Liao C, He J, Liu L, Wang Y, et al. Long-wavelength excitation of carbon dots with dual-organelle targeting capability for live-cell imaging via STED nanoscopy. Dyes Pigments. 2023;216: Article 111383.

[40] Li J, Zhang L, Chen J, Zhang R, Liu Z, Zhao J, Liu B, Han MY, Han G, Zhang Z. One-step synthesized amphiphilic carbon dots for the super-resolution imaging of endoplasmic reticulum in live cells. RSC Adv. 2022;12(30):19424–19430. 35865591 10.1039/d2ra02705d PMC9255560

[41] Hanne J, Falk HJ, Görlitz F, Hoyer P, Engelhardt J, Sahl SJ, Hell SW. STED nanoscopy with fluorescent quantum dots. Nat Commun. 2015;6(1):7127. 25980788 10.1038/ncomms8127 PMC4479004

[42] Wang W, Zhao G, Kuang C, Xu L, Liu S, Sun S, Shentu P, Yang Y(M, Xu Y, Liu X. Integrated dual-color stimulated emission depletion (STED) microscopy and fluorescence emission difference (FED) microscopy. Opt Commun. 2018;423:167–174.

[43] Alvelid J, Bucci A, Testa I. Far red-shifted CdTe quantum dots for multicolour stimulated emission depletion nanoscopy. ChemPhysChem. 2023;24(3): Article e202200698. 36239140 10.1002/cphc.202200698 PMC10098508

[44] Xu L, Li Z, Ma Y, Lei L, Yue R, Cao H, Huan S, Sun W, Song G. Imaging carotid plaque burden in living mice via hybrid semiconducting polymer nanoparticles-based near-infrared-II fluorescence and magnetic resonance imaging. Research. 2023;6:0186. 39830010 10.34133/research.0186 PMC11740978

[45] Ding H, Xiao T, Ren F, Qiu Y, Shen Z, Chen X, Mijowska E, Chen H. Carbon-based nanodots for biomedical applications and clinical transformation prospects. BMEMat. 2024;2(3): Article e12085.

[46] Li Z, Gong J, Lu S, Li X, Gu X, Xu J, Khan JU, Jin D, Chen X. Photothermal lanthanide nanomaterials: From fundamentals to theranostic applications. BMEMat. 2024;2(4): Article e12088.

[47] Kang X, Jiang K, Ge S, Wei K, Zhou Y, Xu BB, Wang K, Zhang X. Frontier in advanced luminescent biomass nanocomposites for surface anticounterfeiting. ACS Nano. 2025;19(12):11547–11575. 40099949 10.1021/acsnano.4c17883

[48] Ebrahimi V, Stephan T, Kim J, Carravilla P, Eggeling C, Jakobs S, Han KY. Deep learning enables fast, gentle STED microscopy. Commun Biol. 2023;6(1):674. 37369761 10.1038/s42003-023-05054-z PMC10300082

[49] Chen Y-I, Chang YJ, Sun Y, Liao SC, Santacruz SR, Yeh HC. Spatial resolution enhancement in photon-starved STED imaging using deep learning-based fluorescence lifetime analysis. Nanoscale. 2023;15(21):9449–9456. 37159237 10.1039/d3nr00305a PMC10460507

[50] Balakrishnan A, Rahm JV, Kaminer A, Kessler LF, Barth H-D, Heilemann M. Fast and long-term super-resolution STED microscopy of nanostructural cellular dynamics using a neural network. In: *Single molecule spectroscopy and superresolution imaging XVIII*. Bellingham (WA):SPIE; 2025. p. 13–19.

[51] Rahm JV, Balakrishnan A, Wehrheim M, Kaminer A, Glogger M, Kessler LF, Kaschube M, Barth HD, Heilemann M. Fast and long-term super-resolution imaging of endoplasmic reticulum nano-structural dynamics in living cells using a neural network. Small Sci. 2025;5(1):2400385. 40212653 10.1002/smsc.202400385 PMC11935122

[52] Bilodeau A, Michaud-Gagnon A, Chabbert J, Turcotte B, Heine J, Durand A, Lavoie-Cardinal F. Development of AI-assisted microscopy frameworks through realistic simulation with pySTED. Nature Mach Intell. 2024;6(10):1197–1215. 39440349 10.1038/s42256-024-00903-w PMC11491398

[53] Zang Z, Xiao D, Wang Q, Jiao Z, Chen Y, Li DDU. Compact and robust deep learning architecture for fluorescence lifetime imaging and FPGA implementation. Methods Appl Fluoresc. 2023;11(2): Article 025002. 10.1088/2050-6120/acc0d9 36863024

[54] Ward EN, Scheeder A, Barysevich M, Kaminski CF. Self-driving microscopes: AI meets super-resolution microscopy. Small Methods. 2025;2401757. 39797467 10.1002/smtd.202401757 PMC12825358

[55] Hirtl M, Gottschalk B, Bachkoenig OA, Oflaz FE, Madreiter-Sokolowski C, Høydal MA, Graier WF. A novel super-resolution STED microscopy analysis approach to observe spatial MCU and MICU1 distribution dynamics in cells. Biochim Biophys Acta, Mol Cell Res. 2025;(3):1872: Article 119900. 10.1016/j.bbamcr.2025.119900 39765273

[56] Honigmann A, Mueller V, Ta H, Schoenle A, Sezgin E, Hell SW, Eggeling C. Scanning STED-FCS reveals spatiotemporal heterogeneity of lipid interaction in the plasma membrane of living cells. Nat Commun. 2014;5(1):5412. 25410140 10.1038/ncomms6412

[57] Kamper M, Ta H, Jensen NA, Hell SW, Jakobs S. Near-infrared STED nanoscopy with an engineered bacterial phytochrome. Nat Commun. 2018;9(1):4762. 30420676 10.1038/s41467-018-07246-2 PMC6232180

[58] Alvelid J, Damenti M, Sgattoni C, Testa I. Event-triggered STED imaging. Nat Methods. 2022;19(10):1268–1275. 36076037 10.1038/s41592-022-01588-y PMC9550628

[59] Barbotin A, Urbančič I, Galiani S, Eggeling C, Booth M, Sezgin E. z-STED imaging and spectroscopy to investigate nanoscale membrane structure and dynamics. Biophys J. 2020;118(10):2448–2457. 32359408 10.1016/j.bpj.2020.04.006 PMC7231928

[60] Liu T, Stephan T, Chen P, Keller-Findeisen J, Chen J, Riedel D, Yang Z, Jakobs S, Chen Z. Multi-color live-cell STED nanoscopy of mitochondria with a gentle inner membrane stain. Proc Natl Acad Sci USA. 2022;119(52): Article e2215799119. 36534799 10.1073/pnas.2215799119 PMC9907107

[61] Ishigaki M, Iketani M, Sugaya M, Takahashi M, Tanaka M, Hattori S, Ohsawa I. STED super-resolution imaging of mitochondria labeled with TMRM in living cells. Mitochondrion. 2016;28:79–87. 27090168 10.1016/j.mito.2016.03.009

[62] Yang X, Yang Z, Wu Z, He Y, Shan C, Chai P, Ma C, Tian M, Teng J, Jin D, et al. Mitochondrial dynamics quantitatively revealed by STED nanoscopy with an enhanced squaraine variant probe. Nat Commun. 2020;11(1):3699. 32709877 10.1038/s41467-020-17546-1 PMC7382495

[63] Willig KI, Rizzoli SO, Westphal V, Jahn R, Hell SW. STED microscopy reveals that synaptotagmin remains clustered after synaptic vesicle exocytosis. Nature. 2006;440(7086):935–939. 16612384 10.1038/nature04592

[64] Urban NT, Willig KI, Hell SW, Nägerl UV. STED nanoscopy of actin dynamics in synapses deep inside living brain slices. Biophys J. 2011;101(5):1277–1284. 21889466 10.1016/j.bpj.2011.07.027 PMC3164186

[65] Arizono M, Inavalli VVGK, Panatier A, Pfeiffer T, Angibaud J, Levet F, ter Veer MJT, Stobart J, Bellocchio L, Mikoshiba K, et al. Structural basis of astrocytic Ca^2+^ signals at tripartite synapses. Nat Commun. 2020;11(1):1906. 32312988 10.1038/s41467-020-15648-4 PMC7170846

[66] Henneberger C, Bard L, Panatier A, Reynolds JP, Kopach O, Medvedev NI, Minge D, Herde MK, Anders S, Kraev I, et al. LTP induction boosts glutamate spillover by driving withdrawal of perisynaptic astroglia. Neuron. 2020;108(5):919–936.e11. 32976770 10.1016/j.neuron.2020.08.030 PMC7736499

[67] Bergstrand J, Xu L, Miao X, Li N, Öktem O, Franzén B, Auer G, Lomnytska M, Widengren J. Super-resolution microscopy can identify specific protein distribution patterns in platelets incubated with cancer cells. Nanoscale. 2019;11(20):10023–10033. 31086875 10.1039/c9nr01967g

[68] Bigi A, Napolitano L, Vadukul DM, Chiti F, Cecchi C, Aprile FA, Cascella R. A single-domain antibody detects and neutralises toxic Aβ42 oligomers in the Alzheimer’s disease CSF. Alzheimer’s Res Ther. 2024;16(1):13. 38238842 10.1186/s13195-023-01361-z PMC10795411

[69] Johansson B, Oasa S, Muntsant Soria A, Tiiman A, Söderberg L, Amandius E, Möller C, Lannfelt L, Terenius L, Giménez-Llort L, et al. The interwoven fibril-like structure of amyloid-beta plaques in mouse brain tissue visualized using super-resolution STED microscopy. Cell Biosci. 2023;13(1):142. 37542303 10.1186/s13578-023-01086-4 PMC10403925

[70] Heintzmann R, Huser T. Super-resolution structured illumination microscopy. Chem Rev. 2017;117(23):13890–13908. 29125755 10.1021/acs.chemrev.7b00218

[71] Dan D, Yao B, Lei M. Structured illumination microscopy for super-resolution and optical sectioning. Chin Sci Bull. 2014;59:1291–1307.

[72] Ortkrass H, Schürstedt J, Wiebusch G, Szafranska K, McCourt P, Huser T. High-speed TIRF and 2D super-resolution structured illumination microscopy with a large field of view based on fiber optic components. Opt Express. 2023;31(18):29156–29165. 37710721 10.1364/OE.495353

[73] Gustafsson MG, Shao L, Carlton PM, Wang CJR, Golubovskaya IN, Cande WZ, Agard DA, Sedat JW. Three-dimensional resolution doubling in wide-field fluorescence microscopy by structured illumination. Biophys J. 2008;94(12):4957–4970. 18326650 10.1529/biophysj.107.120345 PMC2397368

[74] Xypakis E, Gosti G, Giordani T, Santagati R, Ruocco G, Leonetti M. Deep learning for blind structured illumination microscopy. Sci Rep. 2022;12(1):8623. 35597874 10.1038/s41598-022-12571-0 PMC9124205

[75] Saxena M, Eluru G, Gorthi SS. Structured illumination microscopy. Adv Opt Photon. 2015;7(2):241–275.

[76] Zhou W, Yao M, Lin X, Yu Q, Peng J, Zhong J. Confocal structured illumination microscopy for improving the signal-to-noise ratio and depth of fluorescent optical section imaging. Opt Express. 2024;32(18):32550–32563. 39573359 10.1364/OE.536711

[77] Liu Z, Luo Z, Chen H, Yin A, Sun H, Zhuang Z, Chen T. Optical section structured illumination-based Förster resonance energy transfer imaging. Cytometry A. 2022;101(3):264–272. 34490985 10.1002/cyto.a.24500

[78] Kumar V, Behrman K, Speed F, Saladrigas CA, Supekar O, Huang Z, Bright VM, Welle CG, Restrepo D, Gopinath JT, et al. MicroLED light source for optical sectioning structured illumination microscopy. Opt Express. 2023;31(10):16709–16718. 37157744 10.1364/OE.486754 PMC10316754

[79] Li J, Chen X, Wen K, An S, Zheng J, Ma Y, Wang X, Dan D, Yao B, Nienhaus GU, et al. Enhancing optical sectioning in structured illumination microscopy with axially confined fringe modulation. Laser Photonics Rev. 2025;19(10):2401697.

[80] Chen X, Lei Y, Wen K, Li J, An S, Zheng J, Kong L, Kozacki T, Ma Y, Gao P. Large-field optical sectioning structured illumination microscopy. Opt Laser Technol. 2025;181: Article 111870.

[81] Wen K, Fang X, Ma Y, Liu M, An S, Zheng JJ, Kozacki T, Gao P. Large-field structured illumination microscopy based on 2D grating and a spatial light modulator. Opt Lett. 2022;47(11):2666–2669. 35648900 10.1364/OL.460292

[82] Schermelleh L, Carlton PM, Haase S, Shao L, Winoto L, Kner P, Burke B, Cardoso MC, Agard DA, Gustafsson MGL, et al. Subdiffraction multicolor imaging of the nuclear periphery with 3D structured illumination microscopy. Science. 2008;320(5881):1332–1336. 18535242 10.1126/science.1156947 PMC2916659

[83] Fiolka R, Shao L, Rego EH, Davidson MW, Gustafsson MGL. Time-lapse two-color 3D imaging of live cells with doubled resolution using structured illumination. Proc Natl Acad Sci USA. 2012;109(14):5311–5315. 22431626 10.1073/pnas.1119262109 PMC3325651

[84] Li D, Shao L, Chen BC, Zhang X, Zhang M, Moses B, Milkie DE, Beach JR, Hammer JA III, Pasham M, et al. Extended-resolution structured illumination imaging of endocytic and cytoskeletal dynamics. Science. 2015;349(6251):aab3500. 26315442 10.1126/science.aab3500 PMC4659358

[85] Lin R, Kipreos ET, Zhu J, Khang CH, Kner P. Subcellular three-dimensional imaging deep through multicellular thick samples by structured illumination microscopy and adaptive optics. Nat Commun. 2021;12(1):3148. 34035309 10.1038/s41467-021-23449-6 PMC8149693

[86] Chang B-J, Shepherd D, Fiolka R. Projective oblique plane structured illumination microscopy. NPJ Imaging. 2023;1(1):2. 40604244 10.1038/s44303-023-00002-2 PMC12118692

[87] Mo Y, Wang K, Li L, Xing S, Ye S, Wen J, Duan X, Luo Z, Gou W, Chen T, et al. Quantitative structured illumination microscopy via a physical model-based background filtering algorithm reveals actin dynamics. Nat Commun. 2023;14(1):3089. 37248215 10.1038/s41467-023-38808-8 PMC10227022

[88] Temma K, Oketani R, Kubo T, Bando K, Maeda S, Sugiura K, Matsuda T, Heintzmann R, Kaminishi T, Fukuda K, et al. Selective-plane-activation structured illumination microscopy. Nat Methods. 2024;21(5):889–896. 38580844 10.1038/s41592-024-02236-3

[89] Chen Q, Gou W, Lu W, Li J, Wei Y, Li H, Wang C, You W, Li Z, Dong D, et al. Fast, three-dimensional, live-cell super-resolution imaging with multiplane structured illumination microscopy. Nat Photonics. 2025;19:567–576.

[90] Kraus F, Miron E, Demmerle J, Chitiashvili T, Budco A, Alle Q, Matsuda A, Leonhardt H, Schermelleh L, Markaki Y. Quantitative 3D structured illumination microscopy of nuclear structures. Nat Protoc. 2017;12(5):1011–1028. 28406495 10.1038/nprot.2017.020

[91] Choi J, Kim HJ, Sim G, Lee S, Park WS, Park JH, Kang HY, Lee M, Heo WD, Choo J, et al., Label-free three-dimensional analyses of live cells with deep-learning-based segmentation exploiting refractive index distributions. bioRxiv. 2021. 10.1101/2021.05.23.445351

[92] Zheng Y, Chen J, Wu C, Gong W, Si K. Adaptive optics for structured illumination microscopy based on deep learning. Cytometry A. 2021;99(6):622–631. 33543823 10.1002/cyto.a.24319

[93] Zhang Q, Chen J, Li J, Bo E, Jiang H, Lu X, Zhong L, Tian J. Deep learning-based single-shot structured illumination microscopy. Opt Lasers Eng. 2022;155: Article 107066.

[94] Jin L, Liu B, Zhao F, Hahn S, Dong B, Song R, Elston TC, Xu Y, Hahn KM. Deep learning enables structured illumination microscopy with low light levels and enhanced speed. Nat Commun. 2020;11(1):1934. 32321916 10.1038/s41467-020-15784-x PMC7176720

[95] Qiao C, Li D, Liu Y, Zhang S, Liu K, Liu C, Guo Y, Jiang T, Fang C, Li N, et al. Rationalized deep learning super-resolution microscopy for sustained live imaging of rapid subcellular processes. Nat Biotechnol. 2023;41(3):367–377. 36203012 10.1038/s41587-022-01471-3

[96] Wang J, Fan J, Zhou B, Huang X, Chen L. Hybrid reconstruction of the physical model with the deep learning that improves structured illumination microscopy. Adv Photonics Nexus. 2023;2(1):016012.

[97] Song L, Liu X, Xiong Z, Ahamed M, An S, Zheng J, Ma Y, Gao P. Super-resolution reconstruction of structured illumination microscopy using deep-learning and sparse deconvolution. Opt Lasers Eng. 2024;174: Article 107968.

[98] Tian W, Chen R, Chen L. Computational super-resolution: An odyssey in harnessing priors to enhance optical microscopy resolution. Anal Chem. 2025;97(9):4763–4792. 40013618 10.1021/acs.analchem.4c07047 PMC11912138

[99] Qian J, Wang C, Wu H, Chen Q, Zuo C. Ensemble deep learning-enabled single-shot composite structured illumination microscopy (eDL-cSIM). PhotoniX. 2025;6(1):13.

[100] Liu X, Li J, Song L, Zhuo K, Wen K, An S, Ma Y, Zheng J, Gao P. A denoise network for structured illumination microscopy with low-light exposure. Photonics. 2024;11(8):776.

[101] Sun Y, Gu S, Ma Y, Song A, Xing L, Niu J, Yang R, Hu X, Wang W, Ma T, et al. Platelet ultrastructural changes stored at room temperature versus cold storage observed by electron microscopy and structured illumination microscopy. Exp Hematol. 2025;141: Article 104671. 39521173 10.1016/j.exphem.2024.104671

[102] Sonnen KF, Schermelleh L, Leonhardt H, Nigg EA. 3D-structured illumination microscopy provides novel insight into architecture of human centrosomes. Biol Open. 2012;1(10):965–976. 23213374 10.1242/bio.20122337 PMC3507176

[103] Komis G, Mistrik M, Šamajová O, Doskočilová A, Ovečka M, Illés P, Bartek J, Šamaj J. Dynamics and organization of cortical microtubules as revealed by superresolution structured illumination microscopy. Plant Physiol. 2014;165(1):129–148. 24686112 10.1104/pp.114.238477 PMC4012574

[104] Kubalová I, Němečková A, Weisshart K, Hřibová E, Schubert V. Comparing super-resolution microscopy techniques to analyze chromosomes. Int J Mol Sci. 2021;22(4):1903. 33672992 10.3390/ijms22041903 PMC7917581

[105] Rodermund L, Coker H, Oldenkamp R, Wei G, Bowness J, Rajkumar B, Nesterova T, Susano Pinto DM, Schermelleh L, Brockdorff N. Time-resolved structured illumination microscopy reveals key principles of Xist RNA spreading. Science. 2021;372(6547):eabe7500. 34112668 10.1126/science.abe7500

[106] Schouten M, de Luca GMR, Alatriste González DK, de Jong BE, Timmermans W, Xiong H, Krugers H, Manders EMM, Fitzsimons CP. Imaging dendritic spines of rat primary hippocampal neurons using structured illumination microscopy. J Visual Exp. 2014;87:51276. 10.3791/51276 PMC4172253 24835130

[107] Li X, Wu Y, Su Y, Rey-Suarez I, Matthaeus C, Updegrove TB, Wei Z, Zhang L, Sasaki H, Li Y, et al. Three-dimensional structured illumination microscopy with enhanced axial resolution. Nat Biotechnol. 2023;41(9):1307–1319. 36702897 10.1038/s41587-022-01651-1 PMC10497409

[108] Huang X, Fan J, Li L, Liu H, Wu R, Wu Y, Wei L, Mao H, Lal A, Xi P, et al. Fast, long-term, super-resolution imaging with Hessian structured illumination microscopy. Nat Biotechnol. 2018;36(5):451–459. 29644998 10.1038/nbt.4115

[109] Opstad IS, Godtliebsen G, Ahluwalia BS, Myrmel T, Agarwal K, Birgisdottir ÅB. Mitochondrial dynamics and quantification of mitochondria-derived vesicles in cardiomyoblasts using structured illumination microscopy. J Biophotonics. 2022;15(2): Article e202100305. 34766731 10.1002/jbio.202100305

[110] Zhao W, Zhao S, Li L, Huang X, Xing S, Zhang Y, Qiu G, Han Z, Shang Y, Sun DE, et al. Sparse deconvolution improves the resolution of live-cell super-resolution fluorescence microscopy. Nat Biotechnol. 2022;40(4):606–617. 34782739 10.1038/s41587-021-01092-2

[111] Xu P, Deng H, Hong Z, Zhong S, Chen F, Wang L, Wang Z, Mei Y, Luo Z, He Z, et al. Superresolution fluorescence microscopy of platelet subcellular structures as a potential tumor liquid biopsy. Small Methods. 2023;7(10):2300445. 10.1002/smtd.202300445 37349902

[112] Vangindertael J, Camacho R, Sempels W, Mizuno H, Dedecker P, Janssen KPF. An introduction to optical super-resolution microscopy for the adventurous biologist. Methods Appl Fluoresc. 2018;6(2): Article 022003. 29422456 10.1088/2050-6120/aaae0c

[113] Nieves DJ, Gaus K, Baker MA. DNA-based super-resolution microscopy: DNA-PAINT. Genes. 2018;9(12):621. 30544986 10.3390/genes9120621 PMC6315775

[114] Huang B, Wang W, Bates M, Zhuang X. Three-dimensional super-resolution imaging by stochastic optical reconstruction microscopy. Science. 2008;319(5864):810–813. 18174397 10.1126/science.1153529 PMC2633023

[115] Bates M, Jones SA, Zhuang X. Stochastic optical reconstruction microscopy (STORM): A method for superresolution fluorescence imaging. Cold Spring Harb Protoc. 2013;2013(6):489–520. 10.1101/pdb.top075143 23734025

[116] Ricci MA, Manzo C, García-Parajo MF, Lakadamyali M, Cosma MP. Chromatin fibers are formed by heterogeneous groups of nucleosomes in vivo. Cell. 2015;160(6):1145–1158. 25768910 10.1016/j.cell.2015.01.054

[117] Bintu B, Mateo LJ, Su JH, Sinnott-Armstrong NA, Parker M, Kinrot S, Yamaya K, Boettiger AN, Zhuang X. Super-resolution chromatin tracing reveals domains and cooperative interactions in single cells. Science. 2018;362(6413):eaau1783. 30361340 10.1126/science.aau1783 PMC6535145

[118] Diekmann R, Kahnwald M, Schoenit A, Deschamps J, Matti U, Ries J. Optimizing imaging speed and excitation intensity for single-molecule localization microscopy. Nat Methods. 2020;17(9):909–912. 32807954 10.1038/s41592-020-0918-5 PMC7610360

[119] Wu W, Luo S, Fan C, Yang T, Zhang S, Meng W, Xu T, Ji W, Gu L. Tetra-color superresolution microscopy based on excitation spectral demixing. Light Sci Appl. 2023;12(1):9. 36588110 10.1038/s41377-022-01054-6 PMC9806106

[120] Sengupta P, Van Engelenburgˆ SB, Lippincott-Schwartz J. Superresolution imaging of biological systems using photoactivated localization microscopy. Chem Rev. 2014;114(6):3189–3202. 24417572 10.1021/cr400614m PMC4221852

[121] Hess ST, Girirajan TP, Mason MD. Ultra-high resolution imaging by fluorescence photoactivation localization microscopy. Biophys J. 2006;91(11):4258–4272. 16980368 10.1529/biophysj.106.091116 PMC1635685

[122] Kanchanawong P, Shtengel G, Pasapera AM, Ramko EB, Davidson MW, Hess HF, Waterman CM. Nanoscale architecture of integrin-based cell adhesions. Nature. 2010;468(7323):580–584. 21107430 10.1038/nature09621 PMC3046339

[123] Jensen LG, Hoh TY, Williamson DJ, Griffié J, Sage D, Rubin-Delanchy P, Owen DM. Correction of multiple-blinking artifacts in photoactivated localization microscopy. Nat Methods. 2022;19(5):594–602. 35545712 10.1038/s41592-022-01463-w

[124] Parteka-Tojek Z, Zhu JJ, Lee B, Jodkowska K, Wang P, Aaron J, Chew TL, Banecki K, Plewczynski D, Ruan Y. Super-resolution visualization of chromatin loop folding in human lymphoblastoid cells using interferometric photoactivated localization microscopy. Sci Rep. 2022;12(1):8582. 35595799 10.1038/s41598-022-12568-9 PMC9122977

[125] Hou Y, Laasmaa M, Li J, Shen X, Manfra O, Nordén ES, le C, Zhang L, Sjaastad I, Jones PP, et al. Live-cell photoactivated localization microscopy correlates nanoscale ryanodine receptor configuration to calcium sparks in cardiomyocytes. Nat Cardiovascul Res. 2023;2(3):251–267. 10.1038/s44161-022-00199-2 PMC7616007 38803363

[126] Filius M, Cui TJ, Ananth AN, Docter MW, Hegge JW, van der Oost J, Joo C. High-speed super-resolution imaging using protein-assisted DNA-PAINT. Nano Lett. 2020;20(4):2264–2270. 32168456 10.1021/acs.nanolett.9b04277 PMC7146856

[127] Jungmann R, Avendaño MS, Woehrstein JB, Dai M, Shih WM, Yin P. Multiplexed 3D cellular super-resolution imaging with DNA-PAINT and exchange-PAINT. Nat Methods. 2014;11(3):313–318. 24487583 10.1038/nmeth.2835 PMC4153392

[128] Schueder F, Lara-Gutiérrez J, Beliveau BJ, Saka SK, Sasaki HM, Woehrstein JB, Strauss MT, Grabmayr H, Yin P, Jungmann R. Multiplexed 3D super-resolution imaging of whole cells using spinning disk confocal microscopy and DNA-PAINT. Nat Commun. 2017;8(1):2090. 29233999 10.1038/s41467-017-02028-8 PMC5727263

[129] Brockman JM, Su H, Blanchard AT, Duan Y, Meyer T, Quach ME, Glazier R, Bazrafshan A, Bender RL, Kellner AV, et al. Live-cell super-resolved PAINT imaging of piconewton cellular traction forces. Nat Methods. 2020;17(10):1018–1024. 32929270 10.1038/s41592-020-0929-2 PMC7574592

[130] Narayanasamy KK, Rahm JV, Tourani S, Heilemann M. Fast DNA-PAINT imaging using a deep neural network. Nat Commun. 2022;13(1):5047. 36030338 10.1038/s41467-022-32626-0 PMC9420107

[131] Banerjee A, Anand M, Kalita S, Ganji M. Single-molecule analysis of DNA base-stacking energetics using patterned DNA nanostructures. Nat Nanotechnol. 2023;18(12):1474–1482. 37591937 10.1038/s41565-023-01485-1 PMC10716042

[132] Steen PR, Unterauer EM, Masullo LA, Kwon J, Perovic A, Jevdokimenko K, Opazo F, Fornasiero EF, Jungmann R. The DNA-PAINT palette: A comprehensive performance analysis of fluorescent dyes. Nat Methods. 2024;21(9):1755–1762. 39112798 10.1038/s41592-024-02374-8 PMC11399092

[133] Lycas MD, Manley S. DNA-PAINT adaptors make for efficient multiplexing. Cell reports. Methods. 2024;4(6). 10.1016/j.crmeth.2024.100801 PMC11228366 38889688

[134] Piantanida L, Li IT, Hughes WL. Advancements in DNA-PAINT: Applications and challenges in biological imaging and nanoscale metrology. Nanoscale. 2025;17(23):14016–14034. 40407724 10.1039/d4nr04544k

[135] Hyun Y, Kim D. Artificial intelligence-empowered spectroscopic single molecule localization microscopy. Small Methods. 2024;2401654. 10.1002/smtd.202401654 39593255

[136] Gaire SK, Daneshkhah A, Flowerday E, Gong R, Frederick J, Backman V. Deep learning-based spectroscopic single-molecule localization microscopy. J Biomed Opt. 2024;29(6):066501. 38799979 10.1117/1.JBO.29.6.066501 PMC11122423

[137] Chen R, Tang X, Zhao Y, Shen Z, Zhang M, Shen Y, Li T, Chung CHY, Zhang L, Wang J, et al. Single-frame deep-learning super-resolution microscopy for intracellular dynamics imaging. Nat Commun. 2023;14(1):2854. 37202407 10.1038/s41467-023-38452-2 PMC10195829

[138] Basumatary J, Aravinth S, Pant N, Ramanathan V, Thakur CS, Mondal PP. Event-based single molecule localization microscopy (eventSMLM) for high spatio-temporal super-resolution imaging. bioRxiv. 2023. 10.1101/2023.12.30.573392

[139] Xu D, Gu Y, Lu J, Xu L, Wang W, Dong B. Deep-learning-assisted spectroscopic single-molecule localization microscopy based on spectrum-to-spectrum denoising. Nanoscale. 2024;16(11):5729–5736. 38407360 10.1039/d3nr05870k

[140] Cabillic M, Forriere H, Bettarel L, Butler C, Neuhaus A, Idrissi I, Sambrano-Lopez ME, Rossbroich J, Müller LR, Ries J, et al. In-depth single molecule localization microscopy using adaptive optics and single objective light-sheet microscopy. Nat Commun. 2025;16(1):8362. 40993142 10.1038/s41467-025-62198-8 PMC12460799

[141] Breton V, Nazac P, Boulet D, Danglot L. Molecular mapping of neuronal architecture using STORM microscopy and new fluorescent probes for SMLM imaging. Neurophotonics. 2024;11(1):014414. 38464866 10.1117/1.NPh.11.1.014414 PMC10923464

[142] Jolivet N, Bertolin G. Revealing mitochondrial architecture and functions with single molecule localization microscopy. Biol Cell. 2025;117(1): Article e2400082. 39877953 10.1111/boc.202400082 PMC11775716

[143] Chen C, Zong S, Wang Z, Lu J, Zhu D, Zhang Y, Cui Y. Imaging and intracellular tracking of cancer-derived exosomes using single-molecule localization-based super-resolution microscope. ACS Appl Mater Interfaces. 2016;8(39):25825–25833. 27617891 10.1021/acsami.6b09442

[144] Yan R, Chen K, Xu K. Probing nanoscale diffusional heterogeneities in cellular membranes through multidimensional single-molecule and super-resolution microscopy. J Am Chem Soc. 2020;142(44):18866–18873. 33084318 10.1021/jacs.0c08426 PMC8447239

[145] Ejdrup AL, Lycas MD, Lorenzen N, Konomi A, Herborg F, Madsen KL, Gether U. A density-based enrichment measure for assessing colocalization in single-molecule localization microscopy data. Nat Commun. 2022;13(1):4388. 35902578 10.1038/s41467-022-32064-y PMC9334352

[146] Ruba A, Tingey M, Luo W, Yu J, Evangelou A, Higgins R, Khim S, Yang W. The ciliary lumen accommodates passive diffusion and vesicle-assisted trafficking in cytoplasm–ciliary transport. Mol Biol Cell. 2023;34(6):ar59. 36857170 10.1091/mbc.E22-10-0452 PMC10208104

[147] Massou S, Nunes Vicente F, Wetzel F, Mehidi A, Strehle D, Leduc C, Voituriez R, Rossier O, Nassoy P, Giannone G. Cell stretching is amplified by active actin remodelling to deform and recruit proteins in mechanosensitive structures. Nat Cell Biol. 2020;22(8):1011–1023. 32719553 10.1038/s41556-020-0548-2

[148] Schlichthaerle T, Lindner C, Jungmann R. Super-resolved visualization of single DNA-based tension sensors in cell adhesion. Nat Commun. 2021;12(1):2510. 33947854 10.1038/s41467-021-22606-1 PMC8097079

[149] Raciti G, Cavallaro G, Giuffrida R, Grange C, Leggio L, Catania M, Iraci N, Bruno E, Giaimi LA, Lombardo SP, et al. Single-vesicle molecular profiling by dSTORM imaging in a liquid biopsy assay predicts early relapse in colorectal cancer. Biomolecules. 2025;15(9):1307. 41008614 10.3390/biom15091307 PMC12467539

[150] Antunes-Ferreira M, Koppers-Lalic D, Würdinger T. Circulating platelets as liquid biopsy sources for cancer detection. Mol Oncol. 2021;15(6):1727–1743. 33219615 10.1002/1878-0261.12859 PMC8169446

[151] Zheng S, Dadina N, Mozumdar D, Lesiak L, Martinez KN, Miller EW, Schepartz A. Long-term super-resolution inner mitochondrial membrane imaging with a lipid probe. Nat Chem Biol. 2024;20(1):83–92. 37857992 10.1038/s41589-023-01450-y PMC10746544

[152] Saguy A, Alalouf O, Opatovski N, Jang S, Heilemann M, Shechtman Y. DBlink: Dynamic localization microscopy in super spatiotemporal resolution via deep learning. Nat Methods. 2023;20(12):1939–1948. 37500760 10.1038/s41592-023-01966-0

[153] Pezzotti G. Raman spectroscopy in cell biology and microbiology. J Raman Spectrosc. 2021;52(12):2348–2443.

[154] Zhang D, Wang P, Slipchenko MN, Cheng JX. Fast vibrational imaging of single cells and tissues by stimulated Raman scattering microscopy. Acc Chem Res. 2014;47(8):2282–2290. 24871269 10.1021/ar400331q PMC4139189

[155] Hu F, Zeng C, Long R, Miao Y, Wei L, Xu Q, Min W. Supermultiplexed optical imaging and barcoding with engineered polyynes. Nat Methods. 2018;15(3):194–200. 29334378 10.1038/nmeth.4578 PMC5831481

[156] Lee D, du J, Yu R, Su Y, Heath JR, Wei L. Visualizing subcellular enrichment of glycogen in live cancer cells by stimulated Raman scattering. Anal Chem. 2020;92(19):13182–13191. 32907318 10.1021/acs.analchem.0c02348 PMC10676777

[157] Hislop EW, Tipping WJ, Faulds K, Graham D. Label-free imaging of lipid droplets in prostate cells using stimulated Raman scattering microscopy and multivariate analysis. Anal Chem. 2022;94(25):8899–8908. 35699644 10.1021/acs.analchem.2c00236 PMC9244870

[158] Zhang W, Li Y, Fung AA, Li Z, Jang H, Zha H, Chen X, Gao F, Wu JY, Sheng H, et al. Multi-molecular hyperspectral PRM-SRS microscopy. Nat Commun. 2024;15(1):1599. 38383552 10.1038/s41467-024-45576-6 PMC10881988

[159] Xie W, Su L, Shen A, Materny A, Hu J. Application of surface-enhanced Raman scattering in cell analysis. J Raman Spectrosc. 2011;42(6):1248–1254.

[160] Chen H-Y, Zhu SC, Xu HB, Ye MJ, Huang WF, He Y, Qian RC, Li DW. Cell membrane-targeted surface enhanced Raman scattering nanoprobes for the monitoring of hydrogen sulfide secreted from living cells. Biosens Bioelectron. 2024;250: Article 116054. 38295581 10.1016/j.bios.2024.116054

[161] Liu F, Sun Z, Li B, Liu J, Chen Z, Ye J. Surface-enhanced Raman scattering spatial fingerprinting decodes the digestion behavior of lysosomes in live single cells. View. 2024;5(3):20240004.

[162] Chen H-Y, Zhu SC, Xu HB, He Y, Xi CY, Yu JJ, Qian RC, Chen BB, Li DW. A multi-functional core-shell surface-enhanced Raman scattering nanosensor for simultaneous imaging of epidermal growth factor receptor on cell membranes and ROS secreted from living cells. Sensors Actuators B Chem. 2025;422: Article 136636.

[163] Shahalaei M, Azad AK, Sulaiman WMAW, Derakhshani A, Mofakham EB, Mallandrich M, Kumarasamy V, Subramaniyan V. A review of metallic nanoparticles: Present issues and prospects focused on the preparation methods, characterization techniques, and their theranostic applications. Front Chem. 2024;12:1398979. 39206442 10.3389/fchem.2024.1398979 PMC11351095

[164] Cleff C, Gasecka A, Ferrand P, Rigneault H, Brasselet S, Duboisset J. Direct imaging of molecular symmetry by coherent anti-Stokes Raman scattering. Nat Commun. 2016;7(1):11562. 27189667 10.1038/ncomms11562 PMC4873966

[165] Guerenne-Del Ben T, Rajaofara Z, Couderc V, Sol V, Kano H, Leproux P, Petit J-M. Multiplex coherent anti-Stokes Raman scattering highlights state of chromatin condensation in CH region. Sci Rep. 2019;9(1):13862. 31554897 10.1038/s41598-019-50453-0 PMC6761141

[166] Gong L, Zheng W, Ma Y, Huang Z. Higher-order coherent anti-Stokes Raman scattering microscopy realizes label-free super-resolution vibrational imaging. Nat Photonics. 2020;14(2):115–122.

[167] Zong C, Cheng R, Chen F, Lin P, Zhang M, Chen Z, Li C, Yang C, Cheng JX. Wide-field surface-enhanced coherent anti-Stokes Raman scattering microscopy. ACS Photonics. 2022;9(3):1042–1049.

[168] Bera K, Rojas-Gómez RA, Mukherjee P, Snyder CE, Aksamitiene E, Alex A, Spillman DR Jr, Marjanovic M, Shabana A, Johnson R, et al. Probing delivery of a lipid nanoparticle encapsulated self-amplifying mRNA vaccine using coherent Raman microscopy and multiphoton imaging. Sci Rep. 2024;14(1):4348. 38388635 10.1038/s41598-024-54697-3 PMC10884293

[169] Zhitnitsky A, Benjamin E, Bitton O, Oron D. Super-resolved coherent anti-Stokes Raman scattering microscopy by coherent image scanning. Nat Commun. 2024;15(1):10073. 39567553 10.1038/s41467-024-54429-1 PMC11579007

[170] Xiong H, Qian N, Miao Y, Zhao Z, Chen C, Min W. Super-resolution vibrational microscopy by stimulated Raman excited fluorescence. Light Sci Appl. 2021;10(1):87. 33879766 10.1038/s41377-021-00518-5 PMC8058038

[171] He H, Cao M, Gao Y, Zheng P, Yan S, Zhong JH, Wang L, Jin D, Ren B. Noise learning of instruments for high-contrast, high-resolution and fast hyperspectral microscopy and nanoscopy. Nat Commun. 2024;15(1):754. 38272927 10.1038/s41467-024-44864-5 PMC10810791

[172] Horgan CC, Jensen M, Nagelkerke A, St-Pierre JP, Vercauteren T, Stevens MM, Bergholt MS. High-throughput molecular imaging via deep-learning-enabled Raman spectroscopy. Anal Chem. 2021;93(48):15850–15860. 34797972 10.1021/acs.analchem.1c02178 PMC9286315

[173] Abdolghader P, Ridsdale A, Grammatikopoulos T, Resch G, Légaré F, Stolow A, Pegoraro AF, Tamblyn I. Unsupervised hyperspectral stimulated Raman microscopy image enhancement: Denoising and segmentation via one-shot deep learning. Opt Express. 2021;29(21):34205–34219. 34809216 10.1364/OE.439662

[174] Tipping WJ, Faulds K, Graham D. Advances in super-resolution stimulated Raman scattering microscopy. Chem Biomed Imaging. 2024;2(11):733–743. 39610463 10.1021/cbmi.4c00057 PMC11600147

[175] Ruiz-Rodado V, Lita A, Larion M. Advances in measuring cancer cell metabolism with subcellular resolution. Nat Methods. 2022;19(9):1048–1063. 36008629 10.1038/s41592-022-01572-6

[176] Du J, Su Y, Qian C, Yuan D, Miao K, Lee D, Ng AHC, Wijker RS, Ribas A, Levine RD, et al. Raman-guided subcellular pharmaco-metabolomics for metastatic melanoma cells. Nat Commun. 2020;11(1):4830. 32973134 10.1038/s41467-020-18376-x PMC7518429

[177] Dodo K, Sato A, Tamura Y, Egoshi S, Fujiwara K, Oonuma K, Nakao S, Terayama N, Sodeoka M. Synthesis of deuterated γ-linolenic acid and application for biological studies: Metabolic tuning and Raman imaging. Chem Commun. 2021;57(17):2180–2183. 10.1039/d0cc07824g 33527102

[178] Samuel AZ, Miyaoka R, Ando M, Gaebler A, Thiele C, Takeyama H. Molecular profiling of lipid droplets inside HuH7 cells with Raman micro-spectroscopy. Commun Biol. 2020;3(1):372. 32651434 10.1038/s42003-020-1100-4 PMC7351753

[179] Nitta N, Iino T, Isozaki A, Yamagishi M, Kitahama Y, Sakuma S, Suzuki Y, Tezuka H, Oikawa M, Arai F, et al. Raman image-activated cell sorting. Nat Commun. 2020;11:3452. 32651381 10.1038/s41467-020-17285-3 PMC7351993

[180] Hsu C-C, Xu J, Brinkhof B, Wang H, Cui Z, Huang WE, Ye H. A single-cell Raman-based platform to identify developmental stages of human pluripotent stem cell-derived neurons. Proc Natl Acad Sci USA. 2020;117(31):18412–18423. 32694205 10.1073/pnas.2001906117 PMC7414136

[181] Wei Z, Liu W, Yu W, Liu X, Yan R, Liu Q, Guo Q. Multiple parallel fusion network for predicting protein subcellular localization from stimulated Raman scattering (SRS) microscopy images in living cells. Int J Mol Sci. 2022;23(18):10827. 36142736 10.3390/ijms231810827 PMC9504098

[182] Li M, Nawa Y, Ishida S, Kanda Y, Fujita S, Fujita K. Label-free chemical imaging of cytochrome P450 activity by Raman microscopy. Commun Biol. 2022;5(1):778. 35995965 10.1038/s42003-022-03713-1 PMC9395422

[183] Lin Y, Zhang Q, Chen H, Liu S, Peng K, Wang X, Zhang L, Huang J, Yan X, Lin X, et al. Multi-cancer early detection based on serum surface-enhanced Raman spectroscopy with deep learning: A large-scale case–control study. BMC Med. 2025;23(1):97. 39984977 10.1186/s12916-025-03887-5 PMC11846373

[184] Wen Y, Liu R, Xie Y, Li M. Targeted SERS imaging and intraoperative real-time elimination of microscopic tumors for improved breast-conserving surgery. Adv Mater. 2024;36(32):2405253. 10.1002/adma.202405253 38820719

[185] Rehn F, Pils M, Bujnicki T, Bannach O, Willbold D. Artifact detection in fluorescence microscopy using convolutional autoencoder. Sci Rep. 2025;15(1):32482. 40940517 10.1038/s41598-025-18943-6 PMC12432141

[186] Liu T, Liu J, Li D, Tan S. Bayesian deep-learning structured illumination microscopy enables reliable super-resolution imaging with uncertainty quantification. *Nat Commun*. 2025;16:5027. 10.1038/s41467-025-60093-w PMC12125172 40447610

[187] Wang C, Wang D, Kozhevnikov V, Dai X, Turnbull G, Chen X, Kong J, Tang BZ, Li Y, Xu BB. A flexible topo-optical sensing technology with ultra-high contrast. Nat Commun. 2020;11(1):1448. 32193398 10.1038/s41467-020-15288-8 PMC7081276

[188] Bischof J, Fletcher G, Verkade P, Kuntner C, Fernandez-Rodriguez J, Chaabane L, Rose LA, Walter A, Vandenbosch M, van Zandvoort MAMJ, et al. Multimodal bioimaging across disciplines and scales: Challenges, opportunities and breaking down barriers. NPJ Imaging. 2024;2(1):5. 40603654 10.1038/s44303-024-00010-w PMC12118647

[189] Kumari P, Keck S, Sohn E, Kern J, Raedle M. Advanced imaging integration: Multi-modal Raman light sheet microscopy combined with zero-shot learning for denoising and super-resolution. Sensors. 2024;24(21):7083. 39517982 10.3390/s24217083 PMC11548172

[190] Mao J, He H. Deep learning in fluorescence imaging and analysis. J Intell Med. 2024;1(1):42–62.

[191] Mishra V, Kumari N, Vyas M, Aljabali AAA, Chattaraj A, Mishra Y. Advances in multimodal imaging techniques in nanomedicine: Enhancing drug delivery precision. RSC Adv. 2025;15(33):27187–27209. 40740223 10.1039/d5ra03255e PMC12308829

[192] Lukinavičius G, Alvelid J, Gerasimaitė R, Rodilla-Ramirez C, Nguyễn VT, Vicidomini G, Bottanelli F, Han KY, Testa I. Stimulated emission depletion microscopy. Nat Rev Methods Primers. 2024;4(1):56.

[193] van de Linde S, Löschberger A, Klein T, Heidbreder M, Wolter S, Heilemann M, Sauer M. Direct stochastic optical reconstruction microscopy with standard fluorescent probes. Nat Protoc. 2011;6(7):991–1009. 21720313 10.1038/nprot.2011.336

[194] Kamiyama D, Sekine S, Barsi-Rhyne B, Hu J, Chen B, Gilbert LA, Ishikawa H, Leonetti MD, Marshall WF, Weissman JS, et al. Versatile protein tagging in cells with split fluorescent protein. Nat Commun. 2016;7(1):11046. 26988139 10.1038/ncomms11046 PMC4802074

[195] Zhang J, Campbell RE, Ting AY, Tsien RY. Creating new fluorescent probes for cell biology. Nat Rev Mol Cell Biol. 2002;3(12):906–918. 12461557 10.1038/nrm976

[196] Ehtesabi H, Hallaji Z, Najafi Nobar S, Bagheri Z. Carbon dots with pH-responsive fluorescence: A review on synthesis and cell biological applications. Microchim Acta. 2020;187:1–18. 10.1007/s00604-019-4091-4 31989317

[197] Jaiswal JK, Goldman ER, Mattoussi H, Simon SM. Use of quantum dots for live cell imaging. Nat Methods. 2004;1(1):73–78. 16138413 10.1038/nmeth1004-73

[198] Samanta S, Gong W, Li W, Sharma A, Shim I, Zhang W, das P, Pan W, Liu L, Yang Z, et al. Organic fluorescent probes for stochastic optical reconstruction microscopy (STORM): Recent highlights and future possibilities. Coord Chem Rev. 2019;380:17–34.

[199] Bayle V, Fiche JB, Burny C, Platre MP, Nollmann M, Martinière A, Jaillais Y. Single-particle tracking photoactivated localization microscopy of membrane proteins in living plant tissues. Nat Protoc. 2021;16(3):1600–1628. 33627844 10.1038/s41596-020-00471-4

[200] Oleksiievets N, Sargsyan Y, Thiele JC, Mougios N, Sograte-Idrissi S, Nevskyi O, Gregor I, Opazo F, Thoms S, Enderlein J, et al. Fluorescence lifetime DNA-PAINT for multiplexed super-resolution imaging of cells. Commun Biol. 2022;5(1):38. 35017652 10.1038/s42003-021-02976-4 PMC8752799

